# Adherence to community versus facility-based delivery of monthly malaria chemoprevention with dihydroartemisinin-piperaquine for the post-discharge management of severe anemia in Malawian children: A cluster randomized trial

**DOI:** 10.1371/journal.pone.0255769

**Published:** 2021-09-10

**Authors:** Thandile Nkosi-Gondwe, Bjarne Robberstad, Mavuto Mukaka, Richard Idro, Robert O. Opoka, Saidon Banda, Melf-Jakob Kühl, Feiko O. Ter Kuile, Bjorn Blomberg, Kamija S. Phiri

**Affiliations:** 1 School of Public Health and Family Medicine, College of Medicine, University of Malawi, Blantyre, Malawi; 2 Department of Global Public Health and Primary Care, Centre for International Health, University of Bergen, Bergen, Norway; 3 Mahidol-Oxford Tropical Medicine Research Unit, Mahidol University, Bangkok, Thailand; 4 Nuffield Department of Medicine, Centre for Tropical Medicine, University of Oxford, Oxford, United Kingdom; 5 Department of Pediatrics and Child Health, Makerere University College of Health Sciences, Kampala, Uganda; 6 Kenya Medical Research Institute (KEMRI), Centre for Global Health Research, Kisumu, Kenya; 7 Department of Clinical Sciences, Liverpool School of Tropical Medicine, Liverpool, United Kingdom; 8 Department of Clinical Science, University of Bergen, Bergen, Norway; 9 Norwegian National Advisory Unit on Tropical Infectious Diseases, Haukeland University Hospital, Bergen, Norway; University of Tübingen, GERMANY

## Abstract

**Background:**

The provision of post-discharge malaria chemoprevention (PMC) in children recently admitted with severe anemia reduces the risk of death and re-admissions in malaria endemic countries. The main objective of this trial was to identify the most effective method of delivering dihydroartemesinin-piperaquine to children recovering from severe anemia.

**Methods:**

This was a 5-arm, cluster-randomized trial among under-5 children hospitalized with severe anemia at Zomba Central Hospital in Southern Malawi. Children were randomized to receive three day treatment doses of dihydroartemesinin-piperaquine monthly either; 1) in the community without a short text reminder; 2) in the community with a short message reminder; 3) in the community with a community health worker reminder; 4) at the facility without a short text reminder; or 5) at the facility with a short message reminder. The primary outcome measure was adherence to all treatment doses of dihydroartemesinin-piperaquine and this was assessed by pill-counts done by field workers during home visits. Poisson regression was utilized for analysis.

**Results:**

Between March 2016 and October 2018, 1460 clusters were randomized. A total of 667 children were screened and 375 from 329 clusters were eligible and enrolled from the hospital. Adherence was higher in all three community-based compared to the two facility-based delivery (156/221 [70·6%] vs. 78/150 [52·0%], IRR = 1·24,95%CI 1·06–1·44, p = 0·006). This was observed in both the SMS group (IRR = 1·41,1·21–1·64, p<0·001) and in the non-SMS group (IRR = 1·37,1·18–1·61, p<0·001). Although adherence was higher among SMS recipients (98/148 66·2%] vs. non-SMS 82/144 (56·9%), there was no statistical evidence that SMS reminders resulted in greater adherence ([IRR = 1·03,0·88–1·21, p = 0·68). When compared to the facility-based non-SMS arm (control arm), community-based delivery utilizing CHWs resulted in higher adherence [39/76 (51·3%) vs. 54/79 (68·4%), IRR = 1·32, 1·14–1·54, p<0·001].

**Interpretation:**

Community-based delivery of dihydroartemesinin-piperaquine for post-discharge malaria chemoprevention in children recovering from severe anemia resulted in higher adherence compared to facility-based methods.

**Trial registration:**

NCT02721420; ClinicalTrials.gov.

## Introduction

Severe anemia in under-five children is defined as low hemoglobin level of less than 7g/dl [1]. It affects over 10 million children globally and is one of the leading cause of pediatric hospital admissions and mortality in sub-Saharan Africa (SSA) [2]. Children admitted to hospital with severe anemia are at high risk of dying not only during the acute phase but also after discharge from hospital [3,4]. One prospective study in Malawi reported that 8% of hospitalized children with severe anemia were re-admitted or died within six months after discharge compared to none of the community controls [5]. Post-discharge mortality rates as high as 17·9% and 36·5% have been reported in other African countries such as Kenya and Uganda [4,6]. In malaria endemic African countries, malaria infection is a major contributor of severe anemia and is a risk factor for slow hematological recovery that occurs in the community where the risk remains high [7–10].

Post-discharge malaria chemoprevention (PMC) is the targeted use of antimalarials in children with severe anemia to create a malaria prophylactic window period post-transfusion and during recovery [11]. Similar to seasonal malaria chemoprevention (SMC), PMC clears existing infections and provides prolonged prophylaxis against new infections [12–15]. A randomized clinical trial conducted in Malawi reported that provision of PMC to children aged less than 5 years with long-acting ACTs during recovery from severe anemia prevented up to 21% of deaths or hospital readmissions within 6 months after discharge [16]. More recently trials in Uganda and Kenya found that PMC resulted in 65% reduction in malaria incidence during the three months intervention period after successful treatment with blood transfusion and parenteral antimalarial drugs [11,17].

Utilizing community health workers (CHW) to deliver interventions for childhood diseases such as pneumonia and malaria has been reported to be effective in reducing childhood mortality and improved access [18]. Compared to facility-based delivery, provision of intermittent preventive therapy (IPT) in pregnant women and children by CHWs in the community, has also been shown to reduce malaria prevalence and incidence [19,20]. However, this delivery strategy may have some challenges with sustainability due to inadequate supervision, low remuneration and irregular supplies among others [21]. Although facility based interventions have been effective at reducing disease complications [19], they are costly and challenging to deliver for the health system [22].

PMC is an intervention that could offer substantial public health gains in managing under-five children who are at risk of dying. Although PMC is a relatively simple intervention, implementation will require appropriate delivery strategies so that it reaches the population that need it most. However, a major concern is how it can be delivered in an effective and sustainable way [16]. However, unlike other health interventions delivered to children through established health systems such as the expanded programme on immunization, there is no existing delivery mechanism for PMC.

The aim of this trial was to identify the most effective delivery mechanism for PMC by comparing community and facility-based delivery systems with and without reminders using short-text message service (SMS) and using CHWs also known as health surveillance assistants (HSAs) in Malawi.

## Methods

### Study site

**Fig 1:**
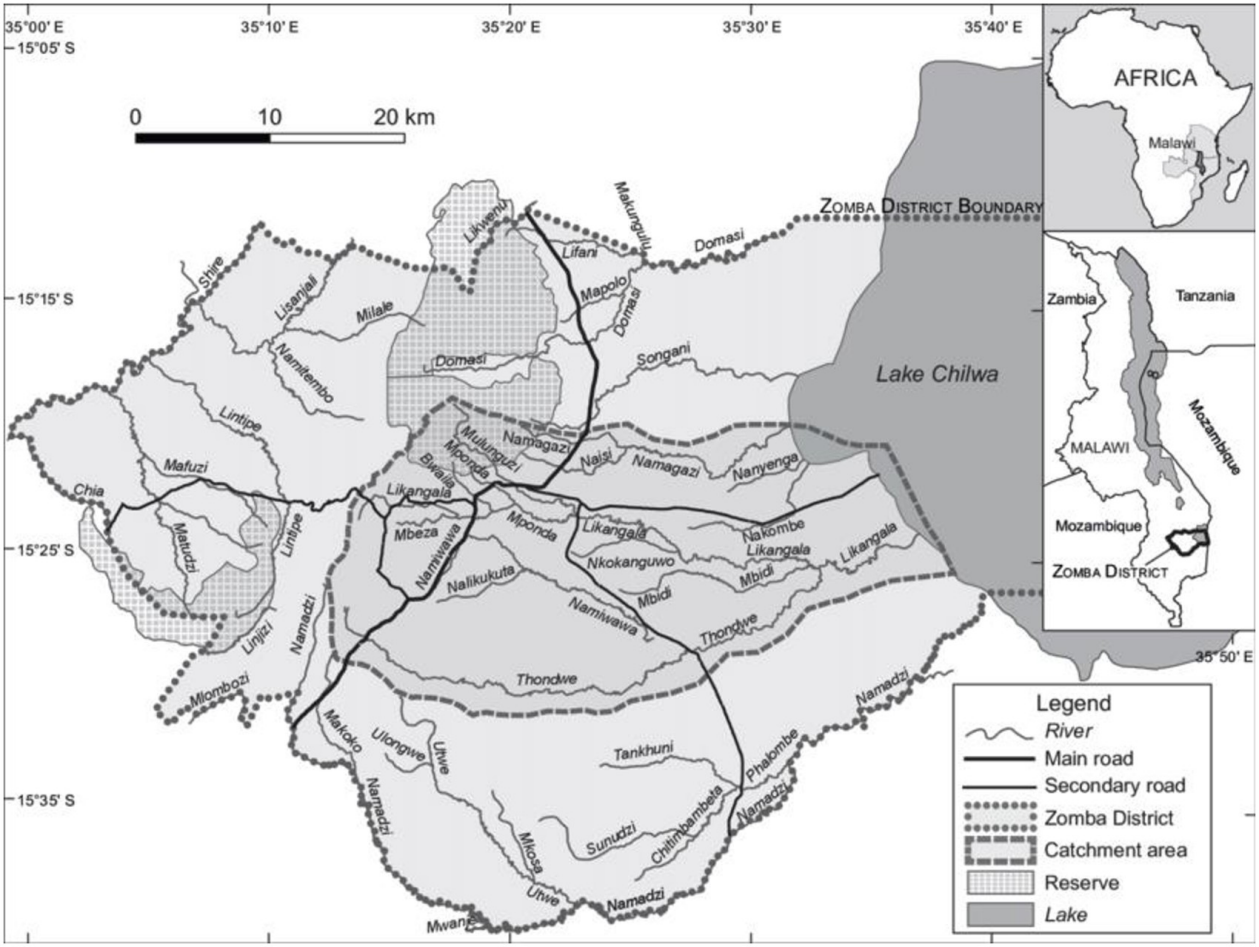
A map of Zomba depicting the study site catchment area.

The study was conducted at Zomba central hospital (ZCH), a referral government hospital with high pediatric admissions rates due to severe malarial anemia located in southern Malawi with perennial malaria transmission. The hospital provides health services to a population of over one million and it is the only government health facility that provides blood transfusions and health services in Zomba district free of charge. Zomba Central Hospital was selected because previous related studies had been conducted there and is was particularly suitable for the delivery trial because of the existing linkages with community based health care providers and rural clinics within their catchment areas. Fig 1 is a map depicting the study area.

### Study design and participants

This was a five-arm open label cluster-randomized trial. Children aged less than five years who were admitted to ZCH pediatric ward were included if they had severe anemia (defined as a hemoglobin (Hb) concentration of <5g/dL) [23], and having received the standard in-hospital care which included a blood transfusion, intravenous artesunate and antibiotics, if bacterial infection was suspected. Once stable, all children received oral lumefantrine-artemether (AL) and caregivers were provided with information regarding the trial procedures, medication and follow up. We excluded children with other specific causes of anemia such as trauma, those with confirmed sickle cell or heart disease and those who resided out of the catchment area. After obtaining informed consent in the local language of Chichewa from their legal guardians, we collected socio-demographic information and medical history using questionnaires and performed a physical examination. We collected a blood sample from a finger prick to check Hb using the hemocue 301 (Angelholm, Sweden) and malaria slides for microscopy. After enrolment, the study participants were traced to their home to collect GPRS positions for follow up and ascertain distance from the hospital.

### Randomization and procedures

The unit of randomization was the village cluster and it was computer-generated by the trial statistician prior to the trial. All of the 1486 villages which were defined to be in the catchment area of ZCH were randomized to each delivery method, as it was not known which village prospective participants would reside prior to the study. Therefore, all children from the same village cluster would be assigned to the same delivery arm. The villages were randomized to one of the following five arms: 1) community-based without an SMS reminder (Com + No SMS); 2) community-based with an SMS reminder (Com+SMS); 3) community-based with an HSA reminder instead of SMS reminder (Com+HSA); 4) facility-based delivery without SMS reminder (Fac-SMS); or 5) facility-based delivery with SMS reminder (Fac+SMS). Detailed descriptions of the methods have been provided in Table 1 and published (S4 File) [24].

**Table 1:** Detailed description of delivery methods of PMC.

Method	Arm	Description
Community-based	Com+ No SMS	PMC drugs given at discharge without SMS reminders: The guardian received all drugs for PMC-1, PMC-2, and PMC-3. They were instructed on how and when to give these drugs to the child when being discharged from the hospital.
	Com+SMS	PMC drugs given at discharge with SMS reminders: The guardian received all drugs for PMC-1, PMC-2 and PMC-3 and was instructed on how and when to give the drugs to the child. Additionally, they were reminded via SMS to give the drugs to the child a day before each treatment course was due.
	Com+HSA	PMC drugs given at discharge with HSA reminders: The guardian receives all drugs for PMC-1, PMC-2 and PMC-3 and was instructed on how and when to give the drugs to the child. Additionally, HSAs who are officially government-supported community-based health workers were reminded via SMS to go and remind the guardian to give the drugs to the child a day before each treatment course was due.
Facility-based	Fac+ No SMS	PMC drugs collected from the hospital: At discharge, the guardian was requested to return to the outpatient department (OPD) of the hospital each month to collect drugs (a treatment course at each time) for PMC-1, PMC-2, and PMC-3. They did not receive any form of reminder other than what was documented in the child’s health card.
	Fac+SMS	PMC drugs collected from the hospital with SMS reminders: At discharge, the guardian was requested to return to the OPD each month to collect drugs (a treatment course at each time) for PMC-1, PMC-2, and PMC-3. Additionally, they were reminded via SMS to come to the clinic to collect drugs a day before each treatment course was due.

#### Text message reminder

In the SMS arms, the text of the SMS was customized for each child with the following message “Remember to give (child’s name) medication starting from tomorrow for three days.” It was translated into the local language of Chichewa, the most widely spoken language in Malawi. The reminder was pre-tested on a random sample of caregivers coming with their children to the pediatric out-patient department prior to commencement of the trial. Considering that this was an implementation trial, we wanted to reflect the “real life” situation and therefore mobile phones were not provided to caregivers. However, for all study participants; we collected mobile phone numbers of the nearest contact person. Consenting caregivers were instructed to inform the owners of the mobile phone numbers that their child was participating in the trial Caregivers and study staff was aware of the allocation. However, the study statistician who performed the analyses was blinded to treatment allocation.

### Study medication

The study medication was co-formulated 20 mg dihydro-artemisinin and 160 mg piperaquine (Eurartesim^®^, Sigma-Tau, Italy) dispersible tablets prescribed in standard doses for three days every month. The monthly treatment courses were prescribed at two (PMC-1), six (PMC-2) and ten weeks (PMC-3) after discharge enrolment regardless of the presence of symptoms. The maximum number of doses that could be administered was nine. Standardized verbal instructions about administration were given to the primary caregiver and specific dates of administration were documented in the child’s health book and the drug blister pack. After enrolment, all the participants were requested to come back to the study clinic at 15 weeks for the end of study assessment.

#### Follow-up procedures

One to three days after the child was supposed t be given the last dose of each course of medication, field workers made unannounced home visits to collect blister packs and assess if study medication was indeed given to the child participating in the trial. If the caregiver was not at home, they were contacted and visits were made when they were at home. The number of tablets remaining in the blister packs was recorded and if none were remaining “0” was recorded. If the blister pack was missing the field worker probed to assess if the medicine was given to the child or not. In addition, assessment for adverse events was made and children were referred to the nearest health facility if they were sick. Data were entered directly into questionnaires in handheld tablets programmed with open data kit (ODK) electronic case report forms (eCRF). Stata 15 was used for analysis.

### Sample size

The study was designed to determine whether delivery of PMC through community-based methods resulted in higher adherence than when delivered through facility-based methods and the sample size was based on this. The main effects of “place of delivery” and “use of mobile phone reminders” were of primary interest. It was estimated that each village cluster would contribute 2 to 4 children with severe anemia. We assumed an intra-cluster correlation coefficient (ICC) of 0.1 and allowing for 20% loss-to-follow-up or efficiency loss due to varying cluster sizes, a sample size of 125 clusters (villages) of an average of 3 children per village (i.e. 75 children per arm and 375 overall), had 80% power to detect a 25% absolute increase in coverage from an estimated 50% in the standard out-patient groups to 75% in the arms supported by mobile phone reminders (α = 0.05). The ICC of 0.1 is slightly more conservative than the ICC in a previous trial of delivery approaches for IPTc in the Gambia (0.08). A statistical analysis plan (SAP) was written and agreed upon by all investigators before the data were analyzed. However, additional analyses were included in the SAP during analysis to explore effects of different cut offs of adherence.

### Statistical considerations

We used appropriate descriptive statistics to summarize data, with mean or median (IQR) for continuous data, and frequencies and percentages for categorical data. For the primary outcome, our analysis was by intention to treat at cluster and participant levels. We used Poisson regression for the total number of doses administered. The unadjusted and adjusted incidence rate ratios (IRR) measuring the percentage of children receiving PMC according to schedule in each arm were obtained and compared between arms and the 95% CI for the IRR have been reported. The estimates of the IRR were adjusted for baseline characteristics and accounted for clustering with 95% CIs are provided for all measures of effect.

We also employed a factorial design analysis for further analysis to investigate the interaction between the effects of community compared to facility delivery and the pooled effect of SMS; and interaction terms were included in models to assess the strength of interaction. We also did several subgroup analyses for the primary and secondary outcomes adjusted for clustering, socioeconomic and demographic variables stratified by each intervention arm with the Fac-SMS group as the reference group.

Our primary outcome of the study adherence to 7 to 9 doses of the study drugs. The secondary outcomes of the trial were: 1) the proportion of those with medium adherence defined as administration of 4 to 6 doses of the study drug; 2) the proportion of those with low adherence defined as the administration of less than 3 doses of the study drugs; 3) all-cause mortality and 4) all cause sick visits.

For the secondary outcomes, the levels of adherence were compared between the five groups. Full details of the study design and statistical methods are provided in detail in the protocol [24], which is available as S4 File.

### Ethical approval

The study was approved by the research ethics committees of the College of Medicine in Malawi (COMREC, approval number P·02/15/1679 and the Regional Ethics Committee of Norway, approval number 2015/537 (REK Vest). Written informed consent was obtained from legal guardians of the study participants prior to enrolment. The trial was registered at ClinicalTrials.gov (identifier: NCT02721420).

## Results

**Fig 2:**
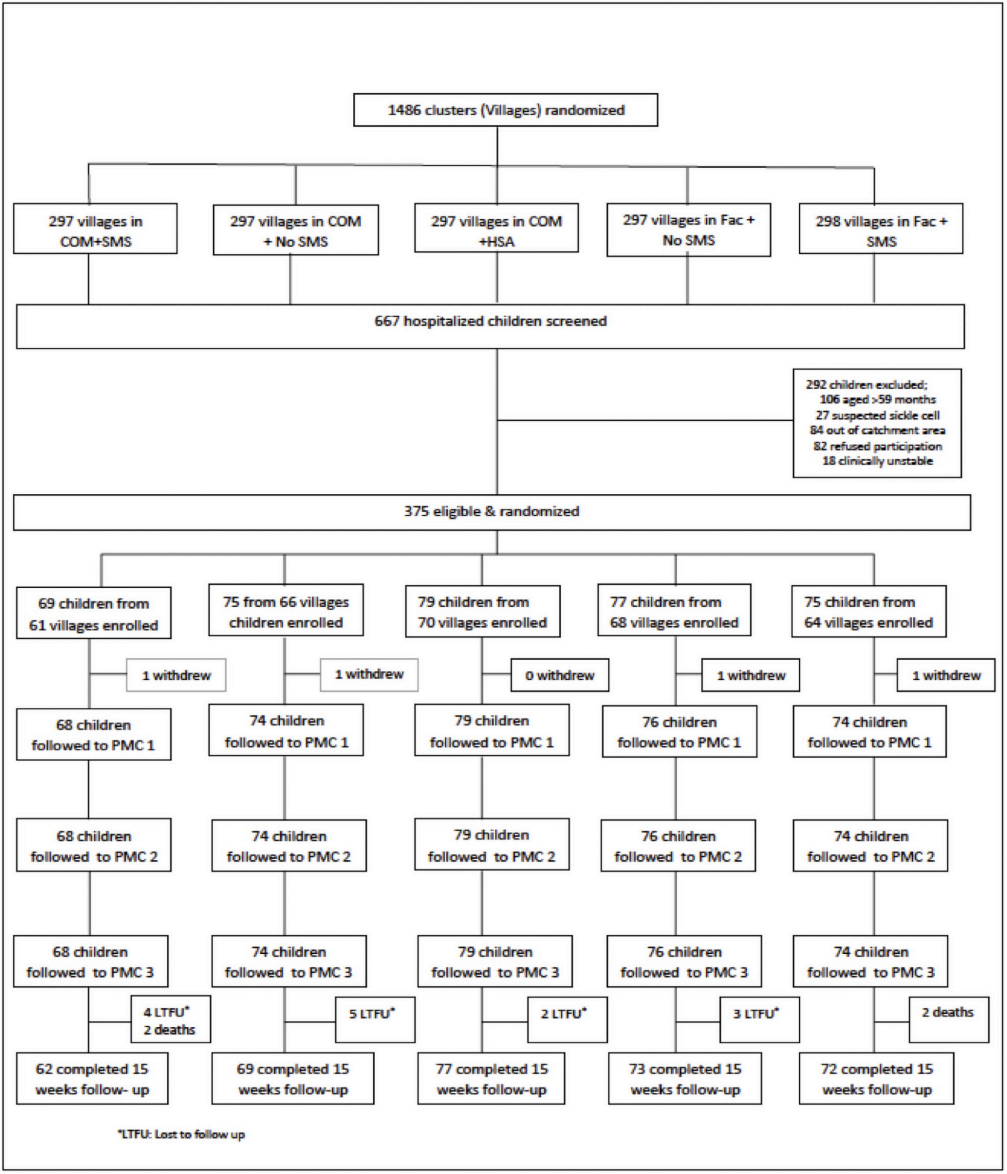
Consolidated Standards of Reporting Trials (CONSORT) flow diagram of the PMC delivery trial.

Between 24 March 2016 and 09 October 2018, a total of 667 children hospitalized with severe anemia were screened for eligibility and 375 children from 329 villages were eligible and enrolled (Com+ No SMS = 69; Com+SMS = 75; Com+HSA = 79; Fac-SMS = 77; Fac+SMS = 75). A total of 351 children (93.6%) were followed successfully until the end of the study; caregivers of four children (1.1%) withdrew consent prior to the first home visit while four children died and twenty did not attend the end of study visit at 15 weeks. Fig 2 is a CONSORT flow chart depicting the enrolment and follow up procedures of the trial.

Table 2 provides a summary of the baseline characteristics of the trial participants. There was a good balance in the baseline characteristics across the arms. The overall median age and weight for all the participants were 29 (IQR 19–39) months and 11.3 (IQR 9.6–13.2) kg, respectively. There were generally more males in each arm except in the Fac+SMS arm (48% male). The proportion of participants with a history of having had a blood transfusion in the preceding month to enrolment was low (1.6%) across all the arms (Table 1). Over three quarter of children used insecticide-treated nets (ITN) the previous night prior to admission. None of the households had insecticide residual spraying. Fever was the most common presenting symptom and the mean hemoglobin was 7.9g/dL (sd 1.4).

**Table 2:** Baseline characteristics of trial participants.

Characteristic	Com+ No SMS	Com+SMS	Com+HSA	Fac+ No SMS	Fac+SMS	Overall
	N = 69	N = 75	N = 79	N = 77	N = 75	N = 375
Participant age in months, median (IQR)	30 (19–40)	30 (23–38)	28 (18–39)	29 (20–38)	28 (16–38)	29 (19–39)
Weight in kg, median (IQR)	12.0 (9.9–3.9)	11.4 (10.0–13.5)	11.4 (9.9–12.5)	11.0 (9.9–12.7)	11.3 (9.6–13.2)	11.3 (9.6–13.9)
Height in cm, median (IQR)	86 (78–92)	84 (79–89)	84 (75–91)	83 (77–90)	84 (77–91)	84 (77–91)
Male, n (%)	37 (53.6)	48 (64.0)	49 (62.0)	42 (54.6)	36 (48.0)	212 (56.5)
Previous transfusion, n (%)	1 (1.5)	2 (2.7)	0 (0.0)	1 (1.4)	2 (2.7)	6 (1.6)
Malaria in previous month n (%)	10 (14.5)	9 (12.2)	6 (7.5)	5 (6.9)	4 (5.5)	34 (9.2)
Slept under ITN previous night, n (%)	52 (75.4)	54 (72.0)	64 (81.0)	62 (80.5)	55 (73.3)	287 (76.5)
Diarrhoea on admission, n (%)	11 (15.9)	12 (16.2)	18 (22.7)	15 (20.6)	14 (19.2)	70 (19.0)
Fever on admission, n (%)	63 (91.3)	69 (92.0)	74 (93.7)	60 (78.0)	69 (92.0)	335 (89.3)
Vomiting on admission, n (%)	26 (37.7)	24 (32.4)	31 (39.2)	17 (23.3)	29 (39.7)	127 (34.5)
Received at three doses of parenteral artesunate, n (%)	69 (100)	75 (100)	79 (100)	77 (100)	75 (100)	375 (100)
Difficulty taking medication, n (%)	14 (22.2)	22 (34.4)	21 (28.7)	19 (29.7)	14 (21.5)	90 (27.4)
Hb at enrolment in g/L, mean (Sd)	7.7 (1.3)	7.9 (1.5)	8.1 (1.6)	7.9 (1.3)	8.1 (1.3)	7.9 (1.4)
Hb at end of study in g/L, mean (Sd)	11.3 (1.5)	11.7 (1.4)	11.6 (1.4)	11.5 (1.5)	11.3 (1.8)	11.5 (1.5)
Guardian age in years, median (IQR)	28 (23–36)	26 (22–34)	27 (23–33)	27 (23–35)	27 (22–34)	27 (22–34)
Literate legal guardian, n (%)	50 (72.5)	46 (63.0)	54 (68.4)	50 (67.6)	52 (72.6)	253 (68.8)
Ownership of mobile phone, n (%)	28 (40.6)	28 (38.4)	28 (35.4)	29 (39.2)	33 (45.2)	146 (39.7)
Wealthy SES, n (%)	10 (14.5)	16 (21.6)	13 (16.5)	15 (20.6)	19 (26.0)	73 (19.8)
Distance to hospital in km, mean (Sd)	19.6 (8.9)	19.4 (8.9)	19.5 (10.2)	20.5 (8.5)	18.2 (9.5)	19.7 (9.2)

Mobile phone ownership in the household or with a neighbor was reported in 39.7% of the caregivers, with the Fac+SMS arm reporting highest (45.2%). The mean distance to the hospital was similar among all the arms (19.7 ±SD 9.5 km). Out of the 150 participants who were expected to receive SMS reminders, only 25 [18.0% (10 in COM+SMS and 15 in FAC+SMS)] reported to have received at least one SMS.

### Adherence

The primary outcome was to compare the PMC delivery across the five intervention arms. The endpoint is based on the total number of doses that were actually used. Adherence to all PMC courses was reported in 63.1% of all the children. A higher proportion of children in the community-based arms reported full adherence to compared to facility-based arms (70.6% versus 52.7%, IRR = 1.24, 95% CI 1.06–1.44, p = 0.006). Children receiving PMC in the community in the SMS arm reported the highest proportion of full adherence (79.7%) compared to those in the community but receiving no SMS (63.2%), Com+HSA (68.4%), Fac-SMS (51.3%) and Fac+SMS (52. %) in the univariate analysis. The proportion that was administered at least six doses representing medium adherence was 22.1% among all the children and Fac-SMS arm had the highest proportion of medium adherence (27.6%), and Com+SMS arm (12.2%) lowest for medium adherence. On the other hand, only 2.9% of the children reported very low adherence which was representing less than 30% of the total doses (Table 3, S2 and S3 Files).

**Table 3:** Levels of adherence to DHP across all the trial arms.

Level of adherence	Total doses	Com+ No SMS n (%)	Com+ SMS n (%)	Com+ HAS n (%)	Fac+ No SMS n (%)	Fac+ SMS n (%)	Total n (%)
Very low (< 30% uptake)	0	1 (1.5)	2 (2.7)	1 (1.3)	4 (5.3)	2 (2.7)	10 (2.7)
	1	0 (0)	0 (0)	1 (1.3)	0 (0)	0 (0)	1 (0.3)
	2	0 (0)	0 (0)	0 (0)	0 (0)	0 (0)	0 (0)
	**Total**	**1 (1.5)**	**2 (2.7)**	**2 (2.6)**	**4 (5.3)**	**2 (2.7)**	**11 (2.9)**
Low (30% uptake)	3	7 (10.3)	4 (5.4)	6 (7.6)	11 (14.5)	13 (17.6)	41 (11.1)
	4	0 (0)	0 (0)	0 (0)	0 (0)	0(0)	0 (0)
	5	1 (1.5)	0 (0)	0 (0)	1 (1.3)	1 (1.4)	3 (0.8)
	**Total**	**8 (11.8)**	**4 (5.4)**	**6 (7.6)**	**12 (15.8)**	**14 (18.9)**	**44 (11.9)**
Medium (60% uptake)	6	15 (22.1)	9 (12.2)	16 (20.3)	21 (27.6)	18 (24.3)	79 (21.3)
	7	1 (1.5)	0 (0)	0 (0)	0 (0)	1 (1.4)	2 (0.5)
	8	0 (0)	0 (0)	1 (1.3)	0 (0)	0 (0)	1 (0·3)
	**Total**	**16 (23.5)**	**9 (12·2)**	**17 (21.5)**	**21 (27.6)**	**19 (25.7)**	**82 (22.1)**
Full (100% uptake)	9	43 (63.2)	59 (79.7)	54 (68.4)	39 (51.3)	39 (52.7)	234 (63.1)
	**Total**	**43 (63.2)**	**59 (79.7)**	**54 (68.4)**	**39 (51.3)**	**39 (52.7)**	**234 (63.1)**
**Total**		68 (100)	74 (100)	79 (100)	76 (100)	74 (100)	371 (100)

In the mixed effects model, village did not have a significant random effect, the likelihood ratio test was 2.9e-05, p = 0.498. In fact the Intra-Cluster Correlation coefficient (ICC) was very small 7.56e-06, indicating that there were no correlations for individuals coming from the same village relative to those of different villages. Thus, the available data is as good as the independent data and give the same results as independent data analysis.

**Fig 3:**
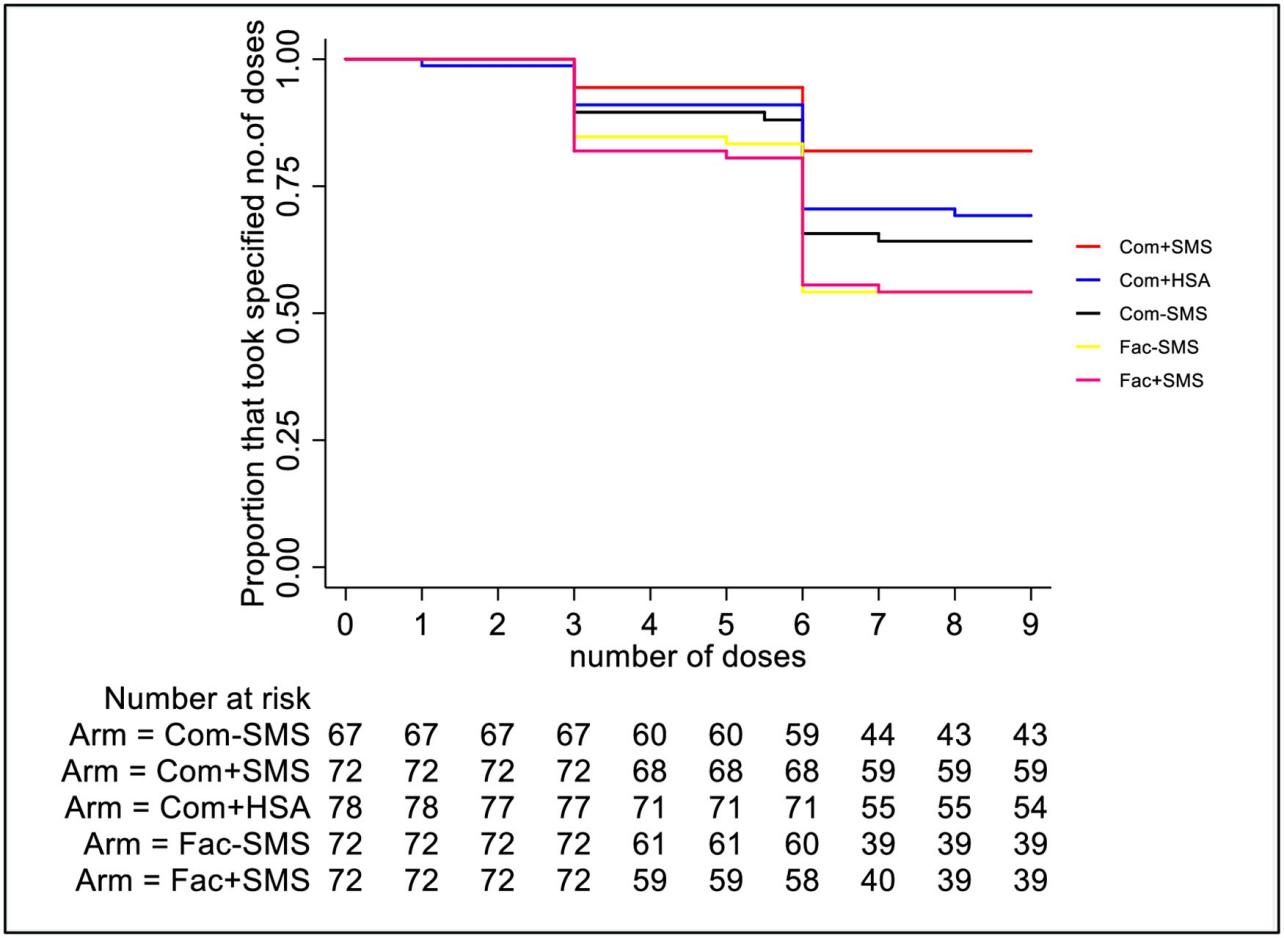
Kaplan Meir curves illustrating the adherence by the total number of PMC doses that were administered in each trial arm.

**Fig 4:**
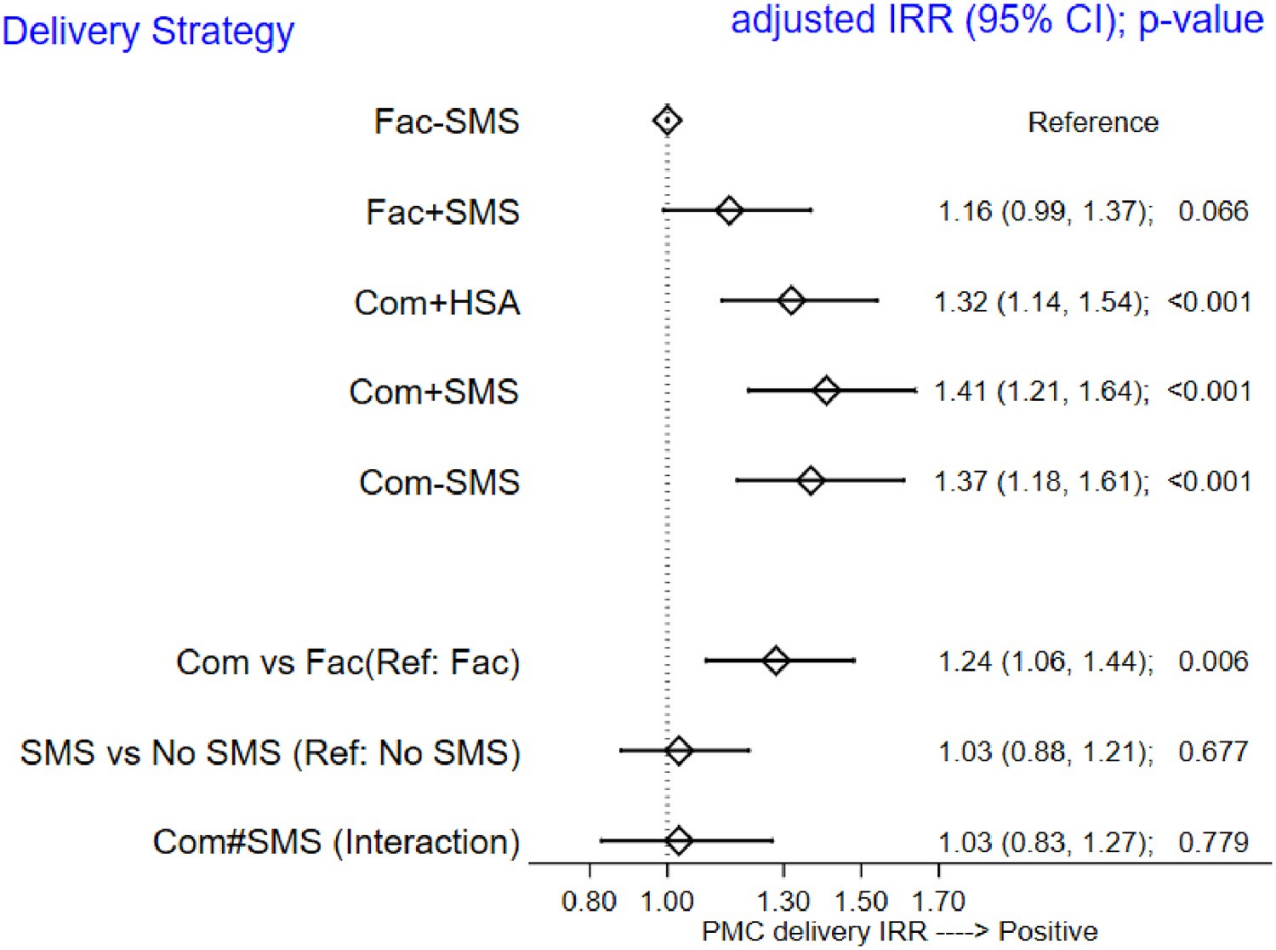
Forest plots illustrating adherence to PMC in each trial arm.

When modelled as count data to assess whether the incidence of number of doses used per patient differ across the groups, a mixed effects Poisson regression was fit to the data. When PMC was delivered through the community-based methods, adherence was greater than facility-based methods (IRR = 1.24; 95%CI 1.10, 1.44 p = 0.001) and this was observed in both the SMS recipients (IRR = 1.41; 95%CI 1.21, 1·64, p<0.001) and in the non-SMS recipients (IRR = 1.37; 95%CI 1.18, 1.61, p<0.001) (Figs 3 and 4, Table 4).

**Table 4:** Poisson regression model excluding PMC 1 with an interaction term in factorial design analysis for adherence between a) community compared to facility and b) SMS compared to no SMS.

Strategy		Crude		Adjusted	
		IRR (95% CI)	p-value	IRR (95% CI)	p-value
Pooled	Facility	Reference	-	1	-
	Community	-	-	1.24 (1.06, 1.44)	0.006
	No SMS	Reference	-	1	
	SMS	-		1.03 (0.88, 1.21)	0.677
By arm	Fac+ no SMS (Ref)	Reference	-	1	-
	Com+ No SMS	1.33 (1.14,1.54)	<0.001	1.37 (1.18,1.61)	<0.001
	Com+SMS	1.37 (1.19,1.59)	<0.001	1.41 (1.21,1.64)	<0.001
	Com+HSA	1.29 (1.12,1.5)	0.001	1.32 (1.14,1.54)	<0.001
	Fac+SMS	1.12 (0.96,1.31)	0.140	1.16 (0.99,1.37)	0.066

Overall, a total of 83.6%, 84.1% and 78.0% of the children received complete courses of PMC1, PMC2 and PMC3 respectively. Generally, most of the children who received the first dose of each PMC course also received the second and the third dose. A small proportion (1.3%) received the first and second dose but not the third dose of PMC 1, only one participant (0·3%) took only the first dose of PMC 2 and another one (0.3%) took only the first and second doses of PMC 2. All participants who took the first course of PMC 3 completed all the three doses.

Community-based delivery arms had the highest proportions of participants receiving PMC2; (86.4 to 91.9%) and PMC3 (78.4 to 89.4%) compared to facility-based arms; who reported up to 78.7% receiving PMC2 and less than 70% for PMC 3. There were no significant differences in the proportion of participants across the arms in administration taking the PMC1 (p = 0·56) and PMC2 (p = 0·08) but administration of PMC 3 was statistically different across all the groups.

As shown in Figs 3 and 4, community delivery utilizing HSA reminders resulted in higher adherence compared to delivery in the health facility (IRR = 1.32; 95%CI 1.14, 1.54, p<0.001). However, there was no evidence that SMS reminders resulted in greater adherence compare to the arms that did not receive the SMS (IRR = 1.03; 95%CI 0.88, 1.21, p = 0.7.

There was no difference in adherence between facility-based delivery with SMS and without SMS (IRR = 1.16; 95%CI 0.99,1.37, p = 0.07) (Fig 3) and there was no evidence of interaction between the use of SMS and delivery strategy (Facility/community) (p = 0.88).

There were total of four deaths (overall mortality of 1.1%); two each in the Com-SMS and Fac+SMS. Two deaths were caused by severe anemia; one due to sepsis and one death caused by drowning. The total number of reported sick visits was 90 of which 46.7% were due to uncomplicated malaria. The lowest number of adverse events was observed in the Com+HSA arm. The number of deaths was too low to make meaningful association to adherence.

## Discussion

In this study, we found that children who had their monthly DHP in the community provided by caregivers irrespective of reminders resulted in 24% higher adherence compared to facility-based delivery where caretakers were asked to return to the clinic to collect their 2^nd^ and 3^rd^ course of PMC. These findings are consistent with findings reported from other African countries. Studies in Kenya, Uganda and Ghana reported that malaria treatment distributed by CHWs resulted in reduced malaria incidence and increased access to treatment [22,25,26]. Similarly, in Malawi, community delivery of sulfadoxine-pyrimethamine (SP) for intermittent preventive therapy in pregnancy IPTp by CHWs improved coverage from 41.5% to 82.9% however it was noted that this led to a reduction in antenatal care (ANC) attendance [27]. In the Gambia, delivery of intermittent preventive therapy in children (IPTc) by CHWs also achieved higher coverage, cost-effectiveness and reduced symptomatic malaria cases [20].

Although utilizing CHWs has been supported by WHO and has proved to be effective for high adherence and reduced incidence of disease, the effectiveness has shown to decline for subsequent doses. In Mali, seasonal malaria chemoprevention (SMC) delivered through routine programmes utilizing existing CHWs reduced malaria prevalence; 84% of the targeted children received the first course but only 54% received four complete SMC courses [28]. Another study reported that completion of all the three doses of IPTc was similar (91.6% versus 91.7%) in community delivery compared to delivery at health centers [29]. Utilizing HSAs has its own challenges and requires additional resources for training, supervision and incentives and this may be challenging for PMC delivery [20,30]. In addition, children receiving PMC will not be in the scheduled work plans for HSAs to incorporate. In our setting, it has been reported that HSAs are motivated to provide reminders for PMC [31]. However, despite this motivation, our study still showed that less than half of the HSAs conducted the required home visit reminders. Many of the reported challenges include inability to locate the child, high existing workload, limited training, supervision, and poor community acceptance [31,32].

Whereby there are limited studies that have examined adherence to medication when utilizing caregivers to administer longer-term malaria interventions at home, in our study, we found that community-delivery utilizing caregivers resulted similar adherence without requiring HSA reminders. One study in Sierra Leone reported that caregivers are capable of administering anti-malarial medication to the children with basic instructions and supervision of the first dose by the CHW [33]. Caregivers were able to administer monthly doses without additional reminders and this is important because it does not require additional resources. A qualitative study examining the acceptance of PMC among caregivers in Malawi reported that caregivers had a higher preference and confidence to be given all the medication to administer to their children without any additional reminder because HSAs and phones were unreliable and collecting medication from the hospital was very challenging [34]. However, the concern with this method is poor storage conditions, ensuring that medication will be only be administered as prescribed to the child and not others and possible side effects of the drugs [34].

Use of mobile phone reminders has been hailed as an important tool to achieve adherence, optimal treatment response, case management and outcomes in resource poor settings such as SSA [35,36]. However, we found that SMS did not have that added effect on adherence. In rural Malawi, provision of SMS to health workers or mothers has been reported to not have a significant effect on adherence and improvement of management of common childhood illnesses with only up to 46·5% receiving SMSs [37,38]. This is rather low compared to similar trials in Kenya where providing SMS was effective when 91% received them [39,40]. Challenges reported with implementing mobile health (mhealth) interventions in resource poor settings such as Malawi include poor network coverage, access, and inability to charge mobile phones, literacy and low phone ownership [41].

Our findings are important because PMC is a relatively new intervention in the Malawian and other developing countries health care system where established delivery systems currently do not exist. Because the timing of PMC medication administration is sensitive, it would be a challenge for a caregiver to rely on receiving timely reminders on a personal phone or rely on others to receive the reminder and pass on the message. A study in Tanzania reported that unsupervised malaria treatment given at home by caregivers was effective without the utilization of any reminders [30]. Contrasting to this, caregivers have a higher preference and confidence to be given all PMC medication to administer at home without any reminders [34]. In this case, community-based delivery strategies where the medication is given to the caregivers may be the most successful strategy for this promising intervention.

Although we found that SMS reminders had no effect on adherence, it must be noted that the proportion of caregivers that reported received the SMS reminder was very low. This was a major limitation in our study as we were not able to conclusively state that they are not effective in improving adherence regards to PMC in our setting.

## Conclusion

This is the first study to assess adherence to PMC, which is an innovative intervention for managing sick children during the post-discharge period. We found that using community-based strategies, caregivers were able to administer PMC medication to their children without additional reminders. As they routinely administer medication to their children at home, this may be a cost-effective strategy for delivery of PMC. Although utilizing HSAs has proven to be effective for improving adherence for other malaria interventions, adherence to subsequent doses has tended to decline and may remain challenging for PMC delivery utilizing HSAs reminders, which is interval sensitive.

## Supporting information

S1 Checklist CONSORT 2010 checklist of information to include when reporting a cluster randomised trial. (DOCX)

S1 File Dataset. (XLS)

S2 File Table 4. Description of the total number of doses administered for each course of PMC. (DOCX)

S3 File Table 5. The distribution of the timing of each dose of PMC per course administered in each trial arm. (DOCX)

S4 File Trial protocol. (PDF)

S5 File PMC trial Malawi protocol for PLOS One. (PDF)

S6 File Database anonymised. (XLS)
