# Supplementary material for: Adherence to community versus facility-based delivery of monthly malaria chemoprevention with dihydroartemisinin-piperaquine for the post-discharge management of severe anemia in Malawian children: A cluster randomized trial
Source: PLoS One. 2021 Sep 10;16(9):e0255769. doi: 10.1371/journal.pone.0255769 (PMC8432777; doi:10.1371/journal.pone.0255769)
Supplement: S2 File — (DOCX) [file pone.0255769.s003.docx]

S2_Table 4. Description of the total number of doses administered for each course of PMC

| **Course** | **No. of doses** | **Com+ No SMS**  **n/N (%)** | **Com+SMS**  **n/N (%)** | **Com+HSA**  **n/N (%)** | **Fac+ No SMS**  **n/N (%)** | **Fac+SMS**  **n/N (%)** | **Total**  **n/N (%)** |
| --- | --- | --- | --- | --- | --- | --- | --- |
|  | 0 | 15/68 (22.1) | 11/74 (14.9) | 10/79 (12.7) | 10/76 (13.2) | 10/74 (13.5) | 56/371 (15.1) |
| PMC 1 | 1 | 0/68 (0) | 0/74 (0) | 1/79 (1.3) | 0/76 (0) | 0/74 (0) | 1/371 (0.3) |
|  | 2 | 1/68 (1.5) | 0/74 (0) | 0/79 (0) | 1/76 (1.3) | 2/74 (2.7) | 4/371 (1.1) |
|  | 3 | 52/68 (76.5) | 63/74 (85·1) | 68/79 (86·1) | 65/76 (85·5) | 62/74 (83·8) | 310/371 (83·6) |
|  | 0 | 7/66 (10.6) | 4/74 (5.4) | 10/79 (12.7) | 17/76 (22.3) | 16/73 (21.6) | 56/368 (15.1) |
| PMC 2 | 1 | 1/66 (1.5) | 0/74 (0) | 0/79 (0) | 0/76 (0) | 0/73 (0) | 1/368 (0.3) |
|  | 2 | 0/66 (0) | 0/74 (0) | 1/79 (1·3) | 0/76 (0) | 0/73 (0) | 1/368 (0.3) |
|  | 3 | 58/66 (87.9) | 70/74 (94.6) | 68/79 (86.1) | 59/76 (77.6) | 57/73 (77.0) | 312/368 (84.1) |
|  | 0 | 7/66 (10.6) | 8/74 (10.8) | 13/79 (16.5) | 29/76 (38.2) | 24/73 (32.9) | 81/368 (22.0) |
| PMC 3 | 1 | 0/66 (0) | 0/74 (0) | 0/79 (0) | 0/76(0) | 0/73(0) | 0/368 (0) |
|  | 2 | 0/66 (0) | 0/74 (0) | 0/79 (0) | 0/76(0) | 0/74(0) | 0/368 (0) |
|  | 3 | 59/66 (89·.) | 66/74 (89.2) | 66/79 (83.5) | 47/76(61.8) | 49/73(67.2) | 287/368 (78.0) |
