# Supplementary material for: Adherence to community versus facility-based delivery of monthly malaria chemoprevention with dihydroartemisinin-piperaquine for the post-discharge management of severe anemia in Malawian children: A cluster randomized trial
Source: PLoS One. 2021 Sep 10;16(9):e0255769. doi: 10.1371/journal.pone.0255769 (PMC8432777; doi:10.1371/journal.pone.0255769)
Supplement: S3 File — (DOCX) [file pone.0255769.s004.docx]

**S 3_Table 5. The distribution of the timing of each dose of PMC per course administered in each trial arm**

| **Course** | | **Com+ No SMS**  **n/N (%)** | **Com+SMS n/N (%)** | **Com+HSA n/N (%)** | **Fac+ No SMS**  **n/N (%)** | **Fac+SMS n/N (%)** | **Overall** |
| --- | --- | --- | --- | --- | --- | --- | --- |
|  | 1^st^ dose | 53/68 (77.9) | 63/74 (85.1) | 69/79 (87.3) | 66/76 (86.8) | 64/74 (86.5) | 310 (83.6) |
| PMC 1 | 2^nd^ dose | 53/68 (77.9) | 63/74 (85.1) | 68/79 (86.1) | 66/76 (86.8) | 64/74 (86.5) |  |
|  | 3^rd^ dose | 52/68 (76.5) | 63/74 (85.1) | 68/79 (86.1) | 65/76 (85.5) | 62/74 (83.8) |  |
|  | 1^st^ dose | 59/66 (89.4) | 70/74 (94.6) | 69/79 (87.3) | 59/76 (77.6) | 57/73 (77.0) | 312 (84.1) |
| PMC 2 | 2^nd^ dose | 58/66 (87.9) | 70/74 (94.6) | 69/79 (87.3) | 59/76 (77.6) | 57/73 (77.0) |  |
|  | 3^rd^ dose | 58/66 (87.9) | 70/74 (94.6) | 68/79 (86.1) | 59/76 (77.6) | 57/73 (77.0) |  |
|  | 1^st^ dose | 59/66 (89.4) | 66/74 (89.2) | 66/79 (83.5) | 47/76 (61.8) | 49/73 (67.1) | 287 (78.0) |
| PMC 3 | 2^nd^ dose | 59/66 (89.4) | 66/74 (89.2) | 66/79 (83.5) | 47/76 (61.8) | 49/73 (67.1) |  |
|  | 3^hs^ dose | 59/66 (89.4) | 66/74 (89.2) | 66/79 (83.5) | 47/76 (61.8) | 49.73 (67.1) |  |
