# Supplementary material for: Adherence to community versus facility-based delivery of monthly malaria chemoprevention with dihydroartemisinin-piperaquine for the post-discharge management of severe anemia in Malawian children: A cluster randomized trial
Source: PLoS One. 2021 Sep 10;16(9):e0255769. doi: 10.1371/journal.pone.0255769 (PMC8432777; doi:10.1371/journal.pone.0255769)
Supplement: S4 File — (PDF) [file pone.0255769.s005.pdf]

Gondwe et al. BMC Pediatrics (2018) 18:238
https://doi.org/10.1186/s12887-018-1199-3

BMC Pediatrics

STUDY PROTOCOL Open Access

Delivery strategies for malaria chemoprevention with monthly dihydroartemisinin-piperaquine for the post-discharge management of severe anaemia in children aged less than 5 years old in Malawi: a protocol for a cluster randomized trial

Thandile Gondwe[1,2*], Bjarne Robberstad[2], Mavuto Mukaka[3,4], Siri Lange[5,6], Bjørn Blomberg[2,7,8] and Kamija Phiri[1]

Abstract

Background: Children initially hospitalized with severe anaemia in Africa are at high risk of readmission or death within 6 months after discharge. No intervention strategy specifically protects children during the post-discharge period. Recent evidence from Malawi shows that 3 months of post-discharge malaria chemoprevention (PMC) with monthly treatment with artemether-lumefantrine in children with severe malarial anaemia prevented 31% of deaths and readmissions. While a confirmatory multi-centre trial for PMC with dihydroartemisinin-piperaquine is on going in Kenya and Uganda, there is a need to design and evaluate an effective delivery strategy for this promising intervention.

Methods: This is a cluster-randomized trial with 5 arms, each representing a unique PMC delivery strategy. Convalescent children aged less than 5 years and weighing more than 5 kg admitted with severe anaemia and clinically stable are included. All eligible children will receive dihydroartemisinin-piperaquine at 2, 6 and 10 weeks after discharge either: 1) in the community without an SMS reminder; 2) in the community with an SMS reminder; 3) in the community with a community health worker reminder; 4) at the hospital with an SMS reminder; or 5) at the hospital without an SMS reminder. For community-based strategies (1, 2 and 3), mothers will be given all the PMC doses at the time of discharge while for hospital-based strategies (4 and 5) mothers will be required to visit the hospital each month. Each arm will consist of 25 clusters with an average of 3 children per cluster giving approximately 75 children and will be followed up for 15 weeks. The primary outcome measure is uptake of complete courses of PMC drugs.

(Continued on next page)

* Correspondence: thandile_nkosi@yahoo.com
[1]College of Medicine, University of Malawi, Private Bag, 360 Blantyre, Malawi
[2]Centre for International Health, Department of Global Public Health and Primary Care, University of Bergen, P.O. Box 7804, 5020 Bergen, Norway
Full list of author information is available at the end of the article

© The Author(s). 2018 **Open Access** This article is distributed under the terms of the Creative Commons Attribution 4.0 International License (http://creativecommons.org/licenses/by/4.0/), which permits unrestricted use, distribution, and reproduction in any medium, provided you give appropriate credit to the original author(s) and the source, provide a link to the Creative Commons license, and indicate if changes were made. The Creative Commons Public Domain Dedication waiver (http://creativecommons.org/publicdomain/zero/1.0/) applies to the data made available in this article, unless otherwise stated.

(Continued from previous page)

Discussion: The proposed study will help to identify the most effective, cost-effective, acceptable and feasible strategy for delivering malaria chemoprevention for post-discharge management of severe anaemia in under-five children in the Malawian context. This information is important for policy decision in the quest for new strategies for malaria control in children in similar contexts.

Trial registration: ClinicalTrials.gov: NCT02721420. Protocol registered on 29 March 2016.The study was not retrospectively registered but there was a delay between date of submission and the date it first became available on the registry.

Keywords: Children, Severe anaemia, Post-discharge malaria chemoprevention, Malaria, Dihydroartemisinin-piperaquine, Cluster randomised trial

Background

Severe anaemia is a reduction in haemoglobin (Hb) concentration below 5 g/dL or haematocrit below 15%. Globally, 43% of children have anaemia and in Africa 3.6% have severe anaemia [1]. Severe anaemia is a leading cause of hospital admissions contributing substantially to paediatric mortality in Africa. Hospitalized children with severe anaemia are particularly at risk within the first 3 months post-discharge, mostly due to a combination of environmental, behavioural, nutritional and genetic risk factors [2–6]. A case-control study in Malawian children indicated that children aged less than 5 years admitted with severe anaemia were not only at high risk of dying during the acute phase in-hospital but also for several months after discharge from the hospital. 17.2% of children with severe anaemia experienced an all cause mortality compared to 2% of controls without severe anaemia [7].

By 18 months post discharge, 10.2% of children with severe anaemia were re-admitted with rebound severe anaemia and 12.6% had died, which is nine times higher than the mortality in community-based, age matched children with mild anaemia. High rates of post-discharge morbidity and mortality have also been reported in western Kenya and Uganda, where 36.5% of children aged less than 5 years admitted with severe anaemia died after 18 months of follow up [3, 7].

Previous observational studies in western Kenya and a recent intervention study in a high transmission areas in Malawi showed that malaria in the post-discharge period is an important contributor responsible for a slow haematological recovery, rebound severe anaemia and morbidity [3, 5, 7]. Many children in these areas experience episodes of new or recrudescent malaria infections after discharge, which negates the initial rise in haemoglobin (Hb) achieved by blood transfusion in hospital [3, 8]. Haematological recovery from malaria-associated anaemia is known to take at least 6 weeks. This period may be prolonged in those with persistent or new malaria infections due to on-going red cell destruction and red blood cell production failure [9].

Standard treatment guidelines for severe anaemia in many countries in sub-Saharan Africa consists of a blood transfusion combined with presumptive intravenous anti-malarial treatment (quinine or artesunate) plus antibiotics if bacterial infections are suspected. Once children have stabilized and can be switched to oral treatment, they receive a 3-day treatment course with artemisinin-based combination therapy (ACT), usually artemether-lumefantrine (AL). Children are often discharged with a short course of iron and folate, typically with no scheduled follow-up [8]. Creating a prophylactic time-window post-transfusion, is suggested to allow time for the bone marrow to recover, resulting in a more sustained haematological recovery post-discharge. Data from a previous study in Malawi show that this process takes 2–3 months in children with severe anaemia [3, 7]. Recently, the use of Intermittent Preventive Treatment (IPT) in children with severe anaemia during the rainy season reduced clinical attacks of malaria by more than 80% in areas with highly seasonal transmission [10]. PMC is a version of IPT designed to clear existing infections and provide prolonged prophylaxis against new infections.

A study in Malawi showed that provision of 3 months of chemoprevention with 3 full treatment courses of artemether-lumefantrine (AL), given in-hospital for initial malaria episode and at 1 and 2 months post-discharge, prevented 41% of deaths or readmissions due to severe anaemia or severe malaria during a 6 months follow-up period [2, 7, 11, 12]. These results are consistent with studies of children with severe anaemia in Gambia, who received chemoprevention given as monthly intermittent preventive therapy with SP or as weekly prophylaxis with pyrimethamine-dapsone targeted during the malaria transmission season [12]. For these children, the rate of clinical malaria was halved and all-cause hospital readmission was reduced by 78% in one trial, and recurrence of severe anaemia was reduced by 78% in the other [12–14]. These data indicate that IPT in the post-discharge period may potentially provide substantial health benefits [13].

Rationale

In the past two decades, most research on severe anaemia and severe malaria have focused on reducing in-hospital mortality. A major and potentially preventable component of the disease burden occurs after discharge from hospital and a proactive approach using PMC could offer substantial public health gains [11], and is a priority area for research. There is, however, no specific delivery strategy that has been scaled up to address this high-risk post-discharge period. Despite being a relatively simple intervention, the implementation of PMC in African settings may face challenges due to weak healthcare systems that are unique in different contexts. PMC requires appropriately designed delivery strategies because treatment must be administered for 3 days at different pre-specified intervals post-discharge.

Delivery of PMC to the target population will require new systems to be established that are sustainable and cost-effective. In contrast to IPT strategies in infants (IPTi) and pregnant women (IPTp), which are delivered through the expanded programme on immunization (EPI) and antenatal clinics, the delivery strategies for PMC are not yet in place. Some evidence shows that delivery of IPT to children (IPTc) through community health workers is feasible and well accepted in rural West Africa [12]. In Malawi, the health system also include village health volunteers (VHVs) and Health Surveillance Assistants (HSAs) who could deliver PMC or schedule post-discharge visits to clinics or hospital outpatient departments for subsequent PMC doses [14]. Since PMC targets a very high-risk group of hospitalized children who already have contact with the health-care system, the point of entry is already established [2, 12]. This trial is part of a larger project under the PMC consortium with 5 main activities in Malawi, Kenya and Uganda that aim to address the gaps in knowledge on whether PMC should be recommended as a strategy for the post-discharge management of children with severe anaemia to the WHO. The PMC trial in Malawi specifically aims to identify the most effective, acceptable and cost-effective strategy for delivering PMC and if use of short message service has additional benefits.

Study aims and objectives

The primary objective of the trial is to determine the optimum PMC delivery strategy by comparing community-based versus health facility-based strategies in order to inform policy decision. Specifically we aim to compare PMC uptake (adherence) levels and safety between community-based versus health facility-based strategies. To determine and compare health system and family/household costs of delivering and receiving PMC using the alternative proposed strategies. To assess the feasibility and acceptability of delivering and receiving PMC in a typical Malawi health system setting. To determine whether SMS or Health Surveillance Assistant (HSA) reminder has additional benefit on PMC uptake (adherence) levels. To estimate the incremental cost-effectiveness and equity impact of the alternative delivery strategies.

Methods

Study design

This is a single-centre open label cluster randomized clinical trial with 5-arms. A cluster represents a village, which is the smallest administrative unit and overseen by a village headman. A total of 1387 villages in the catchment areas of Zomba Central hospital in southern Malawi will be involved in this study.

Trial drug

The study drug used in this trial is Eurartesim® manufactured by Sigma-Tau pharmaceuticals. This is a GMP certified co-formulated artemisin-based antimalarial combination, which contains 20 mg Dihydro-Artemesinin and 160 mg Piperaquine. It is administered according to body weight 2 (PMC-1), 6 (PMC-2) and 10 weeks (PMC-3) after discharge from hospital, each course for three consecutive days.

Study participants

Children aged 4 to 59 months admitted to Zomba central hospital with a diagnosis of severe anaemia either by haemoglobin of < 5 g/dl or clinically assessed as severely anaemic but clinically stable and have received all treatments according to the hospital standard of care are screened for inclusion in the trial at the time of discharge from hospital. Caretakers of children who meet the eligibility criteria are given general information about the trial and those who are interested are required to give written consent for the child to participate into the study.

Interventions

All children receive DHP and hence there are no placebo arms in this trial. However, children are randomized to receive PMC as follows:

Community-based arms

Arm 1

PMC drugs given at discharge without SMS reminder: The guardian receives all drugs for PMC-1, PMC-2 and PMC-3. They are instructed on how and when to give these drugs to the children when being discharged from hospital. The dates for each course are documented in the child's health book.

Arm 2

PMC drugs given at discharge with SMS reminders: The guardian receives all drugs for PMC-1, PMC-2 and

PMC-3 and is instructed on how and when to give these drugs. Additionally they are reminded via SMS to give the drugs to the child one day before each treatment course is due.

Arm 3

PMC drugs at discharge with Health Surveillance Assistant (HSA) reminders: The guardian receives all drugs for PMC-1, PMC-2 and PMC-3 and is instructed on how and when to give these drugs. Additionally, HSAs which are part of existing networks of community-based volunteers taking part in village health committees are reminded via SMS to go and remind the guardian to give the drugs to the child one day before each treatment course is due.

Hospital/facility based arms

Arm 4

PMC drugs collected from the hospital: At discharge, the guardian is instructed to return to the outpatient department (OPD) of the hospital each month to collect drugs for PMC-1, PMC-2 and PMC-3. They do not receive any form of reminder other than what has been documented in the child's health card.

Arm 5

PMC drugs at hospital with SMS reminders: At discharge, the guardian is requested to return to the OPD to collect drugs for PMC-1, PMC-2 and PMC-3. Additionally they will be reminded via SMS to come to the clinic to collect drugs one day before each treatment course is due.

Recruitment and follow up

Figure 1 shows the study flowchart from the standard protocol items: recommendations for interventional trials (SPIRIT). Every morning, the study staff at Zomba Central Hospital goes through all newly admitted children's files in order to identify and pre-screen children who meet the study criteria. During this acute phase of

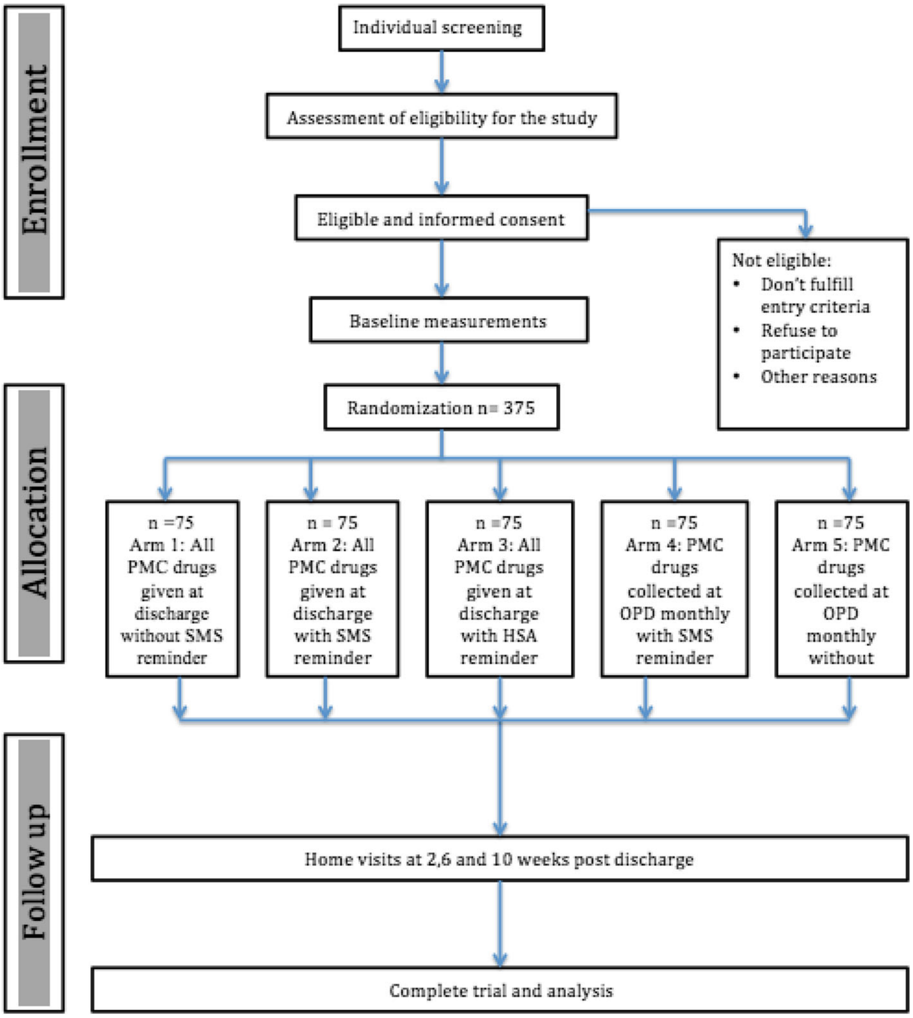

Fig. 1 Study flowchart adapted from the standard protocol items: recommendations for interventional trials (SPIRIT)

illness, pre-study screening involves confirming the study criteria and provision of routine standard of care treatment. No study specific information or samples are collected in this point of time. The role of the study team during pre-study screening is to review the diagnosis and ensure that the potential study participants get the standard quality of care for severe malarial anaemia.

Each pre-screened subject is assigned a pre-screening number in sequential order regardless of whether they fulfil the pre-screening eligibility criteria. After the child has recovered sufficiently, the caretaker is approached for further screening for eligibility. Caretakers of children who fulfil the eligibility criteria and who agree to participate in the research provide written informed consent before enrolling into the study. The study participant's demographic data, relevant clinical information, including the previous and current medical history, and laboratory information is collected simultaneously. Furthermore, a physical and clinical examination is performed and captured on the enrolment case report forms (CRFs).

Randomization and allocation methods

The unit of randomization is villages within the catchment area of Zomba central hospital. 400 out of the 1387 villages were randomly selected and randomized to one of the study arms, where each arm represents a different implementation alternative. When a child is enrolled and all study procedures have been done, the study data officer enters the name of the village where the child resides into a pre-programed database and this automatically generates the study arm and study identification number. The participants and the study staff are aware of the study arm assigned. However, the study statistician who will perform the final analyses will be blinded to the allocated treatment arm.

Follow up procedures

Table 1 summarises the recruitment and follow up procedures for the study. Depending on the trial arm to which the child is allocated, the caretaker is either scheduled to return to OPD clinic for PMC drugs at two, six and ten weeks (facility based strategies), or be given all the courses of drugs at discharge to administer them at home also at two, six and ten weeks (community based strategies). This information is documented in the child's health book. All participants, irrespective of arm, are visited at home shortly after the two, six and ten scheduled PMC treatments to assess DHP uptake. These home visits are for adherence assessment, vital registration and health economic assessments only, and not for clinical assessment. The PMC follow up period

Table 1 Recruitment and follow-up procedures

	Enrolment	Allocation	Follow up			
TIMEPOINT	*-t₌ 2 wks.*	0	*t = 2 wks.*	*t = 6 wks.*	*t = 10 wks.*	*t = 15 wks./study end*
ENROLMENT						
Eligibility screen	X					
Informed consent	X					
Baseline measurements	X					
Allocation		X				
INTERVENTIONS:						
Arm 1: All PMC drugs given at discharge without monthly SMS reminder			X	X	X	
Arm2: all PMC drugs given at discharge with monthly SMS reminder			X	X	X	
Arm 3: All PMC drugs given at discharge with HAS reminder.			X	X	X	
Arm 4: PMC drugs collected monthly at the OPD with SMS reminder.			X	X	X	
Arm 5: PMC drugs collected monthly at the OPD without SMS reminder			X	X	X	
ASSESSMENTS:						
Physical examination	X					X
Blood samples	X					X
Adverse event assessment	X	X	X	X	X	X
OUTCOMES						
Adherence						X
Clinical safety			X	X	X	X
Cost-effectiveness						X
Acceptability and feasibility						X

ends at 15 weeks after enrolment, which is four weeks after the third scheduled PMC course. Participants are then requested to come to the clinic 15 weeks after enrolment for an end of study assessment. Transport is reimbursed for the study end visit. In addition, subject's parent or guardian are instructed to return his/her child to the study clinic for evaluation free of charge at any time if their condition warrants medical attention during the 15 weeks follow-up period after discharge.

Data collection and outcome measures

Adherence outcome measures

The primary outcome is the level of uptake of PMC drugs which will be assessed by unannounced home visits after each course whereby pill counts will be done. Hundred percent uptake of PMC drugs will be defined as administration of all 3-day treatment courses (i.e. 9 doses), given at 2, 6 and 10 weeks after discharge from hospital. Sixty percent of PMC drugs are defined as administration of 6 or more (but less than 9) of the daily dosages out of the total of 9.30% of PMC drugs will be defined as administration of 3 or more (but less than 6) of the daily dosages out of the total of 9. Less than 30% of PMC drugs is defined as administration of less than 3 of the daily dosages out of the total of 9, given 2, 6 and 10 weeks after discharge.

Clinical outcomes

Study instruments include structured forms for clinical history, physical assessment, laboratory evaluations and clinical information collected on history of current and previous illnesses and hospital admissions is collected in addition to hospitalizations and mortality. For children who are hospitalized during the follow-up period, data is collected on the length of hospitalization, diagnosis, treatments provided, laboratory results, and participant outcomes. Physical examination and laboratory tests include: height, weight, mid-upper arm circumference, malaria rapid test results, blood slide results, parasitaemia level, and haemoglobin concentration. This will be used to assess all-cause mortality, incidence rate of all-cause hospital readmissions, incidence rate of readmissions due to severe anaemia (Hb < 5 g/dL) or severe malaria defined by the administration of parenteral Artesunate or Quinine, incidence rate of non-severe all-cause sick-child clinic visits, specifically incidence rate of clinic visit due to RDT/microscopy confirmed non-severe malaria during the study period.

Cost and economic evaluation outcomes

Information about expenses, resource and time use with open-ended fields for unanticipated findings and place for comments will be collected. Demographic information on children is collected including age, sex, while religion, village of residence and socioeconomic features of their household is also recorded as well as cost information for the economic evaluation. This will assess health provider's cost of delivering the PMC services, caretakers' cost of receiving the PMC services, incremental cost-effectiveness ratio (ICER) of PMC delivery strategies and equity impact by socioeconomic status of PMC delivery strategies on main and secondary outcomes.

Acceptability and feasibility outcomes

In addition there will be interview guides for in-depth interviews and focus group discussions with a selection of caretakers and health workers who have been involved in the PMC trial. This information will be used to assess the acceptability of PMC, adaptations to health workers' working practices required to implement PMC, perceptions of implementing PMC through different delivery strategies, and recommendations on effective implementation.

Sample size

The sample size calculation has been adjusted for the design effect using the coefficient of variation method in order to account for the Intra-cluster Correlations (ICC) [15, 16]. We assumed that the cluster sizes would be uniformly distributed between 2 and 4 children [2, 4]. This gives a mean cluster size of 3 children per village per year and a standard deviation of cluster sizes of 0.58. Hence the coefficient of variation of cluster sizes (CV) is 0.58/3 = 0.19 and $CV^2 = 0.036$. Assuming an intra-cluster correlation coefficient (ICC) of 0.1 and allowing for 10% loss-to-follow-up, a sample size of 25 clusters (villages) of an average of 3 children per village (75 children per arm, $N = 375$ overall (for 125 clusters for the 5 arms)) has 80% power to detect a 25% absolute increase in uptake from an estimated 50% in the OPD and delivery at home groups to 75% in the arms supported by SMS reminders ($\alpha = 0.05$). The ICC of 0.1 is slightly more conservative than the ICC in a previous trial of delivery approaches for IPTc in the Gambia.

Data management and statistical analysis

Data is collected and recorded by one of the trained study staff at the point of contact. Data is entered directly into computer tablets that will be pre-programmed and uploaded with electronic case report forms (eCRFs). The eCRFs have crosschecks for verification, validation and comply with Good Clinical Practices (GCP). These gadgets are connected to a desktop allowing data to be directly uploaded into a database.

The percentage of children receiving PMC according to schedule in each arm will be obtained and compared between arms using relative risks (RR) and the 95% CI for the RR will be reported. The estimates of the RR will be adjusted for prognostic factors and potential confounding factors at baseline using log binomial or Poisson regression

with adjustment for cluster effects. Using Cox regression, hazard ratios will be calculated for morbidity endpoints such as incidence of severe anaemia, severe malaria and all cause hospital re-admissions, for repeated events with robust standard error estimation methods to account for correlation between episodes within children. Incidence rates per child-year and absolute rate reductions will also be calculated.

Discussion

This protocol describes a study in which we will determine the optimum PMC delivery mechanism in a Malawian context by comparing community versus health facility based strategies. Presently in African health systems and particularly in Malawi, post-discharge health management systems are not in place, and there is need to assess a number of relevant strategies for effective delivery of PMC in a largely rural community that would typically benefit from this intervention.

We postulate that this post-discharge care in the form of PMC may be delivered either in the community or at a health facility. The most likely scenario where the health care provider is least involved would be where mothers are given all the PMC drugs on discharge from hospital and allowed to administer the drugs on her own to the child (Arm 1). In recent years there has been successful disease control programs operationalizing more decentralized drug delivery programs in the community in low-income countries, making it a viable option for PMC. However as we postulate that the mother could forget to administer the PMC, we would like to test two different reminder systems. The first is through the use of SMS technology, which has been shown to be user-friendly and acceptable from our own pilot work (unpublished pilot study) and other current programs in Malawi (Arm 2 and 5).

An alternate reminder system is the use of Health Surveillance Assistants (HSA). HSA are Ministry of Health (MoH) employees who are responsible for basic health promotion activities in the community. Ideally MoH strives to have one HSA for every 1000 people, but in reality they often cater for much larger populations and are usually over-burdened with many disease control programs. In some areas in rural Malawi there are Village Health committees, which are made up of voluntary members of the community. They work hand in hand with the HSA. We postulate that HSAs and where available village health volunteers (VHVs) are an option for reminding mothers to give PMC to their child (Arm 3).

Another option for delivery of PMC is to request the mother or caretaker to return to the health care facility to collect the drugs for each treatment course (Arms 4 and 5). This is a plausible strategy as it is consistent with the management of chronic illness such as TB and HIV where drugs are routinely collected from a health facility by the patients. This would be an alternative strategy as part of EPI or management of other chronic illnesses e.g. chest follow up clinic.

Abbreviations

ACTs: Artemisin based combination therapies; AL: Artemether-Lumefantrine; CRF: Case Report Form; DHP: Dihydro-Artemesinin-Piperaquine; EC: Ethics Committee; EPI: Expanded programme for immunizations; GCP: Good Clinical Practice; Hb: Haemoglobin concentration; HSA: Health surveillance assistant; ICC: Intra-Cluster Correlation; IPT: Intermittent Preventive Therapy; PMC: Post discharge malaria chemoprevention; SMS: Short message service; SP: Sulfadoxine-Pyrimethamine; WHO: World Health Organization

Author contributions

The authors include all professionals/co-investigators that have participated in the trial for a minimum of one year and these include BR, KP, MM, SL, BB and TG. BR, KP, BB and TG contributed to the design of the trial and clinical methods. BR contributed the economic evaluation methods and analysis plan. MM contributed to the randomization procedures, statistical analysis plan and other areas of methodology. SL contributed the qualitative methods and analysis. TG coordinated the implementation of the trial. TG wrote the first draft of the manuscript and all authors have approved the final version.

Funding

The study was funded by the Research Council of Norway through the Global Health and Vaccination Programme (GLOBVAC), project number 234487. GLOBVAC is part of the EDCTP2 programme supported by the European Union. The Council had no role in the design of the study, in the collection, analysis and interpretation of the data, or in writing and Preparation of the manuscript.

Ethics approval and consent to participate

The protocol and related documents (informed consent and participant information sheets) were submitted for review to the Institutional Review Boards (IRB) and Research Ethics Committees at the Malawi College of Medicine and the Regional Ethics Committee of Norway. The study was approved by the College Of Medicine research ethics committee (COMREC), approval number P.02/15/1679, and the Regional Ethics Committee of Norway, approval number 2015/537/REK vest. Written consent was obtained from legal guardians of the study participants prior to enrolment.

Consent for publication

Not applicable.

Competing interests

The authors declare that they have no competing interests.

Publisher's Note

Springer Nature remains neutral with regard to jurisdictional claims in published maps and institutional affiliations.

Author details

[1]College of Medicine, University of Malawi, Private Bag, 360 Blantyre, Malawi. [2]Centre for International Health, Department of Global Public Health and Primary Care, University of Bergen, P.O. Box 7804, 5020 Bergen, Norway. [3]Mahidol-Oxford Tropical Medicine Research Unit, Mahidol University, Bangkok, Thailand. [4]Centre for Tropical Medicine, Nuffield Department of Medicine, University of Oxford, Oxford, UK. [5]Chr. Michelsen Institute, Jekteviksbakken 31, 5006 Bergen, Norway. [6]Department of Health Promotion and Development, University of Bergen, Christiesgt. 13, 5020 Bergen, Norway. [7]Department of Clinical Science, University of Bergen, Bergen, Norway. [8]National Centre for Tropical Infectious Diseases, Department of Medicine, Haukeland University Hospital, Bergen, Norway.

Received: 24 May 2018 Accepted: 27 June 2018
Published online: 20 July 2018

References

1. WHO. The global prevalence of anaemia in, vol. 2011. Geneva: World Health Organization; 2015.
2. Bojang KA, Milligan PJM, Conway DJ, Sisay-Joof F, Jallow M, Nwakanma DC, et al. Prevention of the recurrence of Anaemia in Gambian children following discharge from hospital. PLoS One. 2010;5(6):e11227.
3. Phiri KS, Calis JCJ, Faragher B, Nkhoma E, Ng'oma K, Mangochi B, et al. Long term outcome of severe Anaemia in Malawian children. PLoS One. 2008; 3(8):e2903.
4. Lackritz EM, Hightower AW, Zucker JR, Ruebush TK 2nd, Onudi CO, Steketee RW, et al. Longitudinal evaluation of severely anemic children in Kenya: the effect of transfusion on mortality and hematologic recovery. AIDS. 1997; 11(12):1487–94.
5. Calis JCJ, Phiri KS, Faragher EB, Brabin BJ, Bates I, Cuevas LE, et al. Severe Anemia in Malawian children. N Engl J Med. 2008;358(9):888–99.
6. Bojang KA, Van Hensbroek MB, Palmer A, Banya WA, Jaffar S, Greenwood BM. Predictors of mortality in Gambian children with severe malaria anaemia. Ann Trop Paediatr. 1997;17
7. Lwin KM, Phyo AP, Tarning J, Hanpithakpong W, Ashley EA, Lee SJ, et al. Randomized, double-blind, placebo-controlled trial of monthly versus bimonthly Dihydroartemisinin-Piperaquine chemoprevention in adults at high risk of malaria. Antimicrob Agents Chemother. 2012;56(3):1571–7.
8. Price RN, Simpson JA, Nosten F, Luxemburger C, Hkirjaroen L, ter Kuile F, et al. Factors contributing to anemia after uncomplicated falciparum malaria. The American Journal of Tropical Medicine and Hygiene. 2001;65(5):614–22.
9. Bojang KA, Palmer A, Boele van Hensbroek M, Banya WA, Greenwood BM. Management of severe malarial anaemia in Gambian children. Trans R Soc Trop Med Hyg. 1997;91(5):557–61.
10. WHO World Malaria Report 2016. Geneva: World Health Organization; 2016.
11. Phiri K, Esan M, van Hensbroek MB, Khairallah C, Faragher B, ter Kuile FO. Intermittent preventive therapy for malaria with monthly artemether–lumefantrine for the post-discharge management of severe anaemia in children aged 4–59 months in southern Malawi: a multicentre, randomised, placebo-controlled trial. Lancet Infect Dis 2012;12(3):191–200.
12. Bojang KA, Akor F, Conteh L, Webb E, Bittaye O, Conway DJ, et al. Two strategies for the delivery of IPTc in an area of seasonal malaria transmission in the Gambia: a randomised controlled trial. PLoS Med. 2011;8(2):e1000409.
13. White NJ. Intermittent presumptive treatment for malaria. PLoS Med. 2005;2(1):e3.
14. Steketee RW, Slutsker L. Targeting of intermittent preventive treatment for malaria. Lancet Infect Dis12(3):168–9.
15. Manatunga AK, Hudgens MG, Chen S. Sample size estimation in cluster randomized studies with varying cluster size. Biom J. 2001;43(1):75–86.
16. Eldridge SM, Ashby D, Kerry S. Sample size for cluster randomized trials: effect of coefficient of variation of cluster size and analysis method. Int J Epidemiol. 2006;35(5):1292–300.

Ready to submit your research? Choose BMC and benefit from:

- fast, convenient online submission
- thorough peer review by experienced researchers in your field
- rapid publication on acceptance
- support for research data, including large and complex data types
- gold Open Access which fosters wider collaboration and increased citations
- maximum visibility for your research: over 100M website views per year

At BMC, research is always in progress.

Learn more biomedcentral.com/submissions
