# Supplementary material for: Adherence to community versus facility-based delivery of monthly malaria chemoprevention with dihydroartemisinin-piperaquine for the post-discharge management of severe anemia in Malawian children: A cluster randomized trial
Source: PLoS One. 2021 Sep 10;16(9):e0255769. doi: 10.1371/journal.pone.0255769 (PMC8432777; doi:10.1371/journal.pone.0255769)
Supplement: S5 File — (PDF) [file pone.0255769.s006.pdf]

Malaria Chemoprevention with monthly treatment with dihydroartemisinin-piperaquine for the post-discharge management of severe anaemia in children aged less than 5 years in Malawi: A 3-year, single site, parallel-group, five-arm cluster randomised trial of community versus health facility-based delivery mechanisms with or without mobile phone sms reminder

Short Title: Post-discharge Malaria Chemoprevention Delivery (PMC) study

Trial Identifiers:

ClinicalsTrials.gov: NCT02721420	The College of Medicine Research and Ethics Committee (COMREC) [P.02/15/1679]	Regional Committees for Medical and Health Research Ethics (REK vest) (2015/537]

Chief Investigator:

- **Dr Kamija Phiri**, College of Medicine, University of Malawi, Private bag 360, Blantyre. Mobile: +265 999957048, Email: kamijaphiri@gmail.com

Principal Investigator:

- **Dr Thandile Nkosi-Gondwe**, College of Medicine, Private Bag 360, Blantyre, Malawi. Mobile: +265 995508830, Email: thandile_nkosi@yahoo.com

Co-Investigators***:*** see page Co-Investigators page 8

Trial Statistician:

- **Dr Mavuto Mukaka**, Johns Hopkins University, Bloomberg School of Public Health, Department of International Health, 615 N. Wolfe Street, Room W5024, Baltimore MD 21205, USA. Email: mmukaka@gmail.com

Funder: The Research Council of Norway, Global Health and Vaccination Research (GLOBVAC). Project id: 234487.

Sponsor: College of Medicine (CoM), University of Malawi, Private Bag 360, Blantyre, Malawi

Amendment History			
Date	Protocol Version	Details of Changes	Signature Chief Investigator
31 Jan 2015	1 .1	Original	
23 May 2017	1. 2		

1 TABLE OF CONTENTS

2 TITLE OF RESEARCH PROTOCOL 8
3 INVESTIGATORS AND INSTITUTIONS 8
3.1 INVESTIGATORS 8
3.1.1 Chief Investigator 8
3.1.2 Principal Investigator 8
3.1.3 Trial Statistician 8
3.1.4 Co-Investigators 8
3.2 NON-ENGAGED COLLABORATORS 9
3.3 INSTITUTIONS 9
4 Protocol Summaries 10
4.1 Trial Registration Data 10
4.2 Narrative Protocol Summary 14
6 BACKGROUND 19
6.1 High risk of severe anaemia rebound after discharge: 19
6.2 Malaria as a cause of post-discharge severe anaemia and mortality 20
6.3 Treatment strategies for prevention of malaria and haematological recovery post-discharge: 20
6.4 Proposed PMC delivery mechanisms trial 20
6.5 Rationale for choice of methods 20
7 STUDY OBJECTIVE 21
7.1 Primary objective 21
7.2 Specific objectives 21
7.3 OUTCOMES 21
7.3.1 Primary outcome 21
7.3.2 Secondary outcomes 21
8 DESIGN AND METHODOLOGY 22
8.1 Trial Design 22
8.1.1 Rationale for the trial arms 23
8.1.2 Sub-studies 24
8.2 STUDY SITE 24
8.3 SAMPLE SIZE 24
8.4 DATA COLLECTION TOOLS 25
8.5 VARIABLES 25

8.6 **TRIAL MEDICATION AND INTERVENTIONS** 25
8.6.1 Study Medications 25
8.6.2 Drug administration 27
8.6.3 Information on how to give study drugs at home 28
8.6.4 Procedures for Drug handling & Accountability 29
8.6.5 Prohibited medications 30
8.7 **SELECTION AND WITHDRAWAL OF STUDY PARTICIPANTS** 30
8.7.1 Inclusion Criteria 30
8.7.2 Eligibility criteria for pre-study screening 30
8.7.3 Screening and Consent 30
8.7.4 Enrolment 31
8.7.5 Unscheduled visits (passive follow-up) 33
8.7.6 Adherence to study intervention protocol and strategies for retention 33
8.7.7 Prior and concomitant therapy 34
8.8 **PARTICIPANT WITHDRAWAL** 35
8.9 **OUTCOMES** 35
8.9.1 Primary Outcome 35
8.9.2 Secondary Outcomes 36
8.10 **EXPECTED DURATION OF TRIAL** 36
8.11 **PROCEDURES FOR RECORDING AND REPORTING ADVERSE EVENTS** 37
8.11.1 Reporting to sponsor 37
8.11.2 Treatment Stopping Rules 37
8.12 **PARTICIPANTS TIMELINE** 38
8.12.1 Overview Study Phases 38
8.12.2 Pre-screening 38
8.12.3 Screening interview and consent & Enrolment 38
8.12.4 1st, 2nd and 3rd PMC treatment schedules 40
8.12.5 Study end visit 40
8.12.6 Unscheduled visits (passive follow-up) 40
9 SUB-STUDIES 41
9.1 **ECONOMIC EVALUATION** 41
9.1.1 Patient intervention costs 42
9.1.2 Patient disease costs 42
9.1.3 Provider intervention costs 42
9.1.4 Provider disease costs 42
9.2 **EQUITY IMPACT ANALYSIS** 43

9.3 **QUALITATIVE STUDY ON ACCEPTABILITY AND PERCEPTIONS ON FEASIBILITY** 43
9.3.1 Health providers 44
9.3.2 Carers of children: 44
10 Programme Management 45
10.1 **TRIAL AUTHORISATION** 45
10.2 **TRIAL STEERING COMMITTEE** 45
10.3 **TRIAL SPONSORSHIP AND QUALITY ASSURANCE** 45
10.4 **NATIONAL AND INTERNATIONAL COLLABORATION AND NETWORKING** 45
10.4.1 Impact of the project and Key beneficiaries 46
10.5 **DIRECT ACCESS TO SOURCE DATA AND DOCUMENTS** 46
11 Data Collection, Management and Analysis 46
11.1 **DATA COLLECTION** 47
11.2 **DATA ENTRY AND EDITING** 47
11.3 **DATA SHARING** 47
11.4 **DATA STORING AND ARCHIVING** 47
11.5 **QUALITY ASSURANCE** 47
11.5.1 Quality Control of the trial 47
11.5.2 Quality assurance/control of laboratory tests 48
11.6 **DATA HANDLING** 48
11.7 **STATISTICS AND DATA ANALYSES** 48
11.7.1 Sample Size and randomization 48
11.7.2 Randomisation procedure 48
11.7.3 Protecting against bias 49
11.7.4 Data Analysis 49
12 Result dissemination and publication policy 49
12.1 **AUTHORSHIP AND PUBLICATIONS** 50
12.2 **INSURANCE / INDEMNITY** 50
12.3 **FINANCIAL ASPECTS** 50
13 Capacity building 50
13.1 **TRAINING, FELLOWSHIPS AND CAPACITY BUILDING** 50
13.2 **AFRICAN LEADERSHIP** 51
14 Ethics & Regulatory Approvals 51
14.1 **DECLARATION OF HELSINKI** 51
14.2 **RESEARCH ETHICS COMMITTEE AND INSTITUTIONAL REVIEW BOARDS** 51
14.3 **INFORMED CONSENT** 51

14.4 **PROTECTION OF PRIVACY AND CONFIDENTIALITY** ... 52
14.4.1 Privacy of individual ... 52
14.4.2 Confidentiality of data ... 52
14.5 **ETHICAL CONSIDERATIONS FOR HUMAN PARTICIPANTS** ... 52
14.5.1 Risks from blood sampling ... 52
14.5.2 Safety of study drug in children ... 52
14.5.3 Methods to minimise risks ... 52
14.5.4 Experience with DP as PMC ... 52
14.5.5 Anticipated benefits to study participants ... 53
14.5.6 Benefit to the community ... 53
14.5.7 Reimbursement of costs ... 54
14.6 **Ancillary and post-trial care** ... 54
14.6.1 Health care during the trial ... 54
14.6.2 Trial insurance ... 54
14.6.3 Post-trial care ... 54
15 REFERENCES ... 54
17 FINANCIAL ASPECTS AND CONFLICT OF INTEREST ... 57
17.1 **Funding of the trial** ... 57
17.2 **Provision of the study drugs** ... 57
18 BUDGET AND BUDGET JUSTIFICATION ... 57
18.1 **Budget** ... Error! Bookmark not defined.
18.2 **Budget Justification** ... 57
20 APPENDICES ... 59
20.1 **APPENDIX I. ROLE INVESTIGATORS AND NON-ENGAGED COLLABORATORS** ... 59
20.1.1 Protocol development: authors' contributions ... 59
20.1.2 Role Investigators ... 59
20.1.3 Role Non-Engaged Collaborators ... 59
20.2 **APPENDIX II. TERMS OF REFERENCE OVERSIGHT COMMITTEES** ... 60
20.2.1 Trial Management Group (TMG) ... 60
20.2.2 Trial Steering Committee (TSC) ... 60
20.2.3 Data Monitoring and Ethics Committee (DMEC) ... 62
20.3 **APPENDIX III. DECLARATION OF HELSINKI** ... 63

Glossary of Terms

ACTs	Artemisin–based combination therapies
AE	Adverse event
AL	Artemether-Lumefantrine
Cmax	maximum plasma concentration
CRF	Case Report Form
DHP	Dihydroartemisinin-Piperaquine
DSMB	Data safety monitoring Board
REC	Research Ethics Committee
EC	Ethics Committee
GCP	Good Clinical Practice
Hb	Haemoglobin
HIV	Human Immunodeficiency Virus
IRB	Institutional Review Board
ITNs	Insecticide Treated Nets
IPTpd	Intermittent Preventive Therapy post-discharge
LLITNs	Long Lasting Insecticide Treated Nets
PCR	Polymerase Chain Reaction
PMC	Post Malaria Chemoprevention
SAE	Serious Adverse Event
SOP	Standard Operating Procedures
SP	Sufadoxine-pyrimethamine
QRS	Time interval between Q-,R- and S- waves on ECG records
QT	Time interval between Q- to T-wave (interval of electrocardiogram)
QTc	QT corrected
TSC	Trial Steering Committee
WHO	World Health Organization

2 TITLE OF RESEARCH PROTOCOL

Malaria Chemoprevention with monthly treatment with dihydroartemisinin-piperaquine for the post-discharge management of severe anaemia in children aged less than 5 years in Malawi: A 3-year, single site, parallel-group, five-arm cluster randomised trial of community versus health facility-based delivery mechanisms with or without mobile phone SMS reminders

Short Title/Acronym: Post-discharge Malaria Chemoprevention Delivery (PMC) study

3 INVESTIGATORS AND INSTITUTIONS

3.1 INVESTIGATORS

3.1.1 Chief Investigator

Dr Kamija S. Phiri, College of Medicine, University of Malawi, P/Bag 360, Blantyre, Malawi. Mobile: +265 999957048, Email: kamijaphiri@gmail.com

3.1.2 Principal Investigator

Dr Thandile Gondwe, College of Medicine, P/Bag 360, Blantyre, Malawi. Mobile: +265 995508830, Email: thandile_nkosi@yahoo.com

3.1.3 Trial Statistician

Dr Mavuto Mukaka, Johns Hopkins University, Bloomberg School of Public Health, Department of International Health, 615 N. Wolfe Street, Room W5024, Baltimore MD 21205, USA. Email: mmukaka@gmail.com

3.1.4 Co-Investigators

Norway	Prof Bjarne Robberstad[2]
	Dr Siri Lange[3]
Uganda	Dr Richard Idro[4]
	Dr Robert Opoka[4]
Kenya	Prof Feiko ter Kuile[5]
	Dr Meghna Desai[5]
Netherlands	Prof Michael Boele van Hensbroek[7]
UK	Prof Azra Ghani[8]
	Dr Mathews Cairns[9]
USA	Prof Chandy John[10]

3.2 NON-ENGAGED COLLABORATORS

Mrs Doreen Ali[11], Deputy Director, National Malaria Control Program, Ministry of Health, Malawi

Dr Martias Joshua[12], Hospital Director, Zomba Central Hospital, Ministry of Health, Zomba, Malawi

3.3 INSTITUTIONS

[1] College of Medicine, University of Malawi, P/Bag 360, Blantyre, Malawi

[2] Centre for International Health, & Department of Global Public Health and Primary Care, University of Bergen, Bergen, Norway

[3] Chr. Michelsen Institute (CMI), Norway

[4] College of Health Sciences, Makerere University, Kampala Uganda

[5] Liverpool School of Tropical Medicine (LSTM), Liverpool, United Kingdom

[6] KEMRI Centre for Global Health Research (CGHR), Kisumu, Kenya

[7] Emma Children's Hospital, Academic Medical Centre, University of Amsterdam, the Netherlands

[8] MRC Centre for Outbreak Analysis & Modelling, Department of Infectious Disease Epidemiology, Imperial College London, London, United Kingdom

[9] London School of Hygiene and Tropical Medicine, university of London, London, United Kingdom

[10] Division of Global Pediatrics, University of Minnesota Medical School, Duluth, MN, USA

[11] National Malaria Control Program, Ministry of Health, Malawi

[12] Zomba Central Hospital, Ministry of Health, Zomba, Malawi

4 Protocol Summaries

4.1 Trial Registration Data

Data Category	Information	
Primary Registry and Trial identifying number	ClinicalTrials.gov: NCT02721420.	
Date of registration in primary registry	[dd mmm yy]	
Secondary identifying numbers	COMREC P.02/15/1679	Norwegian REC ####
Source(s) of monetory or material support	The Research Council of Norway, Global Health and Vaccination Research (GLOBVAC), grant 234487	
Primary Sponsor	College of Medicine, University of Malawi, P/Bag 360, Blantyre Malawi	
Secondary Sponsor	N/A	
Contact for public queries	Dr Kamija Phiri, email: kphiri@medcol.mw	
Contact for Scientific queries	Dr Kamija Phiri, email: kphiri@medcol.mw	
Public Title	Delivery of Malaria Chemoprevention in the post-discharge management of children with severe anaemia in Malawi	
Scientific Title	Malaria Chemoprevention with monthly treatment with dihydroartemisinin-piperaquine for the post-discharge management of severe anaemia in children aged less than 5 years in Malawi: A 3-year, single site, parallel-group, five-arm cluster randomised trial of community versus health facility-based delivery mechanisms with or without mobile phone SMS reminders	
Countries of recruitment	Malawi	
Health Condition(s) or problems studied	Malaria, readmissions after severe anaemia	
Interventions	Dihydroartemisinin-piperaquine (3-day treatment courses, given 2,6 and 10 weeks after discharge):<br>a) at discharge + SMS Reminder	

	b) at discharge + No SMS Reminder<br>c) at discharge + HSA Reminder<br>d) at OPD + SMS Reminder<br>e) at OPD + No SMS Reminder
Study Type	Interventional
	Allocation: cluster randomised; intervention model: parallel assignment; arms: 5; allocation ratio 1:1; Masking: open-label
	Primary purpose: prevention
	Phase-III
Date of enrolment	[24 Mar 16]
Target sample size	375
Recruitment status	Recruiting
Key Inclusion Criteria	1. Haemoglobin <5.0g/dl or PCV <15%, or requirement for blood transfusion for other clinical reasons on or during admission to the hospital<br>2. Age between 4 months (inclusive) and 59 months (inclusive)<br>3. Body weight >5kgs<br>Screening (in-hospital)<br>1. Fulfilled the pre-study screening eligibility criteria<br>2. Clinically stable, able to switch to oral medication<br>3. Subject completed blood transfusion(s) in accordance with routine hospital practice<br>4. Able to feed (for breastfed children) or eat (for older children)<br>5. Absence of known cardiac problems<br>6. Provision of informed consent by parent or guardian<br>Randomization (at discharge)<br>1. Fulfilled screening eligibility criteria<br>2. Still clinically stable, able to take oral medication, able to feed (for breastfed children) or eat (for older children) and able to sit unaided (for older children who were able to do so prior to hospitalization
Exclusion Criteria	Pre-study screening

	1. Recognised specific other cause of severe anaemia (e.g. trauma, haematological malignancy, known bleeding disorder)<br>2. Known sickle cell<br>3. Child will reside for more than 25% of the 3.5months study period (i.e. 3 weeks or more) outside of catchment area<br>Enrolment in the study (t=0) at discharge<br>4. Previous enrolment in the present study<br>5. Known hypersensitivity to study drug<br>6. Sickle cell disease<br>7. Known need at the time of enrolment for concomitant prohibited medication during the 14 weeks PMC treatment period.<br>8. On-going or planned participation into another clinical trial involving on-going or scheduled treatment with medicinal products during the course of the study (3.5 months from enrolment)<br>9. Known need, or scheduled surgery during the course of the study (3.5 months)<br>10. Suspected non-compliance with the follow-up schedule<br>11. Known heart conditions, or family history of congenital prolongation of the QTc interval<br><br>HIV infection and cotrimoxazole prophylaxis are not exclusion criteria
Primary Outcome	100% of PMC drugs uptake (defined as administration of **all** 3-day treatment courses, given 2, 6 and 10 weeks after discharge) assessed by unannounced spot checks
Key secondary Outcomes	Adherence outcomes<br>1. 60% of PMC drugs (defined as administration of 6 or more [but less than 9] of the daily dosages out of the total of 9, given 2, 6 and 10 weeks after discharge<br>2. 30% of PMC drugs (defined as administration of 3 or more [but less than 6] of the daily dosages out of the total of 9, given 2, 6 and 10 weeks after discharge

	3. <30% of PMC drugs (defined as administration of less than 3 of the daily dosages out of the total of 9, given 2, 6 and 10 weeks after discharge<br>Clinical outcomes<br>4. all-cause mortality<br>5. Incidence rate of all-cause hospital readmission<br>6. Incidence rate of readmissions due to severe anaemia (Hb <5g/dL) or severe malaria (parenteral quinine or artesunate)<br>7. Incidence rate of non-severe all-cause sick-child clinic visits<br>8. Incidence rate of clinic visit due to RDT/microscopy confirmed non-severe malaria<br>Cost-effectiveness outcomes<br>9. cost of delivering the PMC services (providers perspective)<br>10. cost of receiving the PMC services (patients perspective)<br>Feasibility and acceptability outcomes<br>11. the acceptability of PMC<br>12. adaptations to their working practices required to implement PMC<br>13. perceptions of the feasibility of implementing PMC through different delivery mechanisms

4.2 Narrative Protocol Summary

Title: Malaria Chemoprevention with monthly treatment with dihydroartemisinin-piperaquine for the post-discharge management of severe anaemia in children aged less than 5 years in Malawi: A 3-year, single site, parallel-group, five-arm cluster randomised trial of community versus health facility-based delivery mechanisms with or without mobile phone SMS reminders

Short Title: Post-discharge Malaria Chemoprevention Delivery (PMC) study

Background and rationale: Children hospitalised with severe anaemia in Africa are at high risk of readmission or death within 6 months after discharge. No strategy specifically addresses this post-discharge period. In Malawi, 3 months of post-discharge malaria chemoprevention (PMC) with monthly 3-day treatment courses of artemether-lumefantrine (AL) in children with severe malarial anaemia prevented 31% of deaths and readmissions. The effect was in addition to the effect of insecticide-treated bednets. There is now need to design and evaluate effective delivery mechanism for PMC within the health system.

Objectives: The primary objective of the trial is to determine the optimum PMC delivery mechanism by comparing community- versus health facility-based strategies in order to inform policy

Study Type: This is a single-centre, matched, cluster randomized, 5-arm, factorial design trial comparing the uptake of PMC-DHP delivered through health facility or community-based approaches with or without SMS/HSA reminders.

Site: 90 villages in the catchment areas of Zomba Central hospital in southern Malawi

Study Population: Inclusion criteria: convalescent children aged less than 5 years and weighing >5 kg admitted with severe anaemia (haemoglobin<5g/dL / Ht<15%); clinically stable, able to take or switch to oral medication; post-transfusion Hb >5g/dL. Exclusion criteria: blood loss due to trauma, malignancy, known bleeding disorders or sickle cell trait, known hypersensitivity to study drug, known heart conditions, non-resident in study area, previous participation in study, known need at enrolment for prohibited medication and scheduled surgery during the course of the study. HIV infection and cotrimoxazole prophylaxis are not exclusion criteria

Study Interventions: All children will receive Dihydroartemisinin-piperaquine (3-day treatment courses, given 2,6 and 10 weeks after discharge) either: a) at discharge + SMS Reminder; b) at discharge + No SMS Reminder; c) at discharge + HSA Reminder; d) at OPD + SMS Reminder; or e) at OPD + No SMS Reminder

Outcome Measures: Primary: 100% of PMC drugs uptake (defined as administration of **all** 3-day treatment courses, given 2, 6 and 10 weeks after discharge) assessed by unannounced spot checks.

Follow-up procedures: Children will be followed up for 15 weeks by passive case detection in 2 phases: Pre-PMC (2 weeks between hospital admission and 2 weeks post-discharge); PMC (2-14 weeks post-discharge)

Sample Size: A sample size of 75 children per arm (375 total children) allows for a detection of25% increase in uptake from 50% to 75% with 10% loss to follow-up (power 90%, α=0.05).

Data Analysis: The % of children receiving IPTpd according to schedule will be compared by relative risks (95% CI), adjusted for prognostic factors at baseline using log binomial or Poisson regression with adjustment for cluster effects

Partner Institutions: University of Bergen; Makerere University; KEMRI-CDC, Kenya; University of Minnesota; Liverpool School of Tropical Medicine; London School of Hygiene & Tropical Medicine; Imperial College London; University of Amsterdam; US Centers for Disease Control and Prevention, University of North Carolina, and University of Massachusetts.

Table1 : Study treatment and phases

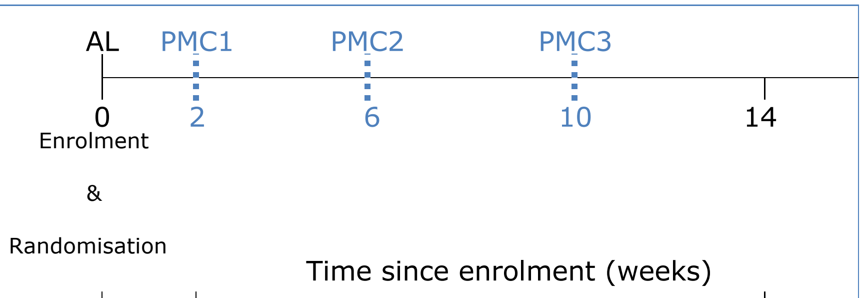

Period	Pre-PMC	PMC
Trial intervention	AL near discharge, all arms	Gp1: PMC Drugs at discharge + No SMS Reminder<br>Gp2: PMC Drugs at discharge + SMS Reminder<br>Gp3: PMC Drugs at discharge + HSA Reminder<br>Gp4: PMC Drugs at OPD + No SMS Reminder<br>Gp5: PMC Drugs at OPD + SMS Reminder
Rescue Rx acute malaria	Oral QN, 7 days	AL, 3 days
Treatment for severe malaria	Parenteral artesunate / quinine and oral AL, 3 days	

An end of study assessment will be done 14 weeks (3.5 months) after discharge.
AL=artemether-lumefantrine. PMC=Post-discharge Malaria Chemoprevention.
DP=dihydroartemisinin-piperaquine. QN=quinine

Table 2: Study Design and Schedule of Assessment

**Phase**	**Screening Phase**	**In-patient Hospitalisation phase**	**Enrolment & Randomisation**	**PMC Treatment Phase 12 weeks period from 2-14 weeks**									**Post-intervention**
**Location**	**In-Hospital**		**Clinic**	**Home/OPD**									**Clinic**
Visit number			#1	#2			#3			#4			#5
Visit description	Pre-study Screening	Screening Consent & Base-line	t=0 weeks; Allocation	t=2 weeks; DHP 1			t=6 weeks DHP 2			t=10 weeks DHP 3			Study end visit
Study Time				2 weeks (day 14 +/- 3)[c]			6 weeks (day 42 +/- 4)[c]			10 weeks (day 70 +/- 4)[c]			14 wks (day 98 +/- 7)
	Days -4[a]-0	Day0		2w-Day 1	2w-Day2	2w-Day3	6w-Day1	6w-Day2	6w-Day3	10w-Day1	10w-Day2	10w-Day3	
Recruitment													
Pre-screening eligibility	X												
Prior consent discussion	X												
Enrolment													
Eligibility screen		X											
Informed Consent			X										
Study code issued			X										
Allocation			X	X									
Interventions													
Drugs at discharge + No SMS				X	X	X	X	X	X	X	X	X	
Drugs at discharge + SMS				X	X	X	X	X	X	X	X	X	
Drugs at discharge + HSA				X	X	X	X	X	X	X	X	X	
Drugs at OPD + No SMS				X	X	X	X	X	X	X	X	X	
Drugs at OPD + SMS				X	X	X	X	X	X	X	X	X	
Iron supplement.				Iron for 28 days from t=14-42 days									

**Phase**	**Screening Phase**	**In-patient Hospitalisation phase**	**Enrolment & Randomisation**	**PMC Treatment Phase 12 weeks period from 2-14 weeks**	**Post-intervention**

Location	In-Hospital		Clinic	Home/OPD									Clinic
Visit Number			#1	#2			#3			#4			#5
Visit description	Pre-study Screening	Screening Consent & Base-line	t=0 weeks; Allocation	t=2 weeks; DHP 1			t=6 weeks DHP 2			t=10 weeks DHP 3			Study end visit
Study Time	Days -4[a]-0	Day0		2 weeks (day 14 +/- 3)			6 wks (day 98 +/- 7)			10 weeks (day 70 +/- 4)			14 wks (day 98 +/- 7)
				2w-Day1	2w-Day2	2w-Day3	6w-Day1	6w-Day2	6w-Day3	10w-Day1	10w-Day2	10w-Day3	
Assessments													
Copy Clinic/Lab data from hospital records		X	X										
Physical Exam.		X	X										X
Blood sample MPs, Hb			X[c]										
**Outcomes**													
PMC uptake				X	X	X	X	X	X	X	X	X	
Clinic visits, Adverse events			Passive surveillance in clinics in the catchment area, 26 weeks from 0-26 weeks (clinical malaria and other acute illnesses) (RDT/smear, Hb, and other tests as required)										
Hospitalisation			Passive surveillance for hospital admission in the catchment area, 26 weeks from 0-26 weeks										
Vital status			X	X	X	X	X	X	X	X	X	X	X
Patient costs			X										X

Visit #1: Enrolment and randomisation at discharge
Visit #2 #3#4: Home visits at 6, 10 and 14 weeks after enrolment to assess uptake of the 1st, 2nd and 3rd treatment course of the PMC study drugs respectively.
Visit #5: at 3.5 months after enrolment. This is the close out assessment.

---

a. Children can be pre-study screened any time between hospital admission and enrolment. The figure of -4 days is provided for illustration purposes only.
b. Visit window= number of days an actual subject visit may fall outside of the planned protocol schedule visit to still meet protocol requirements. DP should be given at least 4 weeks apart.
c. MS, malaria smear. This will be collected for research purposes only, and read days to weeks later. Malaria smears will not be used for point of care. If participants are symptomatic (e.g. fever) an RDT will be taken for point of care.
d. VP=vena puncture. FP=finger prick, DP=dihydroartemisinin-piperaquine, , Hb=haemoglobin, MS=malaria smear, Pf=Plasmodium falciparum

6 BACKGROUND

Children hospitalized with severe anaemia in Africa are at high risk of readmission or death within 6 months after discharge. Malaria remains a major cause of severe anaemia in these settings [1]. A recent multi-centre, randomized, placebo- controlled trial was carried out to determine the impact of administering malaria chemoprevention with Intermittent Preventive Therapy post-discharge (IPTpd) on mortality and readmissions due to severe anaemia among 1441 children <5 years of age with severe anaemia in Malawi [2]. The findings showed that provision of 3 months of chemoprevention with 3 full treatment courses of Artemether Lumefantrine (AL), given in-hospital for initial malaria episode and at 1 and 2 months post-discharge, prevented 31% (95% CI 5-50, P=0.02) of deaths or readmissions due to severe anaemia or severe malaria (composite primary endpoint) by 6 months post-discharge and 41% by 3 months (95% CI 10-62, P=0.014). The beneficial effect was in addition to the initial effect from the standard AL treatment course provided at discharge and in addition to any protective effect by insecticide treated nets (ITNs) [2]. These results are consistent with earlier findings from The Gambia, which showed that in children with severe anaemia, chemoprevention (as monthly IPT with SP or as weekly prophylaxis with Pyrimethamine-Dapsone) targeted during the malaria transmission season halved the rate of clinical malaria and reduced all-cause hospital readmission by 78% in one trial, and recurrence of severe anaemia by 78% in the other [3, 4]. These data are scarce, but indicate that IPTpd in the post-discharge period may potentially provide substantial health benefits.

IPT is the administration of a full treatment course of long-acting antimalarials at pre-defined time intervals irrespective of a patient’s malaria status. IPT clears existing infections and provides prolonged prophylaxis against new infections [5]. The World Health Organisation’s (WHO) recommended malaria control strategies include IPT for pregnant women (IPTp) and for infants (IPTi) and children living in areas with seasonal malaria transmission (IPTc).

6.1 High risk of severe anaemia rebound after discharge:

Severe anaemia is a leading cause of hospital admissions in Africa contributing substantially to paediatric mortality. Our recent case-control study in Malawian children indicated that children aged <5y admitted with severe anaemia are not only at high risk of dying during the acute phase in-hospital (6%) but also for several months after they leave hospital: 8% had died by 6 months post-discharge, which is nine times higher than the mortality in community-based, age matched children with mild anaemia [1, 6]. A further 8% were readmitted with rebound severe anaemia [6]. Similar rates of post-discharge morbidity and mortality are seen in western Kenya, where 17.9% of children <5y admitted with severe anaemia died or were readmitted by 6 months (Desai et al, unpublished observations), while in Uganda as many as 36.5% died or were readmitted within 6 months (C. John et al, unpublished data). Hospitalised children with severe anaemia are particularly at risk within the first 3 months post-discharge, likely due to a combination of environmental, behavioural, nutritional and genetic risk factors [3, 4, 6].

6.2 Malaria as a cause of post-discharge severe anaemia and mortality

Since most post-discharge mortality in children occurs at home, the causes of death have rarely been assessed. However, previous observational studies in western Kenya and a recent intervention study in a high transmission are in Malawi showed that, malaria in the post-discharge period is an important contributor responsible for a slow haematological recovery, rebound severe anaemia and morbidity. Many children in these areas experience episodes of new or recrudescent malaria infections after discharge which negates the initial rise in haemoglobin (Hb) achieved by blood transfusion in hospital. Haematological recovery rom malaria-associated anaemia is known to take at least 6 weeks. This period may be prolonged in those with persistent or new malaria infections due to on-going red cell destruction and red blood cell production failure.

6.3 Treatment strategies for prevention of malaria and haematological recovery post-discharge:

Standard treatment for severe anaemia in many countries in sub-Saharan Africa (SSA) consists of a blood transfusion combined with (presumptive) IV anti-malarial treatment (quinine or artesunate) plus antibiotics if bacterial infections are suspected. Once children have stabilised and can be switched to oral treatment this is completed with short courses of 3-day treatment with artemisinin-based combination therapy (ACT), usually artemether-lumefantrine (AL). Children are often discharged with a short course of iron and folate, typically with no scheduled follow-up [7].

Creating a prophylactic time-window post-transfusion, is suggested to allow time for the bone marrow to recover, resulting in a more sustained haematological recovery post-discharge. Data from a previous study in Malawi show that this process takes 2-3 months in children with severe anaemia [1]. Recently, the use of IPT in children with severe anaemia during the rainy season reduced clinical attacks of malaria by more than 80% in areas with highly seasonal transmission.

6.4 Proposed PMC delivery mechanisms trial

The PMC delivery mechanisms and health services trial is one of the five Major Activities (MAs) that will address gaps in knowledge before the Global Malaria Programme (GMP) of WHO would consider whether PMC should be recommended as a strategy for the post-discharge management of children with severe anaemia. The main objective is to determine the uptake, effectiveness, cost-effectiveness, acceptability and feasibility of two different mechanisms for delivering IPTpd: facility vs community-based approaches and examining the added role of mobile phone text message reminders.

6.5 Rationale for choice of methods

In the past two decades, most research on severe anaemia and severe malaria focused on reducing in-hospital mortality. Our observations suggest that a major, potentially preventable, component of the burden occurs after discharge and that a proactive approach using PMC could offer substantial public health gains. This is a priority area for research since no strategy specifically addresses this high-risk post- discharge period. Although PMC is a simple intervention, implementation requires appropriate delivery mechanisms as treatment must be administered for 3 days at different pre-specified intervals post-discharge. Whereas IPT strategies in infants and pregnant women are

delivered through the expanded programme on immunisation and antenatal clinics, delivery of PMC will require new systems to be established that are sustainable and cost-effective. Delivery of IPTc to children through community health workers has been shown to be feasible and well accepted in rural West Africa [8, 9]. In Malawi, there are health systems that include village health volunteers (VHVs) and Health Surveillance Assistants who could deliver PMC or schedule post-discharge visits to clinics or hospital outpatient departments for subsequent PMC doses. Since PMC targets a very high-risk group of hospitalised children who already have contact with the health-care system, the point of entry is already established [10].

7 STUDY OBJECTIVE

7.1 Primary objective

The primary objective of the trial is to determine the optimum PMC delivery mechanism by comparing community- versus health facility-based strategies in order to inform policy

7.2 Specific objectives

1. To compare PMC uptake (adherence) levels between community- versus health facility-based strategies
2. To determine whether SMS or Health Surveillance Assistant (HSA) reminder has additional benefit on PMC uptake (adherence) levels
3. To determine the safety of PMC delivery in the community versus in the health facility
4. To determine and compare the cost to the health service of delivering PMC using each individual delivery strategy
5. To determine and compare the cost to the patients and guardians of receiving PMC using individual delivery strategies
6. To determine the feasibility and acceptability of delivery of PMC in a typical Malawi health system setting

7.3 OUTCOMES

7.3.1 Primary outcome

100% of PMC drugs uptake (defined as administration of **<u>all</u>** 3-day treatment courses, given 2, 6 and 10 weeks after discharge) assessed by unannounced spot checks

7.3.2 Secondary outcomes

Adherence outcomes

1. 60% of PMC drugs (defined as administration of 6 or more [but less than 9] of the daily dosages out of the total of 9, given 2, 6 and 10 weeks after discharge
2. 30% of PMC drugs (defined as administration of 3 or more [but less than 6] of the daily dosages out of the total of 9, given 2, 6 and 10 weeks after discharge
3. <30% of PMC drugs (defined as administration of less than 3 of the daily dosages out of the total of 9, given 2, 6 and 10 weeks after discharge

Clinical safety outcomes

4. All-cause mortality
5. Incidence rate of all-cause hospital readmission

6. Incidence rate of readmissions due to severe anaemia (Hb <5g/dL) or severe malaria (parenteral quinine or artesunate)
7. Incidence rate of non-severe all-cause sick-child clinic visits
8. Incidence rate of clinic visit due to RDT/microscopy confirmed non-severe malaria

Cost-effectiveness outcomes

9. Cost of delivering the PMC services (providers perspective)
10. Cost of receiving the PMC services (patients perspective)

Feasibility and acceptability outcomes

11. The acceptability of PMC
12. Adaptations to their working practices required to implement PMC
13. Perceptions of the feasibility of implementing PMC through different delivery mechanisms

8 DESIGN AND METHODOLOGY

8.1 Trial Design

This is a single-centre, matched, cluster randomized, 5-arm, factorial design trial comparing the uptake of PMC-DHP delivered through health facility or community-based approaches with or without SMS/HSA reminders. All children will receive DHP and hence there are no placebo arms in this trial. However, children shall be randomized to receive PMC as follows:

a. *Community-based*: Mother given **all** PMC drugs at discharge without an SMS reminder
b. *Community-based*: Mother given **all** PMC drugs at discharge with an SMS reminder
c. *Community-based*: Mother given **all** PMC drugs at discharge with HSA reminder
d. *Facility-based*: Mother asked to return to outpatient departments (OPD) for each **monthly** PMC without an SMS reminder
e. *Facility-based*: Mother asked to return to outpatient departments (OPD) for each **monthly** PMC with an SMS reminder

Where;

Arm 1: **Mother given at discharge without SMS reminder:** At discharge, the OPD staff will give the mother or guardian all drugs for PMC-1, PMC-2 and PMC-3 and instruct mother/guardian when to give these drugs.

Arm 2: **Mother given at discharge + SMS reminder:** At discharge, the OPD staff will give the mother or guardian all drugs for PMC-1, PMC-2 and PMC-3 and instruct mother/guardian when to give these drugs. Additionally they will be reminded via SMS to give the drugs to the child a date before each treatment course is due.

Arm 3: **Mother given at discharge + HSA reminder:** At discharge, the OPD staff will give the mother or guardian all drugs for PMC-1, PMC-2 and PMC-3 and instruct mother/guardian when to give these drugs. Additionally HSA or Village Health Volunteers (VHV) which are part of existing networks of community based volunteers taking part in village health committees will be reminded via SMS to go and remind the mother to give the drugs to the child a date before each treatment course is due.

Arm 4: **Mother given monthly at OPD without SMS reminder**: At discharge, the mother will be requested to return to the OPD to collect drugs for PMC-1, PMC-2 and PMC-3.

Arm 5: **Mother given monthly at OPD + SMS reminder**: At discharge, the mother will be requested to return to the OPD to collect drugs for PMC-1, PMC-2 and PMC-3. Additionally they will be reminded via SMS to come to the clinic to collect drugs a date before each treatment course is due.

8.1.1 Rationale for the trial arms

There is need to assess a number of strategies for effective delivery of PMC in a largely rural community that would typically benefit from this intervention. Presently in African health systems and particularly Malawi there is no post-discharge health management system. Most patients once discharged are no longer expected to have contact with the health system apart from for EPI visits (if less than 9 months old). Post-discharge health care becomes very important in severe anaemia as it has been shown that they are at particular risk of dying or having another episode of severe anaemia.

We postulate that this post-discharge care in the form of PMC may be delivered either in the community or at a health facility. The most likely scenario where the health care provider is least involved would be where mothers are given PMC drugs on discharge from hospital and allowed to administer the drugs on her own to the child (Arm 1). However as we postulate that the mother could forget to administer the PMC, we would like to test a number of reminder systems. The first is through the use of SMS technology, which has been shown to be user friendly and acceptable from our own pilot work and other programs in Malawi that are currently using it (Arm 2). FrontlineSMS™ is a simple and free desktop software that enables instantaneous 2-way communication to any mobile handset. It is easy to implement, simple to operate and the only cost is for the sms that is sent (www.frontlinesms.com).

An alternate reminder system is the use of HSA. HSA are Ministry of Health (MoH) employees who are responsible for basic health promotion activities in the community. Ideally MoH strives to have 1 HSA for every 1000 people, but in reality they often cater for much larger populations. As they are the main MoH community health workers they are usually over burdened with many disease control programs. In some areas in rural Malawi there are Village Health committees, which are made up of voluntary members of the community. They work hand in hand with the HSA. We postulate that HSAs and where available village health volunteers (VHVs) are an option for reminding mothers to give PMC to their child (Arm 3).

Alternative delivery strategies for PMC are to have the mother return to the health care facility to receive drugs for each treatment course. This is a plausible strategy as this occurs in many chronic illness cases where drugs are supposed to be collected from a health facility (e.g. in TB, HIV). In recent years there has been successful disease control programs operationalizing more decentralised drug delivery programs in the community. In this trial the mother may be reminded to come collect their child's PMC drugs using SMS (Arm 4) or not (Arm 5).

Figure 1: Study Arms

		Mode of Reminder		
		**No SMS**	**SMS**	**HSA Reminder[3]**
**Mode of delivery of PMC**	**All at Discharge[1]**	**Group 1**	**Group 2**	**Group 3**
	**Monthly at OPD[2]**	**Group 4**	**Group 5**	

[1]Mothers given all PMC treatment courses at discharge

[2]Mothers return to OPD for each PMC treatment course

[3]HSA is requested to remind mother for each PMC treatment course at home

8.1.2 Sub-studies

The cost of delivering the intervention from the providers perspective and the cost of receiving the intervention from the patients perspective will be assessed through a Economic evaluation sub study involving a sub set of patients participating in the main malaria chemoprevention post-discharge delivery mechanisms trial**.**

A health services qualitative study will be conducted on a subset of participants in the main delivery mechanisms trial who will be enrolled towards the end in order to assess the level of acceptability of PMC; adaptations to health workers working practices to implement the intervention; perceptions of the feasibility of implementing PMC through different delivery mechanisms and come up with a list of recommendations on effective implementation.

8.2 STUDY SITE

90 villages in the catchment areas of Zomba Central hospital in southern Malawi. This hospital participated in previous related studies [1, 2, 6] and is particularly suitable for the delivery trial because of the existing linkages with community based health care providers and rural clinics within their catchment areas. In addition, it is close to the College of Medicine where procurement of study resources will be done.

8.3 SAMPLE SIZE

Although a factorial design will be used, only the main effects of PMC mode of delivery and the "use of SMS/HSA reminders" are of primary interest. There are five arms in total as described above such that each of the four intervention arms will be compared to the control arm (Arm 1: Mother given at discharge without SMS reminder). Each village cluster contributes 2 to 4 children/year on average with severe anaemia. Thus, the cluster sizes vary and we have accounted for this in sample size calculations in order to achieve the number of clusters that would have enough power to detect the desired difference. The sample size calculation has been adjusted in the design effect using the coefficient of variation method [11-13]. This has assumed that the cluster sizes will be uniformly distributed between 2 and 4 children i.e. U[2,4]. This gives a mean cluster size of 3 children per village per year and a standard deviation of cluster sizes of 0.58. Hence the coefficient of variation of cluster sizes (CV) is 0.58/3= 0.19 and CV^2=0.036. Assuming an

intra-cluster correlation coefficient (ICC) of 0.1 and allowing for 10% loss-to-follow-up, a sample size of 25 clusters of an average of 3 children per village (75 children per arm, N=375 overall (for 125 clusters for the 5 arms)) has 80% power to detect a 25% absolute increase in uptake from an estimated 50% in the OPD and delivery at home groups to 75% in the arms supported by SMS reminders (α=0.05). The ICC of 0.1 is slightly more conservative than the ICC in a previous trial of delivery approaches for IPTc in the Gambia (0.08)[14].

8.4 DATA COLLECTION TOOLS

Study instruments will include structured forms for clinical history, physical assessment, laboratory evaluations and information about expenses, resource and time use, with open-ended fields for unanticipated findings and place for comments. In addition there will be interview guides for In-depth Interviews and Focus Group Discussions. Prior to data collection these will be translated into Chichewa language and back-translated for accuracy. The forms will be administered to the participants in Chichewa which is the national language but also a common language in the study area.

8.5 VARIABLES

The study will include a range of variables, identified both at the time of enrolment and at follow-up. Demographic information on children including age, sex, religion, village of residence and socioeconomic features of household will be collected, as well as cost information for the economic evaluation. Information to assess the acceptability of PMC, adaptations to health workers' working practices required to implement PMC, perceptions of the feasibility of implementing PMC through different delivery mechanisms, and recommendations on effective implementation will also be collected. Clinical information collected on history of current and previous illnesses and hospital admissions will be collected in addition to hospitalisations and mortality. For children who are hospitalized during the follow-up period, data will be collected on the length of hospitalization, diagnosis, treatments provided, laboratory results, and participant outcome. Physical exam and laboratory test results collected will include: height, weight, Mid Upper Arm Circumference (MUAC), Malaria rapid testing (mRDT) results, blood slide results, parasitaemia level, and haemoglobin level.

8.6 TRIAL MEDICATION AND INTERVENTIONS

8.6.1 Study Medications

8.6.1.1 Dihydroartemisinin-piperaquine

We shall use the GMP formulation of Dihydroartemisinin-piperaquine (DHA): Eurartesim®, a GMP certified product from Sigma Tau and approved by the European Medicines Agency (EMEA) in October 2011. Eurartesim is a co-formulated tablet containing 40 mg dihydroartemisinin and 320 mg piperaquine phosphate or as 20/180 (paediatric formulation).

Dosing will be by bodyweight according to the following schedule
recommended by the manufacturer (

Table 4: Dihydroartemisinin-piperaquine (Eurartesim®) weight-based dosing schedule

). WHO is in the process of revising the dose recommendations for DP in this age group. We will utilize the new dose recommendations as soon as WHO will make these available.

Table 4: Dihydroartemisinin-piperaquine (Eurartesim®) weight-based dosing schedule

Weight in Kg	Daily dose (mg)		Tablet strength and number of tablets per dose
	Piperaquine	DHA	
**5 to <7**	80	10	½ x 160 mg / 200 mg tablet
**7 to <13**	160	20	1 x 160 mg / 200 mg tablet
**13 to <24**	320	40	1 x 320 mg / 400 mg tablet
**24 to <36**	640	80	2 x 320 mg / 400 mg tablet

If the paediatric strength tablets are not available, the full tablet strength can be used to provide the equivalent dose in mg (i.e. ¼ tablet and ½ tablet of 320 / 40 mg tablets for the first two weight bands)

8.6.2 Drug administration

For all the OPD arms, the first dose will be given as a crushed powder mixed with water at the OPD. The remaining two doses will be given to take at home during the following two consecutive days. Participants in other study arms will take all the drugs at home. Participants will be advised to give the drug to the child at about the same time each day with water. Should a caretaker forget to give the drug dose at the set time, they will be advised to take it as soon as realised and to continue the recommended regimen until the dose is completed.

Participants who take the Eurartesim at the OPD will be observed for 60 minutes. Should a participant vomit within 30 minutes of receiving the treatment drug, the full dose will be re-administered. Should a participant vomit within 30-60 minutes of drug intake, half dose will be re-administered.

To minimise QTc prolongation, the manufacturer of Eurartesim advises patients to take the first day's dose approximately three hours after meals as fatty food can increase the absorption of piperaquine. However, overall studies show that DP is well tolerated and can be given with small amount of food, although trough drug levels are also effective when given to fasting patients.

8.6.2.1 Artemether lumefantrine

Although not part of the study drugs, Good manufacturing Practice (GMP) formulation of Artemether Lumefantrine: Coartem, Norvatis Pharmaceuticals will be administered to all study participants in hospital as part of standard care. AL will be dosed along with a small amount of food, e.g. a biscuit. The recommended treatment is a 6-dose regimen over a 3-day period. The standard tablet or the dispersible tablets containing 20mg of artemether and 120mg of lumefantrine will be used and dosed according to body weight as per WHO dosing recommendation as provided in the latest WHO malaria treatment

guidelines. The dose regimen will be updated if and when WHO updates their dose recommendations. The dosing is based on the number of tablets per dose according to pre-defined weight bands and the current recommendations are:

Body Weight	Recommended number of AL tablets
5-14 kg	1 tablet
15-24 kg	2 tablets
25-34 kg	3 tablets
> 34 kg	4 tablets

These doses are given twice a day for 3 days. This extrapolates to 1.7/12 mg//12-mg/kg body weight of artemether and lumefantrine, respectively, per dose, given twice a day for 3 days with a therapeutic dose range of 1.4-4 mg/kg of artemether and 10-16 mg/kg of lumefantrine.

Table 3: Artemether Lumefantrine weight based dosing schedule

Weight in Kg	Number of pediatric tablets of Coartem® per dose					
	Day 1		Day 2		Day 3	
	1st dose	8 hours	24 hours	36 house	48 hours	60 hours
**5 to <15**	1	1	1	1	1	1
**15 to<25**	2	2	2	2	2	2
**25 to ≤34**	3	3	3	3	3	3
**>34**	4	4	4	4	4	4

8.6.3 Information on how to give study drugs at home

Participants in all the five arms will take the drug at about the same time each day. Should mother/guardian forget to give the child the drug dose at the set time, they will be advised to give it as soon as realised and to continue the recommended regimen until the treatment is completed. The mother/guardian will be advised to observe participants for 60 minutes after taking DHP. Should a participant vomit within 30 minutes of receiving the treatment drug, the mother/guardian is instructed to re-administer the full dose. Should a participant vomit within 30-60 minutes of drug intake, half dose will be re-administered. Repeat dosing will be attempted once.

8.6.4 **Procedures for Drug handling & Accountability**

8.6.4.1 Preparation and packaging

All subject-specific study drugs will be prepared and stored in small opaque medicinal containers provided by the sponsor, according to the subject's body weight recorded at enrolment. This will be done by the pharmacy Assistant. Prior to each drug administration, the drugs will be crushed and suspended in the opaque container by adding flavoured syrup.

8.6.4.2 Labelling of the drugs

Labelling will be done in both Chichewa and English. This will include study name, investigative institute, cluster name and study identification number of the participant, staff contact number, drug dose and usage directions and precautionary measures to be observed when taking the drug.

8.6.4.3 Storage of drugs at the study clinic

The study drugs will be stored in a designated study pharmacy in a secure area with access limited to designated trial staff. Appropriate storage conditions as specified on the study drug pack will be observed.

8.6.4.4 Product accountability

The investigator will be responsible for establishing a system for the correct handling of study drug to ensure that:

1. Deliveries of study drug from the sponsor are correctly received by a responsible person (e.g. pharmacist assistant)
2. Accurate records are maintained for the receipt of study drug, for the dispensing of study drug to subjects and for returned drug.
3. Certificates of delivery and return must be signed preferably by the investigator or authorised personnel and copies retained in the investigator file.
4. Study drug is to be handled and stored safely and properly and in agreement with the given storage instructions.
5. The study drug is to be prescribed only by the principal investigator, co-investigators or study site personnel authorised to do so by the principal investigator.
6. Study drug is dispensed only to study subjects in accordance with the protocol.
7. Subjects must return all unused medication and empty containers to the investigator.
8. At the end of the study delivery records must be reconciled with records of usage and returned stock. Any discrepancies must be accounted for in writing.

   Once accounted for any returned and unused study treatment at the site will be returned to the sponsor for destruction or destroyed locally upon agreement with the sponsor. Drug destruction certificates will be issued that refers to the subject study numbers for subject specific medication that was destroyed.

8.6.4.5 Dispensing of trial drug

Designated staff will be responsible for dispensing the drug to mothers/guardians of participants. Each time the drug is dispensed, the amount dispensed, the name of the participant, date and signature of the person dispensing the drug will be recorded in the log file.

8.6.4.6 Iron and folate supplementation

All children in all the five arms, regardless of Hb level, will receive a standardized prophylactic dose of iron supplementation (about 2 mg/kg) from two weeks into the study onwards for a period of about 4 weeks (i.e. until the next dose of PMC-DP is due at 6 weeks). Iron can be given as mono-therapy or as part of the fixed-dose formulation with folic acid.

8.6.5 **Prohibited medications**

Prohibited medication includes antimalarial drugs not prescribed within the trial protocol and other drugs with antimalarial properties.

8.7 SELECTION AND WITHDRAWAL OF STUDY PARTICIPANTS

8.7.1 Inclusion Criteria

8.7.2 Eligibility criteria for pre-study screening

Parents / guardians of children who fulfil the pre-screening eligibility criteria will be informed about the existence of the study by the hospital and/or study staff. Consent will not be obtained at the acute stage of the illness but a few days later preferably at discharge when the child has recovered and is able to switch from parenteral to oral medication (see section below). This provides a time window of 1 to 3 days for the parents or guardian to reflect on the study and discuss it with family members.

Pre-study screening will be done by hospital staff or study staff. No study specific information or samples will be collected in this pre-study screening period. The role of the study team during this period is to review the diagnosis and ensure that the potential study participants get the standard quality of care for severe malarial anaemia.

Each pre-screened subject will be assigned a pre-screening number in sequential order by the hospital regardless of whether they fulfil the pre-screening eligibility criteria. Data will be recorded on a pre-screening log that will be kept in the investigator’s site file. This record will be used to report how many patients were pre-screened and how many were eventually recruited in the study to establish that the study population was selected without bias. This screening log will not contain names or other identifying information

8.7.3 Screening and Consent

The parents of the children will be approached for a screening interview as soon as the child is sufficiently recovered to take oral medication (i.e. after completion of the blood transfusion and the standard parenteral artesunate medication). This is typically within 48 hours following admission to the hospital. During this interview verbal consent will be sought from the parents or guardian and if granted the eligibility criteria for inclusion in the study will be assessed.

8.7.3.1 Informed Consenting process

The participant will be given the informed consent form translated in Chichewa language. In the case of an illiterate carer, the study staff will read the consent form to the prospective participant in the presence of a witness who is not part of the study. The witness will sign the consent form to attest that information in the consent form was accurately explained and that informed consent was freely given. Study staff will answer

any questions prior to asking the carer to consent. The consent form will be translated to Chichewa from English and then back translated to verify accuracy.

If a carer is illiterate, a thumbprint will be accepted as a legally effective signature. Carers will be advised that they are free to decline any question or procedure and that they may terminate their participation at any time without loss of any benefits and usual child care they receive at the hospital. Consent will be signed either with carbon copies or otherwise with two identical consent form per carer. One signed or thumb printed consent form will be kept on file by the study team and one will be given to the participant.

The consenting process will take place at the study clinic.

8.7.3.2 Screening log

The investigator will keep a subject screening log for all subjects considered for enrolment regardless of whether they were enrolled, which combined with the pre-screening log will be used to establish that the study sample was selected without bias. This screening log will not contain names or other identifying information.

8.7.4 **Enrolment**

Once the parent/guardian provides informed consent for his/her child to participate in the study, the participant will be assigned a study ID and randomised to one of the five study arms.

8.7.4.1 Assignment of study IDs

Pre-screened subjects who meet all eligibility criteria will be issued a study subject number during this visit. Once issued the study subject number they will be considered as ‘enrolled. This number is the subject's unique identifier and used to identify the subject on the CRFs. Subject numbers will be assigned strictly sequentially as subjects enter the study. Once a number has been assigned no attempt will be made to use that number again, for example even if a subject discontinues or is a screening failure.

8.7.4.2 Randomisation procedure at discharge

The unit of randomization will be the villages, defined as the smallest administrative unit overseen by a Village headman. A group Village headman oversees several villages. Ninety (90) villages in the catchment areas of Zomba district located in the southern region of Malawi will be involved in the study. Zomba Central Hospital participated in previous related studies [1, 15-17] and is particularly suitable for the delivery trial because of the existing linkages with community based health care providers and rural clinics within their catchment areas. A 1:1 allocation ratio will be used. To minimize imbalances across groups with respect to baseline malaria prevalence and risk factors for malaria, location-specific baseline information on malaria indicators, such as the prevalence of positive RDTs or microscopy in the 12-month period prior to the trial (Under-five registry data) will be considered in selection of villages.

Randomisation will be done by opening a single concealed envelope, which will contain the cluster and study arm.

8.7.4.3 Clinical assessment:

After consent is obtained and the subject's eligibility is confirmed the subject's demographic data will be recorded in the CRF, and all relevant clinical information, including the previous and current medical history, and laboratory information copied from the hospitals clinic and laboratory notes to the CRF (see CRF for details). A further clinical examination (including anthropometrics) will be performed and a medical history taken that will serve as the baseline examination and captured on the CRF.

Inclusion criteria for enrolment into pre-study screening period

1. Haemoglobin <5.0g/dl or PCV <15%, or requirement for blood transfusion for other clinical reasons on or during admission to the hospital
2. Age between 4 months (inclusive) and 59 months (inclusive)
3. Body weight >5kgs

Exclusion criteria for enrolment into pre-study screening period

1. Recognised specific other cause of severe anaemia (e.g. trauma, haematological malignancy, known bleeding disorder, known sickle cell disease)

8.7.4.4 Eligibility criteria for enrolment into study

Inclusion criteria

1. Fulfilled the pre-study screening eligibility criteria
2. Clinically stable, able to switch to oral medication,
3. Subject completed blood transfusion(s) in accordance with routine hospital practice
4. Able to feed (for breastfed children) or eat (for older children)
5. Able to sit unaided
6. Provision of informed consent by parent or guardian

Exclusion criteria

1. Previous enrolment in the present study
2. Used DHP since screening
3. Fever (>37.5 °C) on the day of randomisation
4. Known hypersensitivity to study drug
5. Child resides outside catchment area during the course of the study (6 months)
6. Known need at the time of enrolment for concomitant prohibited medication during the 14 weeks PMC treatment period
7. On-going participation into another clinical trial involving ongoing or scheduled treatment with medicinal products during the course of the study (6 months)
8. Suspected non-compliance with the follow-up schedule
9. A known need for scheduled surgery during the course of the study (6 months)
10. Suspected non-compliance with the follow-up schedule

HIV infection and cotrimoxazole prophylaxis are not exclusion criteria

8.7.4.5 Location of household and issuance of study ID card

A detailed route description to locate the household of the participant including sketching of a map will be taken, to aid the planned spot checks to assess uptake of PMC at home. Lastly a subject's identification card will be issued. A GPS will be used to collect the exact location of the participant's house.

8.7.5 Unscheduled visits (passive follow-up)

8.7.5.1 Inter-current illness

A passive surveillance system will be used to monitor inter-current illnesses through the research clinic. Parents will be instructed to bring their child to the research clinic for any suspected illness. The 'problem' (reason for attending the clinic during passive surveillance) and 'diagnosis' will be recorded using a standard list based on the International Classification of Disease (ICD-10) for children [18]. Blood samples for malaria smears ,haemoglobin and dried blood spot on filter paper will be taken if clinically indicated.

Information regarding economic costs will also be collected during this visit. Provider costs such as time spent for registration, time spent with the nurse and time spent with a physician will be measured. In addition, all equipment usage and medicines will be identified and retrospectively costed using unit prices. Economic costs to the patient will also be collected; costs regarding travel time to the facility, travel costs, waiting time, among others.

8.7.5.2 Verbal autopsy visit

All parents of children who die will be advised to report the incident to the study clinic/study staff/HSA as soon as possible for a detailed verbal autopsy interview that will be used to categorize the potential cause of death as probable, possibly, or not malaria related.

8.7.6 Adherence to study intervention protocol and strategies for retention

8.7.6.1 Adherence to study protocol and medication

At 2, 6 and 10 weeks mothers will be asked to administer already received PMC drug or asked to come to the clinic to receive the drugs. At discharge all mothers regardless of the arm they have been randomised to will be instructed on how to administer DHA to their child. Nurses will demonstrate this by using dummy tablets (placebos) and later the mothers will be asked to do it under supervision. Mothers will be given adequate time to ask for any clarifications during this process. Mothers will also be informed to report to the hospital for any adverse events that the child might experience whether they suspect they are related or not to the study drug.

At discharge, after randomisation, all mothers will be informed of the dates for PMC. These dates will be written in the child's Health Passport and they will additionally be provided with a laminated study card which will contain the Study ID of the child, Name of the child and PMC-1, PMC-2 and PMC-3 dates.

8.7.6.2 Strategies for retention

During screening, parents will be asked whether the child will travel out of the study area for an extended period during the follow-up period. Those who plan 1 month or more away from the study catchment area will be excluded from enrolment.

Detailed directions to the children's homes as well as contact information, including mobile phone information, will be recorded prior to discharge. If children do not return for the Study End visit at 15 weeks, the study team will call them and ask them to come to the clinic for evaluation, offering transport reimbursement, or may visit their house to help arrange transport to the clinic if they are willing to come to the clinic, or, alternately, a study staff may go to their home for Study End visit evaluation.

The caretaker and participant's travel costs will be reimbursed.

8.7.7 **Prior and concomitant therapy**

All concomitant medications taken during the study will be recorded in the appropriate sections of the CRF with indication, dose information, and dates of administration.

8.7.7.1 Permitted Medications during follow up period

During the treatment and follow-up phase of the study if a subject is diagnosed with malaria the investigator will prescribe antimalarial treatment based on the severity of the malaria illness.

Use of cotrimoxazole (which has some antimalarial properties) treatment or prophylaxis is not considered prohibited as short-courses are commonly prescribed for the treatment of bacterial infection. Daily cotrimoxazole prophylaxis is used by HIV-infected and exposed children.

8.7.7.2 Concomitant and prohibited Medications

Participants will be counselled to avoid concomitant and prohibited medications, specifically antimalarial drugs not prescribed within the trial protocol, or drugs that may be associated with QTc prolongation.

Prohibited medication

- Antimalarials not prescribed by the study: Chloroquine, halofantrine, mefloquine
- Diuretics (hydrochlorothiazide, furosemide)
- Drugs known to prolong the QT interval
- Antimicrobials:
    - macrolides (e.g. erythromycin, clarithromycin, azithromycin, roxithromycin),
    - fluoroquinolones (e.g. ciprofloxacin, norfloxacin, levofloxacin, moxifloxacin, sparfloxacin)
    - pentamidine
- Antifungals: ketoconazole, fluconazole, Itraconazole, posaconazole, voriconazole, caspofungin
- Antiretrovirals: ARVs, specifically: indinavir, nelfinavir, atazanavir, saquinavir
- Antiarrhythmic agents (e.g. amiodarone, sotalol)
- Non-sedating antihistamines (astemizole, terfenadine)
- Antipsychotics (neuroleptics): Haloperidol, Thioridazine
- Antidepressants: Imipramin, Citalopram, Escitalopram

- Antiemetics: Domperidone, Chlorpromazine, Ondansetron

Randomised participants, who take prohibited medications resulting in the premature cessation of the study intervention, will remain in the trial and will be included in the primary, intention-to-treat analysis, but excluded from the per-protocol analysis.

8.8 PARTICIPANT WITHDRAWAL

Patients can discontinue from the study for any one of the following reasons.

1. Screening error resulting in incorrect enrolment (found that subject did not meet required inclusion / exclusion criteria)
2. Withdrawal of consent at any stage or subject not willing to continue in the study / voluntary discontinuation by the subject
3. Suspected or confirmed allergic reaction to the study drug (removal from therapy only)
4. Safety reasons as judged by the investigator, study safety monitor or DMEC (removal from therapy only)
5. Other

The parents or guardian of subjects who discontinue from the study treatment or from the study entirely will always be asked about the reason(s) for their discontinuation and the presence of adverse events. If a subject discontinues it should be established whether the subject:

1. Discontinues the study treatment, but continues their consent for the data capture up to that point, and to continue follow-up. These subjects will be considered 'off study drug/on study' and where feasible will follow the same schedule of events as those who continue the study intervention, except any adherence assessment. All of these children will be followed until study end at 6 months from enrolment.
2. Discontinues all future activities in the study, but continues their consent for the data captured up to that point to be used in the research
3. Discontinues all future activities in the study and withdraws consent for any data captured to be used for the research

Every effort will be made to follow-up patients who discontinue due to drug related adverse events in order to determine the final outcome. If a subject discontinues due to drug-related adverse events, all the assessments will be conducted that would have been carried out at the next scheduled visit at 6 month (unless consent is withdrawn). This will be recorded in the Case Record Forms (CRFs). The study drug will be returned by the subject. Subjects that have discontinued the study prematurely will not be replaced.

8.9 OUTCOMES

8.9.1 Primary Outcome

The primary outcome is the 100% of PMC drugs uptake (defined as administration of **all** 3-day treatment courses, given 2, 6 and 10 weeks after discharge) assessed by unannounced spot checks.

The PMC uptake will be assessed through a study questionnaire administered at unannounced spot checks to determine if and when children and their mothers/guardians attend the scheduled dosing of the drug. The spot checks will be conducted at day 4 or 5

of each DHP treatment course. The effectiveness of the delivery mechanism will be determined by the following measures:

1. **Pill count**: Research Assistants will perform a pill count of the drugs available at the day of the visit. This will be used to assess whether participants took the whole course of DHP or not.
2. **Interview**: A face-to-face interview with the mothers/guardians of children participating in the study will be done to collect information on missed doses and possible reasons for any missed doses.

8.9.2 Secondary Outcomes

8.9.2.1 Adherence outcomes

1. 60% of PMC drugs (defined as administration of 6 or more [but less than 9] of the daily dosages out of the total of 9, given 2, 6 and 10 weeks after discharge
2. 30% of PMC drugs (defined as administration of 3 or more [but less than 6] of the daily dosages out of the total of 9, given 2, 6 and 10 weeks after discharge
3. <30% of PMC drugs (defined as administration of less than 3 of the daily dosages out of the total of 9, given 2, 6 and 10 weeks after discharge

8.9.2.2 Clinical outcomes

1. all-cause mortality
2. Incidence rate of all-cause hospital readmission
3. Incidence rate of readmissions due to severe anaemia (Hb <5g/dL) or severe malaria (parenteral quinine or artesunate)
4. Incidence rate of non-severe all-cause sick-child clinic visits
5. Incidence rate of clinic visit due to RDT/microscopy confirmed non-severe malaria

8.9.2.3 Cost-effectiveness outcomes

1. cost of delivering the PMC services (providers perspective)
2. cost of receiving the PMC services (patients perspective)

8.9.2.4 Feasibility and acceptability outcomes

1. the acceptability of PMC
2. adaptations to their working practices required to implement PMC
3. perceptions of the feasibility of implementing PMC through different delivery mechanisms

8.10 EXPECTED DURATION OF TRIAL

The total duration of the trial for a participating mother/guardian-child pair is 3.5 months. Participant recruitment will occur for 30 months. There will be 4 months of follow-up of the last recruited subject and finally 6 months to complete data analysis and study reporting. The end of trial will be defined as the final assessment of the last participant within the trial, unless additional time is needed for follow-up of a trial-associated adverse event. This new protocol is a continuation of COMREC study P.02/15/1679.

8.11 PROCEDURES FOR RECORDING AND REPORTING ADVERSE EVENTS

8.11.1 Reporting to sponsor

Serious adverse events (SAEs) will be reported using a standard SAE reporting form. The investigators will report via e-mail or fax all deaths and immediately life threatening SAEs that occur in the course of the study to the safety monitor and sponsor within 24 hours of the investigational site becoming aware of it to the sponsor.

The PI will report the SAE to Research Ethics Committee (REC) of the College of Medicine, Malawi within 48 hours of being notified. The sponsor will be responsible for reporting AEs and SAEs to the Data Safety Monitoring Board (DSMB) in accordance to a predefined schedule suggested by the DSMB. The sponsor will be responsible for notifying the manufacturer of the investigational product in accordance with the requirements of the manufacturer.

Other supporting documentation of the event may be requested and should be provided as soon as possible. Additional information received for a case (follow-up or corrections to the original case) will be detailed on a *full report* SAE form and emailed to the sponsor within 15 days. This information will be reviewed to consider any action that may be needed in response to reported adverse events.

In case of doubt about whether an event fulfils the criteria for expedited reporting, the case will be reported to the safety monitor who will assess whether the event should be reported in an expedited manner.

All SAEs will be followed until satisfactory resolution or until the Principal Investigator deems the event to be chronic or the patient to be stable.

8.11.2 Treatment Stopping Rules

The investigator will judge whether an adverse event is of sufficient severity to require discontinuation of the study treatment. If this occurs, or if the participant wishes to withdraw due to what he/she considers an intolerable adverse reaction, he/she will be offered an end of study assessment and be given appropriate medical care until symptoms cease or the condition becomes stable. If he/she agrees, he/she will continue to be followed up according to schedule.

The trial may be prematurely discontinued by the Sponsor, Chief Investigator or Regulatory Authority on the basis of new safety information or for other reasons given by the Data Monitoring & Ethics Committee / Trial Steering Committee regulatory authority or ethics committee concerned.

The trial may also be prematurely discontinued due to lack of recruitment or upon advice from a Trial Steering Committee, who will advise on whether to continue or discontinue the study and make a recommendation to the sponsor. If the study is prematurely discontinued, active participants will be informed and no further participant data will be collected.

8.12 PARTICIPANTS TIMELINE

8.12.1 Overview Study Phases

The study plan and schedule of assessment is provided Table 2: Study Design and Schedule of Assessment.

It consists of an in-patient pre-study screening period while the patient is acutely ill (approximately -4 to 0 days before enrolment) followed by a screening and enrolment at discharge. It will be during this enrolment visit when the children become formal study participants (t=0). They will also be provided with artemether-lumefantrine (AL) (Coartem®) during the time they in hospital.. During enrolment visit (at discharge), the child will be allocated to one of the 5 study arms. Depending on the arm the mother and child will either return to OPD clinic for PMC drugs at 2, 6 and 10 weeks or be given at discharge to administer them at home also at 2, 6 and 10 weeks. The subjects (a subset) will be visited again at home at 2, 6 and 10 weeks after enrolment to assess DHA uptake for each PMC treatment course. These home visits will be for adherence check purposes, vital registration and health economic assessments only, not for clinical assessment. The PMC period ends at 14 weeks, i.e. 4 weeks after the third PMC course. They will then be requested to come to the clinic at 15 weeks after enrolment for an end of study assessment. Subject's parent or guardian will be instructed to return his/her child to the study clinic for evaluation free of charge at any time their condition warrants medical attention during the 14 weeks follow-up period after discharge.

8.12.2 Pre-screening

Parents / guardians of children who fulfil the pre-screening eligibility criteria will be informed about the study by the hospital or study staff. Consent will not be obtained at the acute stage of the illness but a few hours or days later when the child has recovered and is able to take oral medication and discharged. This provides a time window for the parents or guardian to reflect on the study and discuss it with family members and study staff.

Pre-study screening will be done by hospital staff or study staff. No study specific information or study samples will be collected in this pre-study screening period. However, study staff may be asked to keep any leftover blood volume of routine samples that would otherwise be discarded (e.g. for blood-group typing and cross-matching) in the fridge until consent is obtained at enrolment. The role of the study team during this period is to review the diagnosis and ensure that the potential study participants get standard care for severe malarial anaemia.

Each pre-screened subject will be assigned a pre-screening number in sequential order by the hospital regardless of whether they fulfil the pre-screening eligibility criteria. Data will be recorded on a pre-screening log that will be kept in the investigator’s site file. This record will be used to report how many patients were pre-screened and how many were eventually recruited in the study to establish that the study population was selected without bias. This screening log will not contain names or other identifying information.

8.12.3 Screening interview and consent & Enrolment

8.12.3.1Screening & Consent

The parents of the children will be approached for a screening interview as soon as the child is sufficiently recovered to take oral medication (i.e. after completion of the blood

transfusion and the standard parenteral artesunate medication [if any is indicated]). This is typically within 24 to 48 hours following admission to the hospital. During this interview, consent will be sought from the parents or guardian and if consent is granted, the eligibility criteria for inclusion into the study will be assessed.

8.12.3.2 Screening log

The investigator will keep a subject screening log for all subjects considered for enrolment regardless of whether they were enrolled, which, combined with the pre-screening log will be used to establish that the study sample was selected without bias. This screening log will not contain names or other identifying information.

8.12.3.3 Assignment of study IDs

Eligible subjects will be issued a study subject number during the enrolment visit. Once issued the study subject number they will be considered as 'enrolled.' This number is the subject's unique identifier and used to identify the subject on the CRFs. Subject numbers will be assigned strictly sequentially as subjects enter the study depending on the cluster the participant will be coming from. Once a number has been assigned no attempt will be made to use that number again, for example if a subject discontinues or is a screening failure. Study participants will be randomised to study arms depending on the cluster from which they come from.

8.12.3.4 Clinical assessment:

After consent is obtained and the subject's eligibility is confirmed, the subject's demographic data will be recorded in the CRF, and all relevant clinical information, including the previous and current medical history, and laboratory information copied from the hospital's clinic and laboratory notes to the CRF (see CRF for details). A further clinical examination (including anthropometrics) will be performed and a medical history taken that will serve as the baseline examination and captured on the CRF.

8.12.3.5Baseline Laboratory Measurements

As part of standard of care: Children with severe anaemia are expected to have the following tests done as standard of care before blood transfusion: Malaria smear or RDT, haemoglobin concentration, blood smear to type the anaemia, blood cultures (if patient is febrile and facilities are available), complete blood count haemogram (subject to availability of automated analysers). In addition they are expected to have a blood group and cross match test with donor blood as part of the blood transfusion process. After completion of the blood transfusion, a post transfusion haemoglobin concentration is taken. If the patient was suspected to have malaria, a repeat malaria smear or RDT is often taken to evaluate whether the parasitemia has been cleared.

We shall record all these laboratories results and any others tests results such as urinalysis and radiological results that might have been done as part of standard of care. During the pre-study screening period the study team shall support the hospital to facilitate the routine use of these tests and the recording of clinical and laboratory information on the clinical and laboratory forms and hospital registers to ensure they are available to the study team once the patient is enrolled into the study.

Study specific samples: In addition to the standard of care tests, a venous blood sample will be taken (3 ml) through the existing cannula where possible (in which case no additional 'prick' will be required). In cases a sample cannot be collected through the existing cannula, a finger prick will be performed. This will be used for checking malaria

parasites using microscopy, haemoglobin and a DBS sample on filter paper for genotyping of malaria parasites

HIV testing or collection of information on HIV-exposure (i.e. maternal HIV status) will not be done as a study specific procedures, but the information will be copied from the clinical and laboratory hospital records of patients, including information on the maternal HIV status, where available, or the HIV-status will be obtained following provider initiated testing and counselling (PITC) procedures that are part of routine in-hospital care. Where required the study will support the hospital infrastructure needed to generate this information for the study to ensure this information is available for all study participants.

8.12.3.6 Location of household and issuance of study ID card

A detailed route description to locate the participant's household, including sketching a map, if needed, will be recorded, to aid the planned PMC treatment follow up visits at home. Lastly, a subject's identification card will be issued. A GPS will be used to collect the exact location of the participant's house.

8.12.4 1st, 2nd and 3rd PMC treatment schedules

The 1st, 2nd and 3rd PMC treatment will be given 2, 6 and 10 weeks following enrolment. These treatments will be given at home for those randomised to Arm 1, 2 or 3 and at OPD for those randomised to Arm 3 and 4 (Day 1 dose only, Day 2 and 3 will be at home). PMC uptake will be assessed by unannounced visits on day 4 or 5 after each treatment course by a research assistant. If children are found to be ill during these home-visits they will be referred to the study clinic for further evaluations as part of the unscheduled visits as described below.

8.12.5 Study end visit

Study participants will be asked to return to the study clinic at the hospital at 15 weeks. At this visit a history will be taken and examination performed. A blood sample will be taken for malaria slides, haemoglobin and DBS for malaria parasites genotyping. Additional treatment for malaria and / or anaemia will be given if required. Study staff will visit the house if the family fails to present for this visit at the clinic.

Economic costs will be identified from the patient perspective by asking whether the patient sought any medical treatment, other than at Zomba Central Hospital, during the trial period. If so, costs of travel time, waiting time and associated costs will be identified. In addition, patient intervention costs of time spent receiving the treatment will be identified and any additional costs associated with this, such as special dietary requirements, among others.

8.12.6 **Unscheduled visits (passive follow-up)**

8.12.6.1 Inter-current illness

A passive surveillance system will be used to monitor inter-current illnesses through study-clinics. Parents will be instructed to bring their child to the study clinic for any suspected illness. The 'chief complaint' (reason for attending the clinic during passive surveillance) and 'diagnosis' will be recorded using a standard list based on the International Classification of Disease (ICD-10) for children[18]. Blood samples for malaria smears, parasite genetics (filter paper dried blood spots) and haemoglobin will be taken if clinically indicated (e.g. documented fever >= 37.5 °C axillary or >38.0 °C rectal, or a history of fever in the last 24 hours).

8.12.6.2 Verbal autopsy visit

All parents of children who die will be visited at home as soon as possible for a detailed verbal autopsy interview using standardized questionnaires that will be used to categorize the potential cause of death as probable, possibly, or not malaria related.

9 SUB-STUDIES

9.1 ECONOMIC EVALUATION

The economic evaluation will provide information about incremental costs, incremental health benefits and cost-effectiveness of different delivery strategies of PMC compared current standard of care in Malawi. The overall aims are to inform decision makers whether PMC is likely to be cost-effective if routinely implemented, and which delivery strategy that is likely to be most cost-effective.

A novel decision-model (Fig 2) will be developed to capture aspects relating both to treatment efficacy and relating to country specific implementation characteristics including different implementation costs. Since recurrent follow-up is common, and since previous health events are risk factors for new events, a micro-simulation model is adequate to capture costs and health outcomes. The influence of single parameters will be analysed with one-way sensitivity analyses, while overall decision uncertainties will be estimated utilising probabilistic sensitivity analysis. Incremental costs will be calculated per case averted of severe anaemic malaria, per death averted and per disability adjusted life year (DALY) averted. Treatment efficacy will be based on primary data from this trial, combined with results from the previous RCT in Malawi [2] DALYs will be calculated using standard assumptions from the recent Global Burden of Disease Study.

Figure 2: Decision Model

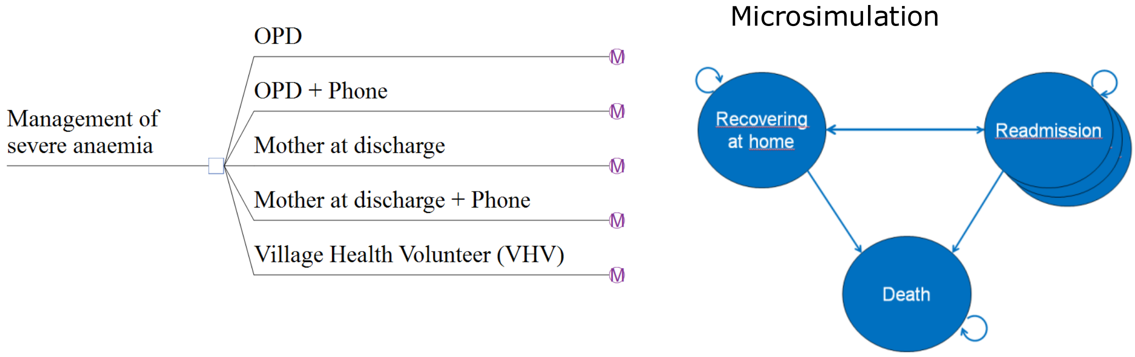

The interventions will be costed prospectively from the perspectives of both the health care provider and the patients/families. Costs will be calculated from a societal perspective, but results will be presented disaggregated for the health care provider and

the patients/families. We will use an ingredients approach, in which resources in separate processes are identified, quantified and valued.

The economic evaluation requires collection of some data in addition to the implementation trial data. The families of the affected children face cost related to receiving the PMC treatment (intervention costs), and in addition they are subject to costs related to the primary disease, readmissions or death (disease costs). Likewise, the health care providers need resources to provide the intervention, which in turn will affect future cases of disease.

9.1.1 Patient intervention costs

The patients' costs of receiving the intervention will be considered in the final study visit. A number of questions will be asked to assess the additional direct and indirect costs associated with receiving the PMC intervention, including travel expenses, treatment expenses and time use for care-takers. Only costs occurring post-discharge will be considered as intervention costs. Time use will be valued assuming that national minimum wage rates represent the alternative cost of the care-takers time.

9.1.2 Patient disease costs

Patients face costs relating to treatment of the primary disease and anaemia, as well as re-admissions or death. Patients' expenses and time use related to the primary disease will be assessed during the enrolment visit or at discharge from the hospital, while their costs related to recurring cases of disease will be considered during the unscheduled sick visits to the study clinic. Both tradable and non-tradable cost items will be identified, measured and valued. Household economic outcomes will be estimated as in the Living Standard Measurement Surveys (www.worldbank.org/lsms).

9.1.3 Provider intervention costs

Provider costs of implementing the five different modes of PMC will utilize information from project accounts, as well as interviews with program managers and health care staff. The data collection will be done retrospectively towards the end of the clinical trial, and efforts will be made to separate intervention costs from costs related to research activities.

9.1.4 Provider disease costs

The assessment of disease treatment costs from the perspective of the health care provider will combine data from facility records and accounts, interviews with health care workers in the hospital and patient cards, and will capture direct and indirect health care provider, capital and recurrent costs. The primary disease and anaemia will be costed prospectively for the recruited children, while re-admissions will be costed prospectively after discharge of patients for all cases retrospectively.

It is increasingly being acknowledged that societal costs are essential in cost-effectiveness analysis; the World Health Organization also recommends this for all analyses conducted. A broader societal perspective is important because the disease not only inflicts costs on the healthcare system, but also puts considerable burdens on the household when an individual falls ill. For the patient there are direct out-of-pocket expenses associated with expenses related to seeking preventive treatment as well as

treatment of re-admission. In addition, the indirect costs related to the value of the time loss spent seeking the treatment both for the patient and the care-taker represent a burden that can be even larger than the directed expenses. Specific objective 5, is thus very important for the analysis as it means including the perspective of the households/patients/families, who are the main benefactors of the intervention. In the following, we will therefore attempt to explain better the costing methods and argue that sub-objective 5 is achievable.

Numerous studies on care seeking costs and the following burdens have been conducted. One relevant example that indicates the feasibility of such measurements is Ewing et al [19]; they conducted a costing study of treatment-seeking and household cost of febrile illness among children in Malawi. This article is co-authored by Prof. Phiri. Additionally, Prof. Robberstad has vast experience in conducting costing studies where the patient perspective is also considered; see f.eks. Ngalseoni et al [20] and Wandwalo, Robberstad and Morkve [21]. Recent experience from Kisumu, Kenya, where another branch of this study is taking place, demonstrates that mothers easily and willingly answer questions about their time use and expenses related to seeking care for malaria disease.

9.2 EQUITY IMPACT ANALYSIS

In addition to important components such as cost-effectiveness, safety and efficiency when considering delivery mechanisms, a consideration of equity is equally valuable. In health we are concerned with its distribution within the population; equity is a term that captures the moral and ethical concern for this distribution. Does delivery mechanism affect compliance based on different socioeconomic variables? Is a specific mechanism superior for a specific part of the population depending on socioeconomic factors or positions? Does one of the delivery mechanisms secure better equity compared to the others? In order to resolve these issues, it is important to detect which determinants, if any, is imperative for the delivery of PMC. This is an effort to incorporate equity into cost-effectiveness, and will be valuable input to decision makers when considering new policies.

In order to conduct an equity impact analysis, an asset index has proven valuable; this is particularly so in areas where income levels might be difficult to define or differentiate by socio-economic group. The analysis can be done by dividing the population into quintiles, based on possession of assets in the household, such as: building materials (in house), water source, radio, bicycle, livestock, acres of land, cooking fuel, food items etc. This information will be collected during the study-end visit.

9.3 QUALITATIVE STUDY ON ACCEPTABILITY AND PERCEPTIONS ON FEASIBILITY

The qualitative study will involve health providers who are involved in the project and carers of children from the five arms. The aim of the study is twofold. The first aim is to understand to what degree health providers and care takers see IPTpd as a useful and welcome intervention that will improve children's health, and to what degree the intervention is seen as being worth the extra effort expected from health workers and carers (acceptability). The second aim of the study is to understand the feasibility of the different delivery mechanisms.

9.3.1 Health providers

Focus Group Discussions (FGDs) will be held with each of the following groups: i) health workers at hospital level, ii) village health workers, iii) health surveillance assistants (HSAs), and iv) District health management teams. For each group, two FGDs with different participants will be conducted in the beginning of the trial, and this will be repeated with the same participants (to the extent possible) when the trial is completed. The total number of FGDs will be 16. A central aim of the FGDs will be to assess the acceptability of IPTpd. In a setting where health providers often work under stress, how do they perceive the additional work burden that IPTpd represents? Do health providers expect additional remuneration, and does the lack of extra compensation have an effect on their motivation to implement IPTpd? To what degree do health providers believe that IPTpd is an important intervention to save children's lives, relative to other interventions? The FGDs will also aim to assess the adaptations that health providers need to make to their working practices in order to implement IPTpd, perceptions of the feasibility of implementing IPTpd through different delivery mechanisms, and recommendations on effective implementation. The information gathered through FGDs will be supplemented by ethnographic methodologies like observations and informal interviews. The FDGs will be conducted in the local language, Chichewa. All FGDs will be recorded, transcribed verbatim, translated to English and coded using software.

9.3.2 Carers of children:

A random selection of carers from the five arms will be conducted when the trial is completed. In each of the five arms, we will select four care takers of children who completed the treatment, and four who did not for in-depth interviews. The care taker will be the person who was with the child during hospitalization and who was recruited to the study. The total number of IDIs will be 40. The care takers will be interviewed at their homes and will be contacted by phone (own or neighbour's) for an appointment. Interview guides will be adapted to fit the issues pertinent to each of the groups (i.e. distance/transport and remembering the dates for OPD delivery, problems with access to working phones for SMS reminders, availability/presence of VHW/HSA etc). We believe that IDIs will be the best method for collecting sensitive and confidential personal information from care takers (including economic constraints, decision-making processes at household level and relationship with Village Health Workers/HSAs and providers at hospital level). In addition to IDIs, two FGDs in each arm will be conducted (one with those who completed the treatment and one with those who did not) to cover less sensitive and confidential themes and to get an idea of common norms. The total number of FGDs with care takers will be 10. Put together, the IDIs and FGDs will explore acceptability of IPTpd and perceptions of different models of delivery including facilitating and blocking factors. We will also map local/non-biomedical perceptions of anaemia which may have an effect on the utilisation of, and trust in, biomedicine. All IDIs and FDGs will be conducted in the local language and will be recorded, transcribed verbatim, translated, and coded using software.

10 Programme Management

10.1 TRIAL AUTHORISATION

Ethical clearance will be sought from the funding country, Norway as well as the Malawian regulator (the College of Medicine Research and Ethics Committee) and the Pharmacies and Poisons Board of Malawi.

10.2 TRIAL STEERING COMMITTEE

Trial Steering Committee will provide independent oversight to ensure the trial progress; adherence to the protocol, patient safety and the integrity of the trial is protected. The TSC members will consist of trial PIs, trial statisticians and 3 independent members, including the independent chair. The TSC will meet every 6 months, virtually or face-to-face. The Data Safety Monitoring board will be appointed comprising of three independent internationally acclaimed experts in malaria and global health (one with statistical expertise).

10.3 TRIAL SPONSORSHIP AND QUALITY ASSURANCE

The College of Medicine of the University of Malawi will be the trial sponsor for the delivery mechanism trial. Trial authorisation will be sought from Pharmacy, Medicines and Poisons Board). Clinical monitoring will be sub-contracted to an independent academic CRO; at least 3 visits are planned in the first year, including study initiation, and then 6-monthly thereafter until trial close out.

10.4 NATIONAL AND INTERNATIONAL COLLABORATION AND NETWORKING

This project will be conducted in collaboration with various institutions. CIH-UIB recently received Centre of Excellence status for its Centre for Intervention Science in Mother and Child Health (CISMAC), with a 10 year 33m USD grant from the Research Council of Norway. The current proposal fits well with CISMAC's vision to 'undertake intervention research to support equitable improvement of maternal, neonatal and child health (MNCH) in low and middle income countries (LMICs) in sub-Saharan Africa'. If funded it may also compete for CISMAC collateral funding. Collaboration with Chr. Michelsens Institute (CMI) brings together the leading Norwegian institute in international health with the leading Norwegian institute in development studies. The project will establish a new, potentially strong South-South partnership between 3 research groups in Africa that are all national leaders in the area. The proposed grant will also enhance the North-South networking capacity by bringing together a number of well-established northern partners from Norway (1) CIH, (2) CMI; Europe (3) LSTM, (4) Imperial College London, UK (5) AMC, Univ of Amsterdam, the Netherlands; and USA (6) Univ of Minnesota. Together this partnership constitutes a new multi-centre trial network in endemic areas in Malawi, Uganda and western Kenya, capable of conducting large-scale definitive trials. Additionally the research sites in Malawi will be part of the Malawi-Zambia-Bergen NORHED proposal research platform that would support masters in epidemiology and research capacity building of the 3 institutions.

10.4.1 Impact of the project and Key beneficiaries

This project is designed to generate the information required to assess whether IPTpd should be recommended as a cost-effective strategy for the post-discharge management of children with severe anaemia in malaria endemic areas. It is anticipated the trial results will increase the effectiveness of the management of severely ill children and reduce the health care costs associated with high readmission rates post-discharge, thereby also reducing associated costs to poor households. Women, who are the primary care takers and also key actors for households economic development, will be relieved and consequently improved welfare for households is expected. In the longer term, the ultimate beneficiaries of this research will be the children in sub-Saharan Africa, whose quality of life, health, welfare and creative output will be enhanced.

WHO and other international stakeholders: The beneficiaries include international organisations and funders of large scale malaria control initiatives such as the Global Fund, DFID, USAID and the US President's Malaria Initiative (PMI), and non-profit organizations such as the Malaria Consortium and MSF, which aim to improve malaria at regional and local levels and are instrumental in supporting countries to implement malaria control policies in sub-Saharan Africa.

National policy makers and stakeholders: Policy makers in Malawi will also benefit directly from the data generated by the trials: not only will they have the necessary country specific data on the safety, efficacy and cost effectiveness of PMC but also valuable information on effective delivery channels. Beneficiaries include the Ministry of Health, responsible for providing hospital services, and the National Malaria Control Programmes responsible for setting policy for malaria control with inputs from local research groups and leadership from WHO. Relevant departments of MOH in the countries are key collaborators in the trials and have been involved from the outset.

Commercial private sector: Sigma Tau, the manufacturer of Eurartesim (R) (the only Good Manufacturing Product [GMP] version of DHP and approved by the European Regular) and the 4 other manufacturers of generic formulations of DHP in China and Vietnam, will benefit from the data on the safety, efficacy and cost effectiveness for a new indication for the drug i.e. for chemoprophylaxis in young children recovering from severe anaemia.

10.5 DIRECT ACCESS TO SOURCE DATA AND DOCUMENTS

The Investigator(s) will permit trial-related monitoring, audits, REC review, and regulatory inspections by providing the Sponsor(s), Regulators and REC direct access to source data and other documents (e.g. patients' case sheets, blood test reports, laboratory reports,).

11 Data Collection, Management and Analysis

The study will either use computer tablets or optical character recognition (OCR) software for creating and scanning (paper-based) data collection forms (Cardiff TeleForm). Completed CRFs and relevant source documents will be scanned on site and transferred daily (encrypted) to a central server at College of Medicine in Blantyre where they will be processed into databases via OCR. Data will be stored in MS Access and SPSS format. CrashPlan-Pro software will ensure automated encrypted back-ups to servers in cloud and at CoM via the internet.

11.1 DATA COLLECTION

Data will be collected and recorded at the point of contact; i.e. either the health facility or at the participant's home by one of the trained study staff and VHV members. Site supervisors will check data collection forms at the end of each day for completeness and accuracy of recording. All uncompleted CRFs will be flagged and accordingly corrected using Good Clinical practices.

11.2 DATA ENTRY AND EDITING

Data will be entered directly into tablets, Personal Digital Assistants (PDAs) or computer touch screens. These electronic data capturing gadgets will be pre-programmed and uploaded with electronic Case Record Forms (eCRFs) using Visual CE programming software. The eCRFs will have cross-checks for verification, validation rules and comply with Good Clinical Practices (GCP). These gadgets will be connected to a desktop allowing data to be directly uploaded into the database. The desktop will be installed with MS SQL server that automatically backs up data. The final data set will be transferred to statistical software programmes used for analyses, e.g, SAS, SPSS and other programmes.

11.3 DATA SHARING

Anonymised data will be available to other researchers for further analysis, subject to ethical approval, the terms of the original participant consent and agreement on its use according to prevailing laws on intellectual property rights at partner institutes. The Partner institutions in the trial would automatically have the right to royalty free use of the data. For others, priority will be given to requests from Malawi. Access to aggregated data will be made readily available, whereas applications for individual participant datasets will be subject to a more stringent review process by the under the authority of the Project Management Committee (PMC) described earlier. The processes will be detailed and agreed in the institutional contracts to be signed by all partners. Information on data availability will be established by provision of an e-mail contact in publications and study reports, and on the project's web-page.

11.4 DATA STORING AND ARCHIVING

Original data collection forms will be handled only by study staff and kept under locked storage until completely coded, checked and transported for data entry. Once data entry and cleaning are complete the original forms will be stored under lock and key at the TRUE until final analyses and reports have been prepared. The CRFs will be stored long term at the TRUE up to a minimum of 7 years. Data will be kept electronically at the TRUE and CoM in compliance with prevailing laws on data storage in all institutions. They will then be destroyed according to true policy and prevailing regulations in Malawi.

11.5 QUALITY ASSURANCE

11.5.1 Quality Control of the trial

Monitoring of this trial will be conducted to ensure compliance with Good Clinical Practice and scientific integrity will be managed and oversight retained, by the sponsor. The Research Support Centre will be responsible for clinical monitoring of the trial on behalf of the sponsor.

GCP training will be provided to all staff/investigators by the Research Support Centre of the College of Medicine, the University of Malawi.

11.5.2 Quality assurance/control of laboratory tests

Regular audits of laboratory performance will be completed by experienced supervisors unconnected to the research study according to standard operating procedures. All malaria blood smears will be read by two different microscopists blinded to the RDT and each other's results, any significantly discordant results based on positive/negative result will be verified by a third expert microscopist. All RDTs will be stored and transported at the recommended temperatures and testing for malaria parasites will be done as per the instructions of the manufacturer.

11.6 DATA HANDLING

The Principal Investigator will act as custodian for the trial data. The following guidelines will be strictly adhered to:

Patient data will be anonymised

- All anonymised data will be stored on a password protected computer.
- All trial data will be stored and archived in Malawi in line with local regulations. Hard copies if any of CRFs will remain on site until completion of field work and stored long term at the Training and Research Unit of Excellence (TRUE)
- Electronic copies of the CRF and database will be archived with limited access.

11.7 STATISTICS AND DATA ANALYSES

11.7.1 Sample Size and randomization

Although a 2x2 factorial design will be used, only the main effects of "place of delivery" and "use of mobile phone reminders" are of primary interest. Each village cluster contributes 2 to 4 children/y with severe anaemia and there are 102 clusters in the catchment area. Assuming an intra-cluster correlation coefficient (ICC) of 0.1 and allowing for 20% loss-to-follow-up or efficiency loss due to varying cluster sizes, a sample size of 19 clusters of 5 children per arm (95 per arm, N=380 overall) has 80% power to detect a 25% absolute increase in coverage from an estimated 50% in the standard VHV or OPD groups to 75% in the arms supported by mobile phone reminders (α=0.05). The ICC of 0.1 is slightly more conservative than the ICC in a previous trial of delivery approaches for IPTc in the Gambia (0.08)[14].

The trial will use villages as the unit of randomisation; i.e. it is a cluster-randomised trial. This trial uses a factorial design and will evaluate two interventions, and will have 4 arms (factorial design). These kind of trials need to take the clustering in variation at village level into account, also called the design effect.

11.7.2 Randomisation procedure

The trial statistician Dr Mavuto Mukaka will computer-generate list of sets of matched clusters and forward these two to the PI. The allocation of clusters to either community-based or health facility-based intervention will occur at a public event prior to the trial. Village elders for 3 matched clusters will be asked to draw one opaque sealed envelope each from a box. Each envelope will contain the allocation. The allocation will be immediately recorded and sent to the study statistician, who in turn will provide the DSMB

statistician with a signed, dated copy in a sealed envelope with carbon paper inside to transfer hand written information onto the list inside the envelopes to create an audit trail.

11.7.3 Protecting against bias

Minimization of selection and confounding bias will be achieved through central block randomisation taking baseline data on malaria risk into account. The matched design with villages as the unit of randomisation will minimize contamination between individual villages and avoid allocation errors.

11.7.4 Data Analysis

The % of children receiving IPTpd according to schedule will be compared by relative risks (95% CI), adjusted for prognostic factors at baseline using log binomial or Poisson regression with adjustment for cluster effects. Hazard ratios will be calculated for morbidity endpoints using Cox regression for repeated events with robust standard error estimation methods to account for correlation between episodes within children. Incidence rates per child-year and absolute rate reductions will also be calculated.

12 Result dissemination and publication policy

This study is part of the activities of the PMC Consortium funded through the Norwegian GLOBVAC programme to conduct these ancillary studies in Malawi, Uganda and Kenya aimed at generating the evidence needed by the World Health Organisation to consider PMC as a strategy to reduce post-discharge morbidity and mortality in malaria endemic areas in Africa.

At the end of the trials, the results will firstly be disseminated to national policy makers, government departments, academics from local research institutions and universities, and professional bodies in Malawi at the College of Medicine Research Dissemination conference as well as specific stakeholder fora. Based on consensus emerging at these meetings, project partners in Malawi will support national policy makers to develop the necessary tools and guidelines to guide national and district level health providers to implement the PMC strategy within hospital services and the health system more broadly.

Research results will also be disseminated to the global malaria research community, technical agencies, and international government bodies via peer reviewed journals and at international scientific fora, including the annual American Society of Tropical Medicine and Hygiene (ASTMH) meeting in 2015 or 2016, the Multilateral Initiative on Malaria (MIM) in 2017, and via meetings at WHO in Geneva comprised of leading scientists in the field of malaria.

We will also inform other international organisations and funders of large scale malaria control initiatives including DFID, USAID and the US President's Malaria Initiative (PMI) which aim to improve malaria at regional and local levels and are instrumental in supporting countries to implement malaria control policies in Africa.

Lastly, the investigators will publish the results of this research scientific peer reviewed journals.

12.1 AUTHORSHIP AND PUBLICATIONS

The study will have a publications committee that will be part of the PMC Consortium created by the partners funded through the Norwegian Globvac programme to conduct this and these ancillary studies in Malawi, Uganda and Kenya aimed at generating the evidence needed by the World Health Organisation to consider PMC/IPTpd as a strategy to reduce post-discharge morbidity and mortality in malaria endemic areas in Africa. The core membership of the Publication Committee will consist of the Consortium grant holders (KP and BR), the Chief Investigator of this trial (FtK), site-principal investigators (Kenya MD and SK, Uganda CJ and RI) from each participating country and the leads of the ancillary studies (BR and MC). For each manuscript a writing committee will be formed. Each participating country group is requested to suggest and justify names for authors in addition to the CI and PIs, to be reviewed by the publications committee. Potential site authors could include all professionals that have participated in the trial for a minimum of one year. It is anticipated that for the publication of the results of the main trial, the CI will have last authorship and that a PhD student or one of the site PIs will have lead authorship. It is also anticipated that the PI of an ancillary study should be considered for the first choice for lead or last author of material derived from this study. Authorship of any presentations or publications arising from this study will also be governed by the principles for authorship criteria of the International Committee of Medical Journal Editors has designed. Disputes regarding authorship will be settled by the CI, PIs and chair of the publications committee.

12.2 INSURANCE / INDEMNITY

A trial insurance policy will be taken out by the sponsor in country.

12.3 FINANCIAL ASPECTS

Funding to conduct the trial is provided by the Globvac programme, the Research Council of Norway, project identification 234487.

13 Capacity building

13.1 TRAINING, FELLOWSHIPS AND CAPACITY BUILDING

Research capacity will be enhanced by provision of training and mentorship for research staff. By running this trial, capacity in trial management will be enhanced in addition to which north partner staff will provide support to write new proposals for research funding for beyond the end of the trial. The research study will strengthen the clinical skills of health workers in managing patients.

CIH has for many years spearheaded Norway's efforts in global health research, and has conducted high-impact research in the area of child health. Over the last 5 years, CIH successfully trained 55 MSc students and 112 students completed their PhD training at the research school in international health (www.uib.no/rs/ih), (50 the last 5 years). There will be 1 PhD candidate who will conduct his/her research as part of this project with a thematic area in epidemiology, and/or medical statistics. Partners from the different institutions forming this research network will jointly supervise them. They will be required to present their scientific work during PMC meetings where their general academic progress will be discussed. There is dedicated PhD space at the College of

Medicine with internet and library facilities. The field site will have dedicated area for PhD and other students with dedicated internet facilities. The PhD will have small funds to support educational materials and travel to conferences.

13.2 AFRICAN LEADERSHIP

This program will offer the PI (PhD qualified 6 years ago) the opportunity to learn to lead a large multi-country multi-disciplinary project supported by Profs ter Kuile and Robberstad, and will offer the opportunity for Dr Mavuto Mukaka, the study statistician to get exposure to multi-centre clinical trials. He will benefit from the involvement of Dr Cairns (LSHTM) and the trial statistician at CDC. A Norwegian Post-doc will benefit from a 1-year assignment at the Imperial College London and LSHTM.

14 Ethics & Regulatory Approvals

14.1 DECLARATION OF HELSINKI

The trial will be conducted in compliance with the principles of the Declaration of Helsinki (1996), the principles of GCP and in accordance with all applicable regulatory requirements in Malawi.

14.2 RESEARCH ETHICS COMMITTEE AND INSTITUTIONAL REVIEW BOARDS

This protocol and related documents (informed consent and participant information sheets) will be submitted for review to the Institutional Review Boards (IRB) and Research Ethics Committees at the Malawi College of Medicine.

Any protocol amendments will be submitted to the primary ethics committees before implementation. The study will comply with local regulations pertaining to reporting of SAEs and in addition will report safety data to Sigma Tau, the manufacturer of DHP. A copy of the final study report will be provided to all RECs, DMEC, regulator, and Sigma Tau.

14.3 INFORMED CONSENT

Informed consent will be obtained from each participant and documented appropriately before children are enrolled in the study (see appendix). The carers will be given the informed consent form to read, with the help of the trial staff, and the carers will be given a chance to ask questions. The carers will be given a copy of the consent form. Carers who are able to sign their name will be asked to sign the consent form. For a carer who cannot read (non-literate), the informed consent will be read to the carer by a member of the study team, and documented by thumbprint. Carers who consent using a thumbprint will have a witness who attests the consent was read to them in its entirety, and that their participation was voluntary. Details about the trial and its benefits and potential risk will be explained to the participants in the language in which they are most fluent.

14.4 PROTECTION OF PRIVACY AND CONFIDENTIALITY

14.4.1 Privacy of individual

Individual data such as positive RDT tests for malaria and anaemia will be reported to the participant at point of care, to relevant study staff, the antenatal care doctor where appropriate and will be recorded in the child's health passport in addition to study CRFs.

14.4.2 Confidentiality of data

All information regarding the participants will remain confidential to the extent allowed by law. Unique numerical identifiers will be used for data entry. All screening forms and case report forms will be kept in a secured location with access limited to authorized study staff. Unique numerical identifiers will be used for the computer-based data entry and blood samples. Publications will contain only aggregate data; no identifying information will be included.

14.5 ETHICAL CONSIDERATIONS FOR HUMAN PARTICIPANTS

14.5.1 Risks from blood sampling

Blood sampling may be inconvenient to the participants, and may cause minor discomfort and bruising. In some aspects of the trial, blood sampling has the potential to directly benefit the participants as any malaria infection or anaemia detected as a result of the sampling will be treated.

The volume of blood collected from each participant will be small; a maximum of 10 mls per child may be collected over the course of the study. Well-trained nurses and laboratory staff employed on the trial will perform blood-sampling tasks. New and sterile disposable needles and lancets will be used for blood taking procedures. Universal precaution measures for blood handling and disposal will be observed when performing the procedures and used needles and other waste will be safely discarded immediately after use.

14.5.2 Safety of study drug in children

DHP is currently thought to be safe in children. However, adverse events, particularly those associated with the study medication will be recorded and monitored throughout.

14.5.3 Methods to minimise risks

In order to minimize risks to study subjects, subjects will be encouraged to present to the clinic at any time that the they show signs of any illness, fever, or injury. Sterile technique will be used to obtain blood samples and only trained and experienced staff will perform finger prick and venepuncture.

14.5.4 Experience with DP as PMC

Dihydroartemisinin-piperaquine (DP), the drug combination that will be used in this trial, is one of the artemisinin containing combination therapies (ACTs). ACTs are now the standard for treatment of *P.falciparum* malaria in both adults and children [22]. A systematic review of the efficacy and safety of ACTs for the treatment of malaria in children conducted to inform the 2nd edition of the malaria treatment guidelines by WHO, showed that DP is very effective and provides a long duration of post-treatment

prophylaxis, similar to mefloquine and longer than amodiaquine (AQ-artesunate, or AQ-SP) and artemether-lumefantrine (Coartem®) based antimalarial combinations [23].

Because of its long half-life, piperaquine (PQ) has great potential for use as the ACT of choice for Intermittent Screening and Treatment (IST) and IPT, as was shown in IPTc studies in children [24].

Furthermore a trial with monthly DP used as IPT in Thai adults showed it to be well tolerated, safe, and highly effective [8]. In that trial the most important determinant of protective efficacy was the trough plasma concentration of piperaquine, and this was determined by the dosing frequency. Although an acceptable protective efficacy of 86% was achieved with dosing every 2 months, the drug concentrations required for successful prevention of malaria infections are not maintained in between doses. Compared with participants receiving monthly DP dosing, participants who received dosing every 2 months were 8 times more likely to get malaria (adjusted hazards ratio [AHR], 8.24; 95% CI, 3.25 to 20.9), and participants in the placebo group were 41 times more likely to get malaria within 9 month (AHR, 41.3; 95% CI, 16.6 to 102.8). This suggests that for effective prevention of malaria, DP should be given monthly in order to achieve steady state concentrations above the minimum inhibitory concentrations and sustained prophylactic levels [8].

PQ is currently only available in the fixed dose combination with dihydro-artemisinin (DHA) as DP. The DHA component, eliminated within a few hours, is not expected to provide a significant contribution to the effect of IPTp, yet may provide a degree of protection against the development of PQ resistance in the population. A GMP formulation of DP has recently become available as Eurartesim, manufactured by Sigma-Tau, Italy and approved in October 2011 by the European Medicine Agency (EMEA) for treatment of malaria

Piperaquine is well tolerated. Side effects in adults include transient drops in haemoglobin by day 7 (seen with all artemisinins), headache, weakness and fever. The main safety concerns with piperaquine relate to its dose-dependent QTc prolongation. Mild levels of QTc prolongation has been confirmed in clinical trials, but these were mild and similar to many other anti-malarials [25] and there is no indication from clinical data signalling that it is associated with clinically significant arrhythmias [26] This is consistent with recently in-vitro models which confirmed that despite mild QTc prolongation the potential cardiac proarrhythmic risk with piperaquine is low and similar to that observed with lumefantrine (the long-acting component in Coarterm), and lower than for chloroquine. This study concluded that DP does not appear to induce potential torsadogenic effects in vitro (which could result in life threatening abnormality of heart rhythm).

14.5.5 **Anticipated benefits to study participants**

By taking part in this trial, participants will receive PMC-DHP in whom we expect to treat infections that may otherwise remain undetected. Participants who do not attend scheduled appointment will receive reminders and active follow-up. Participants experiencing illness between visits will be seen and treated free of charge as part of the study.

14.5.6 **Benefit to the community**

This project is designed to generate the information required to assess whether PMC should be recommended as a cost-effective strategy for the post-discharge management

of children with severe anaemia in malaria endemic areas. It is anticipated the trial results will increase the effectiveness of the management of severely ill children and reduce the health care costs associated with high readmission rates post-discharge, thereby also reducing associated costs to poor households. Women, who are the primary care takers and also key actors for household's economic development, will be relieved and consequently improved welfare for households is expected. In the longer term, the ultimate beneficiaries of this research will be the children in sub-Saharan Africa, whose quality of life, health, welfare and creative output will be enhanced.

14.5.7 Reimbursement of costs

The study will provide payment for all study drugs, study procedures, study-related visits and reasonable medical expenses that are incurred as a result of the study including expenses for transport for any study related visits.

14.6 Ancillary and post-trial care

14.6.1 Health care during the trial

All care directly related to the proper and safe conduct of the trial, and the treatment of immediate adverse events related to trial procedures will be provided free of charge by the local hospitals. The provision of ancillary care beyond that immediately required for conduct of the trial will not be covered by the trial. The use of a health passport or national equivalent will aid the identification of study children.

14.6.2 Trial insurance

The sponsor will take out trial insurance such that participants enrolled into the study are covered by indemnity for negligent harm and non-negligent harm associated with the protocol. This will include cover for additional health care, compensation or damages whether awarded voluntarily by the Sponsor, or by claims pursued through the courts. The liability of the manufacturer of the trial drug DP is limited to those claims arising from faulty manufacturing of the commercial product and not to any aspects of the conduct of the study.

14.6.3 Post-trial care

The study budget is not in a position to fund post-study care or implementation of PMC as policy. However, the investigators work in close collaboration with local and international policy makers (e.g. WHO) and funders (e.g. President Malaria Initiative) to ensure that policy makers and funders are informed early of germane research finding and can plan for the potential implementation of PMC as policy in the study areas and other relevant areas in Malawi and the region.

15 REFERENCES

1. Calis J.C., P., K.S., Faragher, E.B., Brabin, B.J., Bates, I., Phiri, A.I., et, al., *Severe anemia in Malawian children.* The New England Journal of Medicine, 2008. **358**(9): p. 888-99.
2. Phiri, K., Esan, M., van Hensbroek, M.B., Khairallah, C., Faragher, B., ter Kuile, F.O., *Intermittent preventive therapy for malaria with monthly artemether-lumefantrine for the post-discharge management of severe anaemia in children aged 4-59 months in southern Malawi: a multi-centre, randomised, placebo-controlled trial.* Lancet Infect Dis, 2012. **12**(3): p. 191-200.

3. Bojang, K., Palmer A., Boele van hensbroek, M. Banya, W.A., gREENWOOD, b.m., *Management of severe malarial anaemia in Gambian children following discharge from hospital.* Trans R Soc Trop Med Hyg, 1997. **91**(5): p. 557-61.
4. Bojang, K.A., Milligan, P.J., Conway, D.J., Sisay-Joof, F., Jallow, M., Nwakanma, D.C., et, al.,, *Prevention of the recurrence of anaemia in Gambian children following discharge from hospital.* PLoS One, 2010. **5**(6): p. E11227.
5. White, N.J., *Intermittent presumptive treatment for malaria* PLoS One, 2005. **2**(1): p. e3.
6. Phiri, K.S., Calis, J.C., Faragher, B., Nkhoma, E., Ng'oma, K., Mangochi, B, et, al., *Long term outcome of severe anaemia in Malawian children.* PLoS One, 2008. **3**(8): p. E2903.
7. Price, R.N., Simpson, J.A., Nosten, F., Luxemburger, C., Hkirjaroen, L. ter Kuile, F., et, al., *Factors contributing to anaemia after uncomplicated falciparum malaria.* Am J Trop Med Hyg, 2001. **65**(5): p. 614-22.
8. Lwin, K.M., Phyo, A.P., Tarning, J., Hanpithakpong, W., Ashley, E.A., Lee S.J., et, al., *Randomized, Double-Blind, Placebo-Controlled Trial of Monthly versus Bimonthly Dihydroartemisinin-Piperaquine Chemoprevention in Adults at High Risk of Malaria.* Antimicrobial Agents and chemotherapy, 2012. **56**(3): p. 1571-7.
9. Bojang, K., Akor, F., Conteh, L., Webb, E., Bittaye, O., Conway, D.J., et,al., *Two strategies for the delivery of IPTc in an area of seasonal malaria transmission in the Gambia: a randomised controlled trial.* PLoS One, 2011. **8**(2): p. E1000409.
10. Steketee, R.W., Slutsker, L., *Targetting of Intermittent preventive treatment for malaria.* The Lancet Infectious diseases, 2011. **12**(3): p. 168-9.
11. Eldridge SM, A.D., Kerry S. , *Sample size for cluster randomized trials: effect of coefficient of variation of cluster size and analysis method.* Int J Epidemiol 2006. **35**: p. 1292-300.
12. Manatunga A, H.M., Chen S. , *Sample size estimation in cluster randomized studies with varying cluster size.* Biometrical Journal, 2001. **43**: p. 75-86.
13. Kang S, A.C., Jung S. , *Sample size calculation for dichotomous outcomes in cluster randomization trials with varying cluster size.* Drug Information Journal 2003. **37**: p. 109-14.
14. Bojang, K., et al.,, *Two strategies for the delivery of IPTc in an area of seasonal malaria transmission in the Gambia: a randomised controlled trial.* PLoS Med, 2011. **8**(2).
15. Phiri, K., et al., *Intermittent preventive therapy for malaria with monthly artemether-lumefantrine for the post-discharge management of severe anaemia in children aged 4-59 months in southern Malawi: a multicentre, randomised, placebo-controlled trial.* Lancet Infect Dis, 2012. **12**(3): p. 191-200.
16. Calis, J.C., et al., *Severe anemia in Malawian children.* The New England journal of medicine, 2008. **358**(9): p. 888-99.
17. Phiri, K.S., et al., *Long term outcome of severe anaemia in Malawian children.* PLoS One, 2008. **3**(8): p. e2903.
18. WHO, *International Classification of Diseases and related health problems 10th Revision.* World Health Organisation, Geneva, Switzerland., 2015.
19. Ewing, V., et al., *Seasonal and geographic differences in treatment-seeking and household cost of febrile illness among children in Malawi.* Malaria Journal, 2011. **10**(1): p. 32.
20. Ngalesoni, F., et al., *Economic cost of primary prevention of cardiovascular diseases in Tanzania.* Health Policy and Planning, 2014.
21. Wandwalo, E., B. Robberstad, and O. Morkve, *Cost and cost-effectiveness of community based and health facility based directly observed treatment of tuberculosis in Dar es Salaam, Tanzania.* Cost Effectiveness and Resource Allocation, 2005. **3**(1): p. 6.
22. WHO, *Guidelines for the treatment of malaria.* World Health Organisation, Geneva, Switzerland., 2010. **2nd Edition**.
23. Sinclair, D., Zani, B., Donegan, S., Olliaro, P., Garner, P., *Artemisinin-based combination therapy for treating uncomplicated malaria.* Cochrane Database Syst Rev, 2009. **3**(CD007483).

24. Cisse, B., Cairns, M., Faye, E., et, al., *Randomized trial of piperaquine with sulfadoxine pyrimethamine or dihydroartemisinin for malaria intermittent preventive treatment in children.* PLoS One, 2009. **4**(9): p. e7164.
25. Mytton OT, A.E., Peto L, et al. , *Electrocardiographic safety evaluation of dihydroartemisinin piperaquine in the treatment of uncomplicated falciparum malaria.* he American journal of tropical medicine and hygiene, 2007. **77**(3): p. 447-450.
26. GM., K., *Dihydroartemisinin/Piperaquine: A Review of its Use in the Treatment of Uncomplicated Plasmodium falciparum Malaria.* Drugs, 2012. **72**(7): p. 937-961.

17 FINANCIAL ASPECTS AND CONFLICT OF INTEREST

17.1 Funding of the trial

Funding to conduct the trial is provided by the University of Bergen, Norway through a grant from the Research Council of Norway, Global Health and Vaccination Research (GLOBVAC) programme.

GLOBVAC had no role in the design of this trial and will not have any during the execution, analysis, interpretation of the data, or decision to submit the results

17.2 Provision of the study drugs

Dihydroartemisinin-piperaquine will be provided free of charge by the Sigma Tau, the manufacturer. The study will provide copies of safety reports of SAEs and AEs to the manufacturer (expedited where required). The manufacturer will not be involved in the design of the trial.

18 BUDGET AND BUDGET JUSTIFICATION

18.1 Budget Justification

Personnel: There will be a research team that will be responsible for implementing the study in Zomba. This will be lead by the PI and will include Trial Manager and Study Coordinator. They will be responsible for oversight and management of the trial. Nurses and clinicians will be responsible for participant recruitment and follow-up. Research assistants will carry out the community-based visits and lab technician will be responsible for laboratory assays.

Equipment: There will be need for a study vehicle to drop off participants and map exact locations of participants. A research assistant will later use the motorbike to carry out community-based visits. Computer and other related equipment will be used for data capture, management and analysis. There will also be need for small laboratory and clinic equipment for the research site

Consumables: The main consumables are related to use of the vehicles for follow-up (fuel and maintenance of 4x4 vehicle and motorbike). Some consumables have been set aside to support the office (communications, stationary) and laboratory.

Trial Management: No fault insurance will be procured from COMREC-approved insurance agents for all study participants. Support will also be provided to the College of Medicine to support its sponsor role. The funders have kindly agreed to allow the CoM to use to build sponsor capacity, which may include hiring of competent personnel, placement of personnel in other institutions and infrastructure support within the RSC Clinical Trials Unit.

Overheads: To note that the funders only allow overheads on Salaries

20 APPENDICES

20.1 APPENDIX I. ROLE INVESTIGATORS AND NON-ENGAGED COLLABORATORS

20.1.1 Protocol development: authors' contributions

Feiko ter Kuile (FtK) and Kamija Phiri (KP) conceived the study. Nyanyiwe Mbeye (NM), Bjarne Robberstad (BR), S and KP drafted the protocol. BR, Richard Idro (RI), Robert Opoka (RO), Chandy John (CJ), Meghna Desai (MD), Simon Kariuki (SK), Azra Ghani (AG), Michael Boele van Hensbroek (MBvH) and FtK and KP, further development the study design during a protocol workshop. Siri Lange (SL) and BR developed the qualitative and economic evaluation main activities, respectively. KP and BR are the GLOBVAC grant holders. Brian Faragher (BF) provided statistical expertise in clinical trial design. All authors contributed to the refinement of the study protocol and approved the final version.

20.1.2 Role Investigators

Dr Kamija Phiri, a senior clinical epidemiologist from the College of Medicine (CoM) in Malawi with over 10 years' experience in clinical research will be provide technical support to the design and conduct of the study and will conduct a delivery mechanism trial in Malawi. Dr Thandile-Nkosi-Gondwe will work with Dr K. Phiri as the Principal Investigator for the Malawi trial.

Prof Feiko ter Kuile from Liverpool School of Tropical Medicine (LSTM), Dr Meghna Desai (epidemiologist from CDC based at KEMRI), Prof Chandy John (Paediatrician from the Univ of Minnesota with links with Makerere Univ) together Drs Richard Idro and Robert Opoka are investigators on this study, providing scientific input as part of the Trial Steering Committee.

Dr Richard Idro (Makerere Univ.) will be a supervisor for the Ugandan PhD student together with Dr Robert Opoka and Prof Michael Boele (Univ. of Amsterdam). Statistical support will be provided by Dr Mavuto Mukaka (CoM) and the CDC statistician based at KEMRI in Kenya. The scientific lead for Modelling work (data from both Malawi and Kenya studies) will be Dr Michael Cairns from the London School of Tropical Medicine and Hygiene supported by Dr Azra Ghani from the Imperial College.

Dr Martias Joshua, Zomba Central Hospital Director and Dr Doreen Ali of National Malaria Control Program in Malawi will be responsible for the linkage with local control programmes.

Prof Robberstad (Project Manager) of the Project Owner Univ. of Bergen (UIB) who will be responsible for health economics component.

20.1.3 Role Non-Engaged Collaborators

Non-engaged collaborators are not classified as investigators and have an advisory role only and are not to be directly involved with the research activities.

20.2 APPENDIX II. TERMS OF REFERENCE OVERSIGHT COMMITTEES

20.2.1 Trial Management Group (TMG)

20.2.1.1 Purpose

The TMG is responsible for the day to day running of the trial.

20.2.1.2 Membership

1. Chief Investigator (Chair)
2. Principal Investigator,
3. Site clinicians
4. Trial Co-ordinator
5. Administrators
6. Others who are involved in the day to day running of the trial.

20.2.1.3 Responsibilities:

- Study planning
- Organisation of Trial Steering Committee and Data Monitoring and Ethics Committee (DMEC) meetings
- Provide risk report to regulators, manufacture and ethics committees
- SUSAR [Serious unexpected suspected adverse events] reporting
- Responsible for trial master file
- Budget administration and contractual issues
- Advice for lead investigators
- Organisation of central data management and sample collection

20.2.2 Trial Steering Committee (TSC)

20.2.2.1 Purpose

The purpose of this document is to provide the TSC with a guidance of the terms of reference with the understanding that the Committee carry out their functions.

Attend regular meetings that shall be scheduled by the Trial Manager to address points 1, 3, 4 and to track the progress of the trial

20.2.2.2 Membership TSC

Independent members

1. Chair: [name and contact details]
2. Statistician: [name and contact details]
3. Paediatrician/medical officer [name and contact details]

Trial members

- The CI and all Site Co-PIs.

- Other co-investigators and the trial statistician will attend the meetings if and when required.

20.2.2.3Roles and Responsibilities TSC

The TSC is a trial governing body, which includes a majority of its members who are independent of the trial management group. The TSC concentrates on the progress of the trial and ensures that the trial is conducted to the standards set out in the Guidelines for Good Clinical Practice with consideration given to participant safety and provision of informed consent.

- To evaluate the progress of the trial in relation to timeliness, data quality and other factors that can affect the overall objectives of the trial
- To ensure participant rights and safety are adhered to and that the protocol demands freely given informed consent
- To review relevant information from other sources
- To consider the recommendations of the Data Monitoring and Ethics Committee (DMEC) and in light of it to inform the Chief Investigator and TMG the need to changes to the trial protocol
- To inform the GlobVac programme on the progress of the trial and in exceptional circumstances to extend or to terminate the trial.
- To ensure that the trial results are disseminated appropriately and consideration be given to the implementation of the results into policy

20.2.2.4Operational TSC

The CI will present the full protocol to the TSC as an agenda before the start-up of data collection. The TSC members shall review the time line set out in the protocol for participant recruitment, informed consent documents and plans for data safety monitoring.

The TSC shall see that the finalised protocol is sent to the sponsor and funders before the start of participant recruitment and data collection.

The TSC in its first meeting shall approve the nominated members of the DMEC and establish the DMEC which shall meet regularly to review and report on the data quality and the results of interim analyses.

In all their deliberations the TSC should consider any deviations from the trial protocol, participant safety and information provided to the participants and consenting procedures.

20.2.2.5Frequency of Meetings

The TSC shall have an initial face to face start up meeting to discuss the protocol and establish the DMEC. A second meeting shall take place before the initiation of the trial to finalise the protocol and approve the commencement of the trial. Thereafter the TSC will normally meet once a year in the life span of the trial and one meeting at the closure of the trial.

The Chair and at least 2 of the three independent members together with the CI and trial co-ordinator shall constitute the quorum. If so required, in addition a member of the funder can be invited to attend the meetings.

20.2.2.6Trial Reports and actions TSC meeting

The TSC shall provide at each meeting a summary report of their findings and recommendations which must be submitted to the funder, the Sponsor and the TMG.

If the TSC makes a recommendation that the trial should be stopped or suspended, the Sponsor will take the necessary action to ensure that new recruitment to the trial is stopped whilst the TSC report is evaluated and the Research Ethics Committee is informed.

20.2.3 **Data Monitoring and Ethics Committee (DMEC)**

20.2.3.1Membership DMEC

1. Chair: [name and contact details]
2. Statistician: [name and contact details]
3. Paediatrician/medical officer [name and contact details]

20.2.3.2Role DMEC

The DMEC consist of 3-4 members (including one or more clinicians and one statisticians, all with experience in clinical trials).

The DMEC shall assess the data regularly (before the annual TSC meeting) to review the data and the interim analysis. The assessment could be via email or other electronic medium annually prior to the TSC meeting. In the first year of recruitment more frequent assessment (bi-annually) is recommended for this trial with one face to face meeting atleast once during the trial.

The members should be the only personnel to see the results separated by treatment group during the trial. They are independent and look at the trial from an ethical point of view of the participant safety, future patients and society in general. It is their responsibility to prevent patients being exposed to any excess risks by recommending to the Trial Steering Committee (TSC) for the trial suspension or termination early if the safety or efficacy results are sufficiently convincing. The trial statistician is usually invited to attend part of the DMEC meeting to present the most current unblinded data from the trial.

20.2.3.3Responsibilities DMEC

- To determine how frequently interim analysis of trial data should be undertaken.
- To consider the unblinded interim data from the trial and relevant information from other sources.
- To consider any requests for unblinding and release of interim trial data and to recommend to the TSC on the importance of this.
- To report (following each DMEC meeting) to the TSC and to recommend whether the trial should continue, the protocol be modified or the trial be stopped.

A full confidence report should be submitted in writing to the TSC at the end of each DMEC meeting

20.3 APPENDIX III. DECLARATION OF HELSINKI

WORLD MEDICAL ASSOCIATION DECLARATION OF HELSINKI

Recommendations guiding physicians in

Biomedical research involving human subjects

Adopted by the 18th World Medical Assembly, Helsinki

, Finland, June 1964,

Amended by the 29th World Medical Assembly, Tokyo, Japan, October 1975,

35th World Medical Assembly, Venice, Italy, October 1983

41st World Medical Assembly Hong Kong, September 1989

and the

48th General Assembly, Somerset West, Republic of South Africa,

October 1996

INTRODUCTION

It is the mission of the physician to safeguard the health of the people. His or her knowledge and conscience are dedicated to the fulfillment of this mission.

The Declaration of Geneva of the World Medical Association binds the physician with the words, "The health of my patient will be my first consideration", and the International Code of Medical Ethics declares that, "A physician shall act only in the patient's interest when providing medical care which might have the effect of weakening the physical and mental condition of the patient."

The purpose of biomedical research involving human subjects must be to improve diagnostic, therapeutic and prophylactic procedures and the understanding of the aetiology and pathogenesis of disease.

In current medical practice most diagnostic, therapeutic or prophylactic procedures involve hazards. This applies especially to biomedical research.

Medical progress is based on research which ultimately must rest in part on experimentation involving human subjects.

In the field of biomedical research a fundamental distinction must be recognized between medical research in which the aim is essentially diagnostic or therapeutic for a patient, and medical research, the essential object of which is purely scientific and without implying direct diagnostic or therapeutic value to the person subjected to the research.

Special caution must be exercised in the conduct of research, which may affect the environment, and the welfare of animals used for research must be respected.

Because it is essential that the results of laboratory experiments be applied to human beings to further scientific knowledge and to help suffering humanity, the World Medical Association has prepared the following recommendations as a guide to every physician in biomedical research involving human subjects. They should be kept under review in the future. It

must be stressed that the standards as drafted are only a guide to physicians all over the world. Physicians are not relieved from criminal, civil and ethical responsibilities under the laws of their own countries.

Basic principles

1. Biomedical research involving human subjects must conform to generally accepted scientific principles and should be based on adequately performed laboratory and animal experimentation and on a thorough knowledge of the scientific literature.
2. The design and performance of each experimental procedure involving human subjects should be clearly formulated in an experimental protocol which should be transmitted for consideration, comment and guidance to a specially appointed committee independent of the investigator and the sponsor provided that this independent committee is in conformity with the laws and regulations of the country in which the research experiment is performed.
3. Biomedical research involving human subjects should be conducted only by scientifically qualified persons and under the supervision of a clinically competent medical person. The responsibility for the human subject must always rest with a medically qualified person and never rest on the subject of the research, even though the subject has given his or her consent.
4. Biomedical research involving human subjects cannot legitimately be carried out unless the importance of the objective is in proportion to the inherent risk to the subject.
5. Every biomedical research project involving human subjects should be preceded with careful assessment of predictable risks in comparison with foreseeable benefits to the subject or to others. Concern for the interests of the subject must always prevail over the interests of science and society.
6. The right of the research subject to safeguard his or her integrity must always be respected. Every precaution should be taken to respect the privacy of the subject and to minimize the impact of the study on the subject's physical and mental integrity and on the personality of the subject.
7. Physicians should abstain from engaging in research projects involving human subjects unless they are satisfied that the hazards involved are believed to be predictable. Physicians should cease any investigation if the hazards are found to outweigh the potential benefits.
8. In publication of the results of his or her research, the physician is obliged to preserve the accuracy of the results. Reports of experimentation not in accordance with the principles laid down in this Declaration should not be accepted for publication.
9. In any research on human beings, each potential subject must be adequately informed of the aims, methods, anticipated benefits and potential hazards of the study and the discomfort it may entail. He or she should be informed that he or she is at liberty to abstain from participation in the study and that he or she is free to withdraw his or her consent to participation at any time. The physician should then obtain the subject's freely-given informed consent, preferably in writing.
10. When obtaining informed consent for the research project, the physician should be particularly cautious if the subject is in a dependent relationship to him or her or may consent under duress. In that case the informed consent should be obtained by a physician who is not engaged in the investigation and who is completely independent of this official relationship.

11. In case of legal incompetence, informed consent should be obtained from the legal guardian in accordance with national legislation. Where physical or mental incapacity makes it impossible to obtain informed consent, or when the subject is a minor, permission from the responsible relative replaces that of the subject in accordance with national legislation. Whenever the minor child is in fact able to give a consent, the minor's consent must be obtained in addition to the consent of the minor's legal guardian.
12. The research protocol should always contain a statement of the ethical considerations involved and should indicate that the principles enunciated in the present Declaration are complied with.

Medical research combined with professional care (Clinical Research)

1. In the treatment of the sick person, the physician must be free to use a new diagnostic and therapeutic measure, if in his or her judgement it offers hope of saving life, re-establishing health or alleviating suffering.
2. The potential benefits, hazards and discomfort of a new method should be weighed against the advantages of the best current diagnostic and therapeutic methods.
3. In any medical study, every patient - including those of a control group, if any - should be assured of the best proven diagnostic and therapeutic method. This does not exclude the use of inert placebo in studies where no proven diagnostic or therapeutic method exists.
4. The refusal of the patient to participate in a study must never interfere with the physician-patient relationship.
5. If the physician considers it essential not to obtain informed consent, the specific reasons for this proposal should be stated in the experimental protocol for transmission to the independent committee (I,2).
6. The physician can combine medical research with professional care, the objective being the acquisition of new medical knowledge, only to the extent that medical research is justified by its potential diagnostic or therapeutic value for the patient.

Non-therapeutic biomedical research involving human subjects (Non-clinical biomedical research)

1. In the purely scientific application of medical research carried out on a human being, it is the duty of the physician to remain the protector of the life and health of that person on whom biomedical research is being carried out.
2. The subjects should be volunteers -- either healthy persons or patients for whom the experimental design is not related to the patient's illness.
3. The investigator or the investigating team should discontinue the research if in his/her or their judgement it may, if continued, be harmful to the individual.
4. In research on man, the interest of science and society should never take precedence over considerations related to the well-being of the subject.
